# Is My Network Module Preserved and Reproducible?

**DOI:** 10.1371/journal.pcbi.1001057

**Published:** 2011-01-20

**Authors:** Peter Langfelder, Rui Luo, Michael C. Oldham, Steve Horvath

**Affiliations:** 1 Department of Human Genetics, University of California, Los Angeles, Los Angeles, California, United States of America; 2 Departments of Human Genetics and Biostatistics, University of California, Los Angeles, Los Angeles, California, United States of America; University of California San Diego, United States of America

## Abstract

In many applications, one is interested in determining which of the properties of a network module change across conditions. For example, to validate the existence of a module, it is desirable to show that it is reproducible (or preserved) in an independent test network. Here we study several types of network preservation statistics that do not require a module assignment in the test network. We distinguish network preservation statistics by the type of the underlying network. Some preservation statistics are defined for a general network (defined by an adjacency matrix) while others are only defined for a correlation network (constructed on the basis of pairwise correlations between numeric variables). Our applications show that the correlation structure facilitates the definition of particularly powerful module preservation statistics. We illustrate that evaluating module preservation is in general different from evaluating cluster preservation. We find that it is advantageous to aggregate multiple preservation statistics into summary preservation statistics. We illustrate the use of these methods in six gene co-expression network applications including 1) preservation of cholesterol biosynthesis pathway in mouse tissues, 2) comparison of human and chimpanzee brain networks, 3) preservation of selected KEGG pathways between human and chimpanzee brain networks, 4) sex differences in human cortical networks, 5) sex differences in mouse liver networks. While we find no evidence for sex specific modules in human cortical networks, we find that several human cortical modules are less preserved in chimpanzees. In particular, apoptosis genes are differentially co-expressed between humans and chimpanzees. Our simulation studies and applications show that module preservation statistics are useful for studying differences between the modular structure of networks. Data, R software and accompanying tutorials can be downloaded from the following webpage: http://www.genetics.ucla.edu/labs/horvath/CoexpressionNetwork/ModulePreservation.

## Author Summary

In network applications, one is often interested in studying whether modules are preserved across multiple networks. For example, to determine whether a pathway of genes is perturbed in a certain condition, one can study whether its connectivity pattern is no longer preserved. Non-preserved modules can either be biologically uninteresting (e.g., reflecting data outliers) or interesting (e.g., reflecting sex specific modules). An intuitive approach for studying module preservation is to cross-tabulate module membership. But this approach often cannot address questions about the preservation of connectivity patterns between nodes. Thus, cross-tabulation based approaches often fail to recognize that important aspects of a network module are preserved. Cross-tabulation methods make it difficult to argue that a module is *not* preserved. The weak statement (“the reference module does not overlap with any of the identified test set modules”) is less relevant in practice than the strong statement (“the module cannot be found in the test network irrespective of the parameter settings of the module detection procedure”). Module preservation statistics have important applications, e.g. we show that the wiring of apoptosis genes in a human cortical network differs from that in chimpanzees.

## Introduction

Network methods are frequently used in genomic and systems biologic studies, but also in general data mining applications, to describe the pairwise relationships of a large number of variables [1], [2]. For example, gene co-expression networks can be constructed on the basis of gene expression data [3]–[10]. In many network applications, one is interested in studying the properties of network modules and their change across conditions [11]–[16]. For example, [17]–[19] studied modules across multiple mouse tissues, [20] studied module preservation between human brain and blood tissue, and [21] studied module preservation between human and mouse brains.

This article describes several module preservation statistics for determining which properties of a network module are preserved in a second (test) network. The module preservation statistics allow one to quantify which aspects of within-module topology are preserved between a reference network and a test networks. For brevity, we will refer to these aspects as connectivity patterns, but we note that our statistics are not based on network motifs. We use the term “module” in a broad sense: a network module is a subset of nodes that forms a sub-network inside a larger network. Any subset of nodes inside a larger network can be considered a module. This subset may or may not correspond to a cluster of nodes.

Many cluster validation statistics proposed in the literature can be turned into module preservation statistics. In the following, we briefly review cluster validation statistics. Traditional cluster validation (or quality) statistics can be split into four broad categories: cross-tabulation, density, separability, and stability statistics [22]–[24]. Since cross-tabulation statistics compare cluster assignments in the reference and test clusterings, they require that a clustering procedure is also applied to the test data. On the other hand, density and density/separability statistics do not require a clustering in the test data set. These statistics typically evaluate clusters by how similar objects are within each cluster and/or how dis-similar objects are between different clusters [25]. Stability statistics typically study cluster stability when a controlled amount of artificial noise is added to the data. Although stability statistics also evaluate clusters, they are more relevant to comparing clustering procedures rather than quantifying cluster preservation and hence we do not consider them here.

While many cluster validation statistics are based on within- and/or between cluster variance, several recent articles used prediction error to evaluate the reproducibility (or validity) of clusters in gene expression data [24], [26], [27]. These papers argued that the use of a measure of test set clusters defined by a classifier made from the reference data is an appropriate approach to cluster validation when the aim is to identify reproducible clusters of genes or microarrays with similar expression profiles. For example, the in-group proportion (IGP), which is similar to the cluster cohesion statistic [28], is defined as the proportion of observations classified to a cluster whose nearest neighbor is also classified to the same cluster [24]. One can also calculate a significance level (p-value) for the IGP statistic. A comparison of the IGP statistic to alternative cluster quality statistics found that the IGP performs well [24]. Thus, we use the IGP statistic as benchmark statistic for assessing the use of module preservation statistics in case that modules are defined as clusters. Our simulation studies and applications show that one of our module preservation statistics is sometimes closely correlated with the IGP statistic if the modules are defined as clusters. But cluster validation statistics (such as the IGP) may not be appropriate when modules are not defined as clusters. **In general, assessing module preservation is a different task from assessing cluster preservation**. In our simulations, we demonstrate that module preservation statistics can detect aspects of module preservation that are missed by existing cluster validation statistics.

## Results

### Overview of module preservation statistics

Table 1 presents an overview of the module preservation statistics studied in this article. We distinguish between cross-tabulation based and network based preservation statistics. **Cross-tabulation based preservation statistics** require independent module detection in the test network and take the module assignments in both reference and test networks as input. Several cross-tabulation based statistics are described in the first section of Supplementary Text S1. While cross-tabulation approaches are intuitive, they have several disadvantages. To begin with, they are only applicable if the module assignment in the test data results from applying a module detection procedure to the test data. For example, a cross-tabulation based module preservation statistic would be meaningless when modules are defined as gene ontology categories since both reference and test networks contain the same sets of genes. But a non-trivial question is whether the network connections of a module (gene ontology category) in the reference network resemble those of the same module in the test network. To measure the resemblance of network connectivity, we propose several measures based on network statistics. Network terminology is reviewed in Table 2 and in Methods.

**Table 1:** Overview of module preservation statistics.

No.	Preservation Statistic			Network	Ref. netw. input			Test netw. input			Used in composite		
	Name	Eq.	Type		Lbl	Adj	*datX*	Lbl	Adj	*datX*	*Zsum.*	*medR.*	*Zsum.A*
1	coClustering	Supp.	Cross-tab	not used	yes	no	no	yes	no	no	no	no	no
2	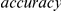	Supp.	Cross-tab	not used	yes	no	no	yes	no	no	no	no	no
3	−log(p-value)	Supp.	Cross-tab	not used	yes	no	no	yes	no	no	no	no	no
4	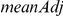	8	Density	general	yes	no	no	no	yes	no	no	no	yes
5	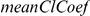	9	Density	general	yes	no	no	no	yes	no	no	no	no
6	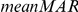	10	Density	general	yes	no	no	no	yes	no	no	no	no
7	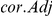	11	Connect.	general	yes	yes	no	no	yes	no	yes	yes	yes
8	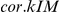	12	Connect.	general	yes	yes	no	no	yes	no	yes	yes	yes
9	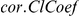	13	Connect.	general	yes	yes	no	no	yes	no	no	no	no
10	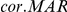	14	Connect.	general	yes	yes	no	no	yes	no	no	no	no
11	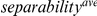	27	Separab.	general	yes	yes	no	no	yes	no	no	no	no
12	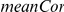	19	Den.+Con.	cor	yes	no	yes	no	no	yes	yes	yes	no
13	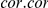	20	Connect.	cor	yes	no	yes	no	no	yes	yes	yes	no
14	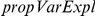	21	Density	cor	yes	no	yes	no	no	yes	yes	yes	no
15	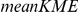	22	Den.+Con.	cor	yes	no	yes	no	no	yes	yes	yes	no
16	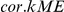	23	Connect.	cor	yes	no	yes	no	no	yes	yes	yes	no
17	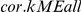	24	Connect.	cor	yes	no	yes	no	no	yes	no	no	no
18	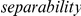	28	Separab.	cor	yes	no	yes	no	no	yes	no	no	no
19	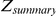	1	Compos.	cor	yes	yes	yes	no	yes	yes			
20	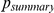		Compos.	cor	yes	yes	yes	no	yes	yes			
21	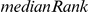	34	Compos.	cor	yes	yes	yes	no	yes	yes			
22	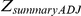	35	Compos.	general	yes	yes	no	no	yes	no			

The columns report the names, types, and input of individual preservation statistics (Lbl, module label; Adj, general network adjacency; 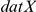, numeric data from which a correlation network is constructed). The last 3 columns indicate which of the individual statistics are used in the composite summary statistics 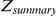, 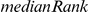, and 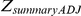, respectively. The definition of cross-tabulation based statistics can be found in Supplementary Text S1.

**Table 2:** Glossary of network terminology.

Term	Definition
(Undirected) Network	Generally speaking, an undirected network consists of nodes (for example, gene expression profiles), and connection strengths between pairs of nodes. The connection strengths can be either categorical (connected vs. unconnected), or continuous between 0 (no connection) and 1 (strongest connection).
Adjacency matrix	The connection strengths in an undirected network can be represented by the *adjacency matrix*, a symmetric matrix whose entries lie between 0 and 1. The element 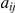 is the connection strength between nodes 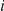 and 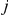 . As a convention, the diagonal elements are set to 1, 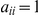 .
Correlation network	This type of network is built from numerical data 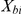 representing the value of variable 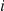 in observation 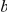 . The adjacency (connection strength) 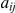 between nodes 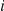 and 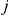 is calculated from the correlation of the corresponding node profiles 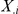 and 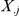 . In our applications, we use Equation 15 or 16 to calculate the adjacency from correlations.
Gene co-expression network	In gene co-expression networks, the nodes represent genes (or probesets of a microarray) measured across a given set of microarray samples, and the connections represent the strength of co-expression. Various measures of co-expression can be used, for example Pearson or robust correlation (in which case the co-expression network is also a correlation network), information-theoretic methods such as mutual information, and other measures of co-expression similarity.
Sub-network	A subnetwork of a network can be any collection (subset) of nodes from the network, together with the adjacencies (connection strengths) between the nodes. Thus, a subnetwork of a network also forms a (smaller) network on its own.
Module	A network module is a subset of nodes that forms a sub-network inside a larger network. Any subset of nodes inside a larger network gives rise to a module. This subset may or may not correspond to a cluster of nodes.
Cluster	A cluster of nodes within a network is usually defined as a group of nodes that are strongly connected. Many definitions and algorithms for finding clusters in data have been proposed in the literature.
Network density	The mean adjacency (connection strength) among all nodes in the network.
Connectivity	For each node, the connectivity (also known as degree) is defined as the sum of connection strengths with the other network nodes: 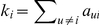 . In co-expression networks, the connectivity measures how correlated a gene is with all other network genes.
Intramodular connectivity 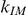	Intramodular connectivity measures how connected, or co-expressed, a given node is with respect to the nodes of a particular module. Thus, intramodular connectivity is also the connectivity in the subnetwork defined by the module. The intramodular connectivity may be interpreted as a measure of module membership.
Module eigennode 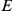	The module eigennode 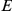 is defined as the first principal component of a given module. For a co-expression module, the module eigengene can be considered a representative of the gene expression profiles in a module.
Eigennode-based connectivity 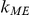 , also known as module membership ( 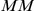 )	For the i-th vector 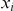 (e.g. gene expression profile), 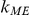 equals the correlation of 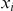 with the module eigennode. For example in a co-expression network application, 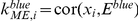 measures how correlated gene 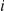 is with the eigengene of the blue module. Thus, 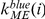 measures the membership of the 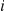 -th gene with respect to the blue module. If 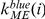 is close to 0, the 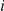 -th gene is not part of the blue module. The sign of module membership encodes whether the gene has a positive or a negative relationship with the blue module eigengene. The module membership measure can be defined for all input genes (irrespective of their original module membership). It turns out that 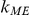 is often highly related with the intramodular connectivity 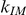 [29].

Even when modules are defined using a module detection procedure, cross-tabulation based approaches face potential pitfalls. A module found in the reference data set will be deemed non-reproducible in the test data set if no matching module can be identified by the module detection approach in the test data set. Such non-preservation may be called the **weak non-preservation:** *“the module cannot be found using the current parameter settings of the module detection procedure”*. On the other hand, one is often interested in **strong non-preservation:** *“the module cannot be found irrespective of the parameter settings of the module detection procedure”*. Strong non-preservation is difficult to establish using cross-tabulation approaches that rely on module assignment in the test data set. A second disadvantage of a cross-tabulation based approach is that it requires that for each reference module one finds a matching test module. This may be difficult when a reference module overlaps with several test modules or when the overlaps are small. A third disadvantage is that cross-tabulating module membership between two networks may miss that the fact that the patterns of connectivity between module nodes are highly preserved between the two networks.

**Network based statistics** do not require the module assignment in the test network but require the user to input network adjacency matrices (described in Methods). We distinguish the following 3 types of network based module preservation statistics: 1) density based, 2) separability based, and 3) connectivity based preservation statistics. **Density based** preservation statistics can be used to determine whether module nodes remain highly connected in the test network. **Separability based** statistics can be used to determine whether network modules remain distinct (separated) from one another in the test network. While numerous measures proposed in the literature combine aspects of density and separability, we keep them separate and provide evidence that density based approaches can be more useful than separability based approaches in determining whether a module is preserved. **Connectivity based** preservation statistics can be used to determine whether the connectivity pattern between nodes in the reference network is similar to that in the test network. As detailed in Methods, several module preservation statistics are similar to previously proposed cluster quality and preservation statistics, while others (e.g. connectivity based statistics) are novel.

Table 1 reports the **required input** for each preservation statistic. Since each preservation statistic is used to evaluate the preservation of modules defined in a reference network, it is clear that each statistic requires the module assignment from the reference data. But the statistics differ with regard to the module assignment in the test data. Only cross-tabulation based statistics require a module assignment in the test data. Network based preservation statistics do not require a test set module assignment. Instead, they require the test set network adjacency matrix (for a general network) or the test data set 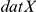 of numeric variables (for a correlation network).

We distinguish network statistics by the underlying network. Some preservation statistics are defined for a general network (defined by an adjacency matrix) while others are only defined for a correlation network (constructed on the basis of pairwise correlations between numeric variables). Our applications show that the correlation structure facilitates the definition of particularly powerful module preservation statistics. Preservation statistics 4–11 (Table 1) can be used for general networks while statistics 12–19 assume correlation networks. Network density and module separability statistics only need the test set adjacency matrix while the connectivity preservation statistics also require the adjacency matrix in the reference data.

It is often not clear whether an observed value of a preservation statistic is higher than expected by chance. As detailed in Methods, we attach a significance level (permutation test p-value) to observed preservation statistics, by using a permutation test procedure which randomly permutes the module assignment in the test data. Based on the permutation test we are also able to estimate the mean and variance of the preservation statistic under the null hypothesis of no relationship between the module assignments in reference and test data. By standardizing each observed preservation with regard to the mean and variance, we define a 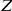 statistic for each preservation statistic. Under certain assumptions, each 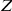 statistic (approximately) follows the standard normal distribution if the module is not preserved. The higher the value of a Z statistic, the stronger the evidence that the observed value of the preservation statistic is significantly higher than expected by chance.

#### Composite preservation statistics and threshold values

Because preservation statistics measure different aspects of module preservation, their results may not always agree. We find it useful to aggregate different module preservation statistics into composite preservation statistics. Composite preservation statistics also facilitate a fast evaluation of many modules in multiple networks. We define several composite statistics.

For correlation networks based on quantitative variables, the 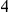 density preservation statistics are summarized by 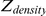 (Equation 30), the 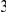 connectivity based statistics are summarized by 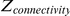 (Equation 31), and all individual 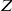 statistics are summarized by 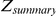 defined as follows
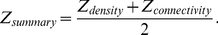
(1)As detailed in the Methods, our simulations suggest the following thresholds for 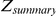: if 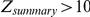 there is strong evidence that the module is preserved; if 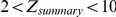 there is weak to moderate evidence of preservation; if 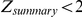, there is no evidence that the module preserved. For general networks defined by an adjacency matrix, we find it expedient to summarize the preservation statistics into a summary statistic denoted 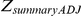 (Equation 35).

Since biologists are often more familiar with p-values as opposed to Z statistics, our R implementation in function modulePreservation also calculates empirical p-values. Analogous to the case of the Z statistics, the p-values of individual preservation statistic are summarized into a descriptive measure called 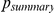. The smaller 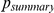, the stronger the evidence that the module is preserved. In practice, we observe an almost perfect inverse relationship (Spearman correlation 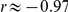) between 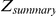 and 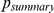.

The Z statistics and permutation test p-values often depend on the module size (i.e. the number of nodes in a module). This fact reflects the intuition that it is more significant to observe that the connectivity patterns among hundreds of nodes are preserved than to observe the same among say only 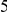 nodes. Having said this, there will be many situations when the dependence on module size is not desirable, e.g., when preservation statistics of modules of different sizes are to be compared. In this case, we recommend to either focus on the observed values of the individual statistics or alternatively to summarize them using the composite module preservation statistic 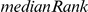 (Equation 34). The 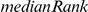 is useful for comparing relative preservation among multiple modules: a module with lower median rank tends to exhibit stronger observed preservation statistics than a module with a higher median rank. Since 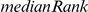 is based on the observed preservation statistics (as opposed to Z statistics or p-values) we find that it is much less dependent on module size.

### Application 1: Preservation of the cholesterol biosynthesis module between mouse tissues

Several studies have explored how co-expression modules change between mouse tissues [19] and/or sexes [18]. Here we re-analyze gene expression data from the liver, adipose, muscle, and brain tissues of an F2 mouse intercross described in [13], [17]. The expression data contain measurements of 17104 genes across the following numbers of microarray samples: 137 (female (F) adipose), 146 (male (M) adipose), 146 (F liver), 145 (M liver), 125 (F muscle), 115 (M muscle), 148 (F brain), and 141 (M brain).

We consider a single module defined by the genes of the gene ontology (GO) term “Cholesterol biosynthetic process” (CBP, GO id GO:0006695 and its GO offspring). Of the 28 genes in the CBP, 24 could be found among our 17104 genes. Cholesterol is synthesized in liver and we used the female liver network as the reference network module. As test networks we considered the CBP co-expression networks in other tissue/sex combinations.

**Figure 1:**
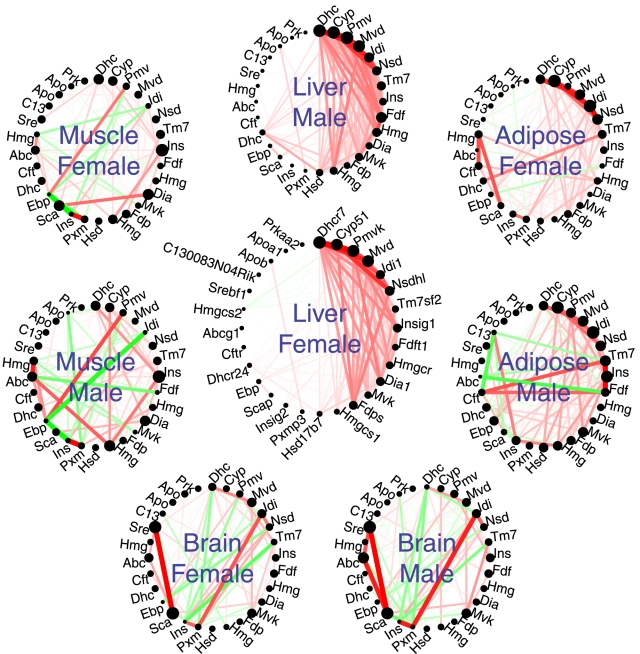
Network plot of the module of cholesterol biosynthesis genes in different mouse tissues. The module is defined as a signed weighted correlation network among genes from the GO category Cholesterol Biosynthetic Process. Module preservation statistics allow one to quantify similarities between the depicted networks. The figure depicts the connectivity patterns (correlation network adjacencies) between cholesterol biosynthesis genes in 4 different mouse tissues from male and female mice of an F2 mouse cross. The thickness of the line reflects the absolute correlation. The line is colored in red if the correlation is positive and green if it is negative. The size of each black circle indicates the connectivity of the corresponding gene; hubs (i.e., highly connected) genes are represented by larger circles. Visual inspection suggests that the male and female liver networks are rather similar and show some resemblance to those of the adipose tissue. Module preservation statistics can be used to measure the similarity of connectivity patterns between pairs of networks.

Each circle plot in Figure 1 visualizes the connection strengths (adjacencies) between CBP genes in different mouse tissue/sex combination. The color and width of the lines between pairs of genes reflect the correlations of their gene expression profiles across a set of microarray samples. Before delving into a quantitative analysis, we invite the reader to visually compare the patterns of connections. Clearly, the male and female liver networks look very similar. Because of the ordering of the nodes, the hubs are concentrated on the upper right section of the circle and the right side of the network is more dense. The adipose tissues also show this pattern, albeit much more weakly. On the other hand, the figures for the brain and muscle tissues do not show these patterns. Thus, the figure suggests that the CBP module is more strongly preserved between liver and adipose tissues than between liver and brain or muscle.

**Figure 2:**
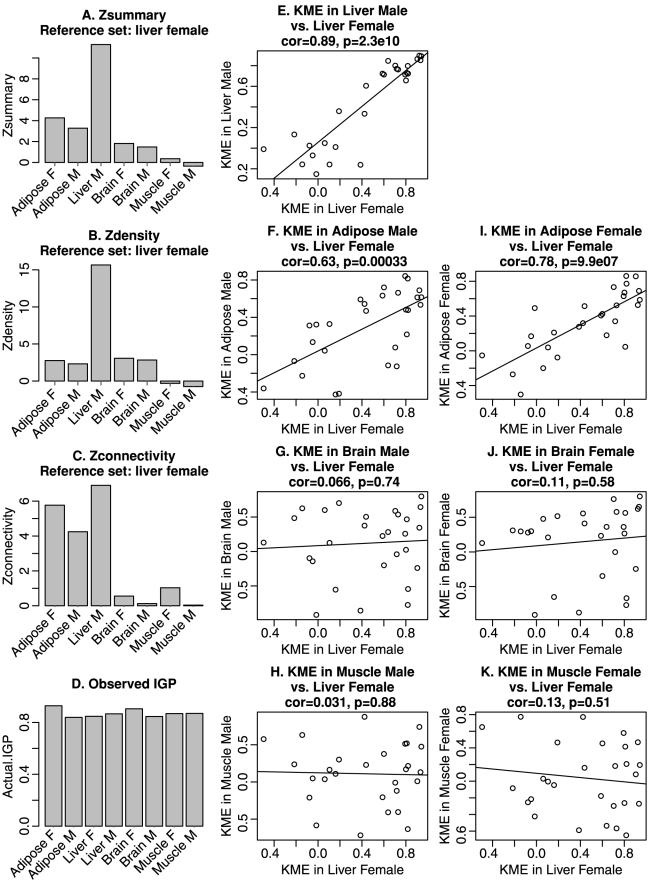
Preservation of GO term cholesterol biosynthetic process across mouse tissues. Quantitative evaluation of the similarities among the networks depicted in Figure 1. As reference module, we define a correlation network among the genes of the GO term “Cholesterol biosynthetic process” (CBP) in the female mouse liver network. Panels A–C show summary preservation statistics in other tissue and sex combinations. Panel A shows the composite preservation statistic 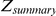. The CBP module in the female liver network is highly preserved in the male liver network (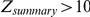) and moderately preserved in adipose networks. There is no evidence of preservation in brain or muscle tissue networks. Panels B and C show the density and connectivity statistics, respectively. Panel D shows the results of the in group proportion analysis [24]. According to the IGP analysis, the CBP module is equally preserved in all networks. E–K show the scatter plots of 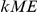 in one test data set (indicated in the title) vs. the liver female reference set. Each point corresponds to a gene; Pearson correlations and the corresponding p-values are displayed in the title of each scatter plot. The eigengene-based connectivity 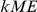 is strongly preserved between adipose and liver tissues; it is not preserved between female liver and the muscle and brain tissues.

We now turn to a quantitative assessment of this example. We start out by noting that a **cross-tabulation based approach** of module preservation is meaningless in this example since the module is a GO category whose genes can trivially be found in each network. However, it is a very meaningful exercise to measure the similarity of the connectivity patterns of the module genes across networks. To provide a quantitative assessment of the connectivity preservation, it is useful to adapt network concepts (also known as network statistics or indices) that are reviewed in Methods. Figure 2 provides a quantitative assessment of the preservation of the connectivity patterns of the cholesterol biosynthesis module between the female liver network and networks from other sex/tissue combinations. Figure 2A presents the composite summary statistic (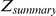, Equation 1) in each test network. Overall, we find strong evidence of preservation (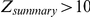, Equation 1) in the male liver network but no evidence (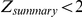) of preservation in the female brain and muscle networks. We find that the connectivity of the female liver CBP is most strongly preserved in the male liver network. It is also weakly preserved in adipose tissue but we find no evidence for its preservation in muscle and brain tissues. The summary preservation statistic 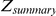 measures both aspects of density and of connectivity preservation. We now evaluate which of these aspects are preserved. Figure 2B shows that the module shows strong evidence of density preservation (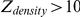) (Equation 30) in the male liver network but negligible density preservation in the other networks. Interestingly, Figure 2C shows that the module has moderate connectivity preservation 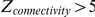 (Equation 31) in the adipose networks.

The 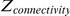 measure summarizes the statistical significance of 3 connectivity based preservation statistics. Two of our connectivity measures evaluate whether highly connected intramodular hub nodes in the reference network remain hub nodes in the test network. Preservation of intramodular connectivity reflects the preservation of hub gene status between the reference and test network. One measure of intramodular connectivity is the module eigengene-based connectivity measures 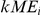 (Equation 17), which is also known as the module membership measure of gene 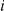 [13], [29], [30]. Genes with high values of 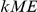 are highly correlated with the summary profile of the module (module eigengene defined as the first principal component, see the fifth section in Supplementary Text S1). A high correlation of 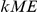 between reference and test network can be visualized using a scatter plot and quantified using the correlation coefficient 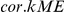. For example, Figure 2I shows that 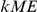 in the female liver module is highly correlated with that of the male liver network (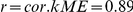, 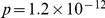). Further, the scatter plots in Figure 2 show that the 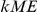 measures between liver and adipose networks show strong correlation (preservation): 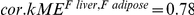 (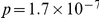), 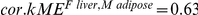 (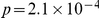), 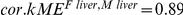 (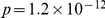), while the correlation between 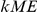 in female liver and the brain and muscle data sets are not significant. This example demonstrates that connectivity preservation measures can uncover a link between CBP in liver and adipose tissues that is missed by density preservation statistics.

We briefly compare the performance of our network based statistics with those from the IGP method [24]. The R implementation of the IGP statistic requires that at least 2 modules are being evaluated. To get it to work for this application that involves only a single module, we defined a second module by randomly sampling half of the genes from the rest of the entire network. Figure 2D shows high, nearly constant values of the IGP statistic across networks, which indicates that the CBP module is present in all data sets. Note that the IGP statistic does not allow us to argue that the CBP module in the female liver network is more similar to the CBP module in the male liver than in other networks. This reflects the fact that the IGP statistic, which is a cluster validation statistic, does not measure connectivity preservation.

### Application 2: Preservation of human brain modules in chimpanzee brains

**Figure 3:**
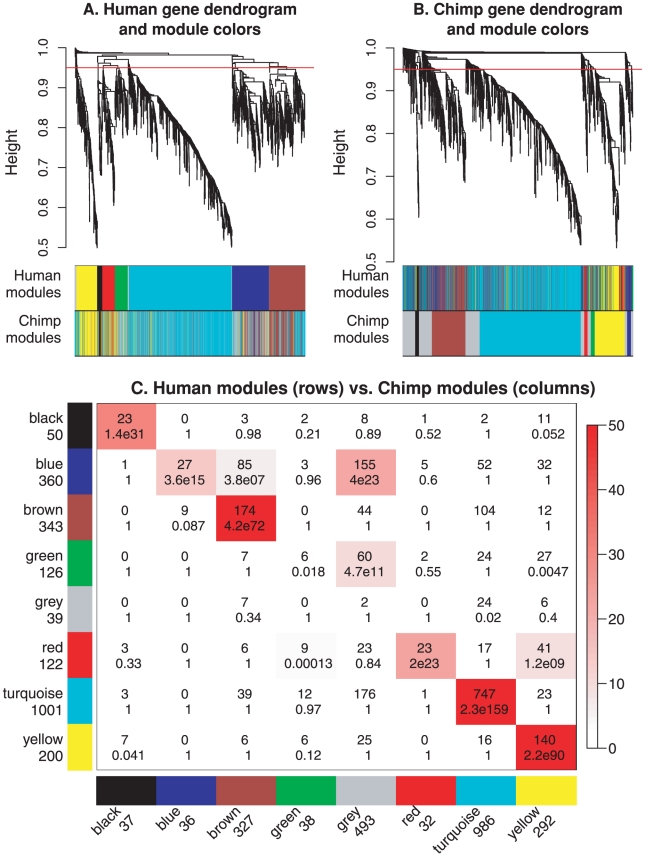
Cross-tabulation based comparison of modules (defined as clusters) in human and chimpanzee brain networks. A. Hierarchical clustering tree (dendrogram) of genes based on human brain co-expression network. Each “leaf” (short vertical line) corresponds to one gene. The color rows below the dendrogram indicate module membership in the human modules (defined by cutting branches of this dendrogram at the red line) and in the chimpanzee network (defined by branch cutting the dendrogram in panel B.) The color rows show that most human and chimpanzee modules overlap (for example, the turquoise module). B. Hierarchical clustering tree of genes based on the chimpanzee co-expression network. The color rows below the dendrogram indicate module membership in the human modules (defined by cutting branches of dendrogram in panel A.) and in the chimpanzee network (defined by branch cutting the dendrogram in this panel.) C. Cross-tabulation of human modules (rows) and chimpanzee modules (columns). Each row and column is labeled by the corresponding module color and the total number of genes in the module. In the table, numbers give counts of genes in the intersection of the corresponding row and column module. The table is color-coded by 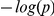, the Fisher exact test p value, according to the color legend on the right. Note that the human yellow network is highly preserved while the human blue network is only weakly preserved in the chimpanzee network.

Here we study the preservation of co-expression between human and chimpanzee brain gene expression data. The data set consists of 18 human brain and 18 chimpanzee brain microarray samples [31]. The samples were taken from 6 regions in the brain; each region is represented by 3 microarray samples. Since we used the same weighted gene co-expression network construction and module identification settings as in the original publication, our human modules are identical to those in [32]. Because of the relatively small sample size only few relatively large modules could be detected in the human data. The resulting modules were labeled by colors: turquoise, blue, brown, yellow, green, black, red (see Figure 3A). Oldham *et al* (2006) determined the biological meaning of the modules by examining over-expression of module genes in individual brain regions. For example, heat maps of module expression profiles revealed that the turquoise module contains genes highly expressed in cerebellum, the yellow module contains genes highly expressed in caudate nucleus, the red module contains genes highly expressed in anterior cingulate cortex (ACC) and caudate nucleus, and the black module contains mainly genes expressed in white matter. The blue, brown and green modules contained genes highly expressed in cortex, which is why we refer to these modules as cortical modules. Visual inspection of the module color band below the dendrograms in Figures 3A and 3B suggests that most modules show fairly strong preservation. Oldham *et al* argued that modules corresponding to evolutionarily older brain regions (turquoise, yellow, red, black) show stronger preservation than the blue and green cortical modules [32]. Here we re-analyze these data using module preservation statistics.

The most common **cross-tabulation approach** starts with a contingency table that reports the number of genes that fall into modules of the human network (corresponding to rows) versus modules of the chimpanzee network (corresponding to columns). The contingency table in Figure 3C shows that there is high agreement between the human and chimpanzee module assignments. The human modules black, brown, red, turquoise, and yellow have well-defined chimpanzee counterparts (labeled by the corresponding colors). On the other hand, the human green cortical module appears not to be preserved in chimpanzee since most of its genes are classified as unassigned (grey color) in the chimpanzee network. Further, the human blue cortical module (360 genes) appears to split into several parts in the chimpanzee network: 27 genes are part of the chimpanzee blue module, 85 genes are part of the chimpanzee brown module, 52 fall in the chimpanzee turquoise module, 155 genes are grey in the chimpanzee network, etc. To arrive at a more quantitative measure of preservation, one may quantify the module overlap or use Fisher's exact test to attach a significance level (p-value) to each module overlap (as detailed in the first section of Supplementary Text S1). The contingency table in Figure 3C shows that every human module has significant overlap with a chimpanzee module. However, even if the resulting p-value of preservation were not significant, it would be difficult to argue that a module is truly a human-specific module since an alternative module detection strategy in chimpanzee may arrive at a module with more significant overlap. In order to quantify the preservation of human modules in chimpanzee samples more objectively, one needs to consider statistics that do not rely on a particular module assignment in the chimpanzee data.

**Figure 4:**
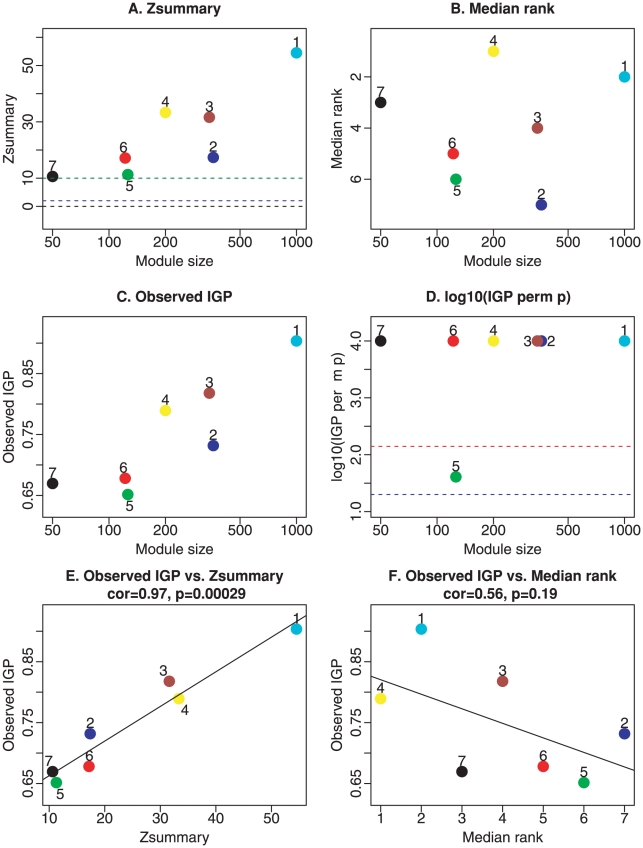
Composite preservation statistics of human modules in chimpanzee samples. A. The summary statistic 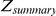 (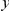-axis), Equation 1, as a function of the module size. Each point represents a module, labeled by color and a secondary numeric label (1 = turquoise, 2 = blue, 3 = brown, 4 = yellow, 5 = green, 6 = red, 7 = black). The dashed blue and green lines indicate the thresholds 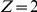 and 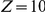, respectively. B. The composite statistic 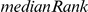 (y-axis), Equation 34, as a function of the module size. Each point represents a module, labeled by color and a secondary numeric label as in panel A. Low numbers on the 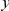 axis indicate a high preservation. C. Observed IGP statistic (Kapp and Tibshirani, 2007) versus module size. D. P-value of the IGP statistic versus module size. E. and F. show scatter plots between the observed IGP statistic and 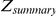 and 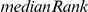, respectively. In this example, where modules are defined as clusters, the IGP statistic has a high positive correlation (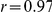) with 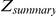 and a moderately large negative correlation (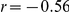) with 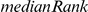. The negative correlation is expected since low median ranks indicate high preservation.

We now turn to approaches for measuring module preservation that do not require that module detection has been carried out in the test data set. Figures 4A,B show composite module preservation statistics of human modules in chimpanzee samples. The overall significance of the observed preservation statistics can be assessed using 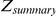 (Equation 1) that combines multiple preservation 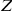 statistics into a single overall measure of preservation, Figure 4A. Note that 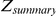 shows a strong dependence on module size, which reflects the fact that observing module preservation of a large module is statistically more significant than observing the same for a small module. However, here we want to consider all modules on an equal footing irrespective of module size. Therefore, we focus on the composite statistic 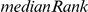 which shows no dependence on module size (Figure 4B). The median rank is useful for comparing relative preservation among modules: a module with lower median rank tends to exhibit stronger observed preservation statistics than a module with a higher median rank. Figure 4B shows that the median ranks of the human brain modules. The median rank of the yellow module is 1, while the median ranks of the blue module is 6, indicating that the yellow module is more strongly preserved than the blue module. Our quantitative results show that modules expressed mainly in evolutionarily more conserved brain areas such as cerebellum (turquoise) and caudate nucleus (yellow and partly red) are more strongly preserved than modules expressed primarily in the cortex that is very different between humans and chimpanzees (green and blue modules). Thus the module preservation results of 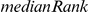, corroborate Oldham's original finding regarding the relative lack of preservation of cortical modules.

Since the modules of this application are defined as clusters, it makes sense to evaluate their preservation using cluster validation statistics. Figure 4C shows that the IGP statistic implemented in the R package clusterRepro [24] also shows a strong dependence on module size in this application. The IGP values of all modules are relatively high. However, the permutation p-values (panels C and D) identify the green module as less preserved than the other modules (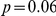, Bonferroni corrected p-value 0.43). Figures 4E,F show scatter plots between the observed IGP statistic and 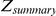 and 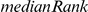, respectively. In this example, where modules are defined as clusters, the IGP statistic has a high positive correlation (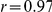) with 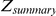 and a moderately large negative correlation (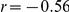) with 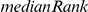. The negative correlation is expected since low median ranks indicate high preservation.

While composite statistics summarize the results, it is advisable to understand which properties of a module are preserved (or not preserved). For example, **module density based** statistics allow us to determine whether the genes of a module (defined in the reference network) remain densely connected in the test network. As an illustration, we will compare the module preservation statistics for the human yellow module whose genes are primarily expressed in caudate nucleus (an evolutionarily old brain area), and the human blue module whose genes are expressed mostly in the cortex which underwent large evolutionary changes between humans and chimpanzees. In chimpanzees, the mean adjacency of the genes comprising the human yellow module is significantly higher than expected by chance, with a high permutation statistic 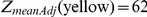, 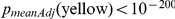. But the corresponding permutation 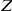 statistic for the human blue module is only weakly significant, 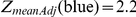, 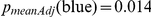 (see Supplementary Text S2 and Supplementary Table S1). Thus, the mean adjacency permutation statistic suggests that the blue module is less preserved than the yellow module.

**Figure 5:**
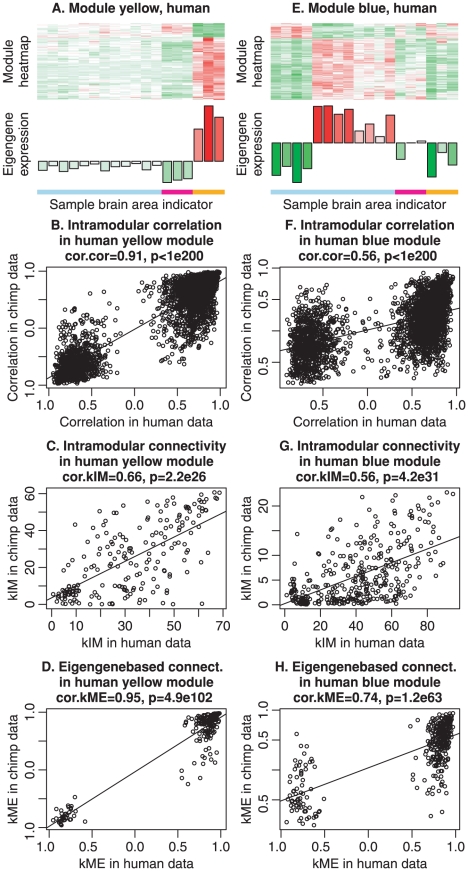
Connectivity-based statistics for evaluating the preservation of the human yellow and blue modules in the chimpanzee network. A. Heatmaps and eigengene plots for visualizing the gene expression profiles of the yellow module genes (rows) across human brain microarray samples (columns). In the heat map, green indicates under-expression, red over-expression, and white mean expression. The module eigengene expression depicted underneath the heat map shows how the eigengene expression (y-axis) changes across the samples (x-axis) which correspond to the columns of the heat map. The eigengene can be interpreted as a weighted average gene expression profile. The color bar below the eigengene indicates the region from which the sample was taken: light blue color indicates cortical samples, magenta indicates cerebellum samples, and orange indicates caudate nucleus samples. Scatter plots B.–D. show that the connectivity patterns of the yellow module genes tends to be highly preserved between the two species. B. Scatter plot of gene-gene correlations in chimpanzee samples (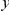-axis) vs. human samples (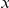-axis) within the human yellow module. Each point corresponds to a gene-gene pair. The scatter plot exhibits a significant correlation (cor.cor and p-value displayed in the title), indicating that the correlation pattern among the genes is preserved between the human and chimpanzee data. C. Scatter plot of intramodular connectivities, Equation 7, of genes in the human yellow module in chimpanzee samples (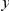-axis) vs. human samples (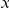-axis). Each point corresponds to one gene. The scatter plot exhibits a significant correlation (cor.kIM and p-value displayed in the title), indicating that the hub gene status in the human yellow module is preserved in the chimpanzee samples. D. Scatter plot of eigengene-based connectivities, Equation 17, of genes in chimpanzee samples (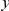-axis) vs. human samples (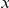-axis). Each point corresponds to one gene. The scatter plot exhibits a significant correlation (cor.kME and p-value displayed in the title), indicating that fuzzy module membership in the human yellow module is preserved in the chimpanzee samples. Scatter plots E.–H. show that the human blue module is less preserved in the chimpanzee network. Note that the correlations in scatter plots F.–H. are lower than the corresponding correlations in the yellow module plots B.–D., indicating weaker preservation of the human blue module in the chimpanzee samples. Overall, these results agree with those from the cross-tabulation based analysis reported in Figure 3.

For co-expression modules, one can define an alternative density measure based on the module eigengene (Figures 5A and E). The higher the proportion of variance explained by the module eigengene (defined in the fifth section in Supplementary Text S1) in the test set data, the tighter is the module in the test set. The human yellow module exhibits a high proportion of variance explained, 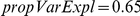, and the corresponding permutation 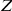 statistic is 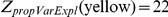, 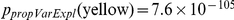. In contrast, for the human blue module we find 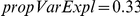 and the corresponding permutation 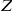 statistic is 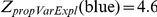, 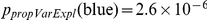. The permutation statistics again suggest that the yellow module is more preserved than the blue module.

Although density based approaches are intuitive, they may fail to detect another form of module preservation, namely the **preservation of connectivity patterns** among module genes. For example, network module connectivity preservation can mean that, within a given module 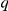, a pair of genes with a high connection strength (adjacency) in the reference network also exhibits a high connection strength in the test network. This property can be quantified by correlating the pairwise adjacencies or correlations between reference and test networks. For the genes in the human yellow module, the scatter plot in Figure 5B shows pairwise correlations in the human network (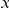-axis) versus the corresponding correlations in the chimpanzee network (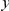-axis). The correlation between pairwise correlations (denoted by 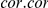) equals 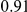 and is highly significant, 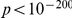. The analogous correlation for the blue module, Figure 5F is lower, 0.56, but still highly significant, 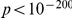, in part because of the higher number of genes in the blue module.

A related but distinct connectivity preservation statistic quantifies whether intramodular hub genes in the reference network remain intramodular hub genes in the test network. Intramodular hub genes are genes that exhibit strong connections to other genes within their module. This property can be quantified by the *intramodular connectivity* 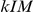 (Equation 7): hub genes are genes with high 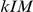. Intramodular hub genes often play a central role in the module [5], [33]–[35]. Preservation of intramodular connectivity reflects the preservation of hub gene status between the reference and test network. For example, the intramodular connectivity of the human yellow module is preserved between the human and chimpanzee samples, 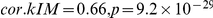 (Figure 5C). In contrast, the human blue (cortical) module exhibits a lower correlation (preservation) 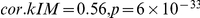 (Figure 5G). The 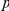 value is more significant because of the higher number of genes in the blue module.

Another intramodular connectivity measure is 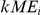, which turns out to be highly related with 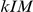 [29]. Figure 5D shows that 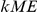 for the human yellow module is highly preserved in the chimpanzee network (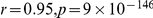). The corresponding correlation in the human blue module is lower, 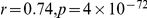 (Figure 5H). In summary, the observed preservation statistics show that the human yellow module (related to the caudate nucleus) is more strongly preserved in the chimpanzee samples than the human blue module (related to the cortex).

### Application 3: Preservation of KEGG pathways between human and chimpanzee brains

To further illustrate that modules do not have to be clusters, we now describe an application where modules correspond to KEGG pathways. KEGG (Kyoto Encyclopedia of Genes and Genomes) is a knowledge base for systematic analysis of gene functions, linking genomic information with higher order functional information [36]. KEGG also provides graphical representations of cellular processes, such as signal transduction, metabolism, and membrane transport. To illustrate the use of the module preservation approach, we studied the preservation of selected KEGG pathway networks across human and chimpanzee brain correlation networks. While pathways in the KEGG database typically describe networks of proteins, our analysis describes the correlation patterns between mRNA expression levels of the corresponding genes. As before, we define a weighted correlation network adjacency matrix between the genes (described in the third section of Supplementary Text S1 and [5]). For the sake of brevity, we focused the analysis on the following 8 signaling pathways: Hedgehog signaling pathway (12 genes in our data sets), apoptosis (24 genes in our data sets), TGF-beta signaling pathway (26 genes), Phosphatidylinositol signaling system (39 genes), Wnt signaling pathway (55 genes), Endocytosis (59 genes), Calcium signaling pathway (78 genes), and MAPK signaling pathway (93 genes). All of these pathways have been shown to play critical roles in normal brain development and function [37]–[41]. We provide a brief description of the functions of these pathways in Methods; more detailed description can be found in the KEGG database and in numerous textbooks.

**Figure 6:**
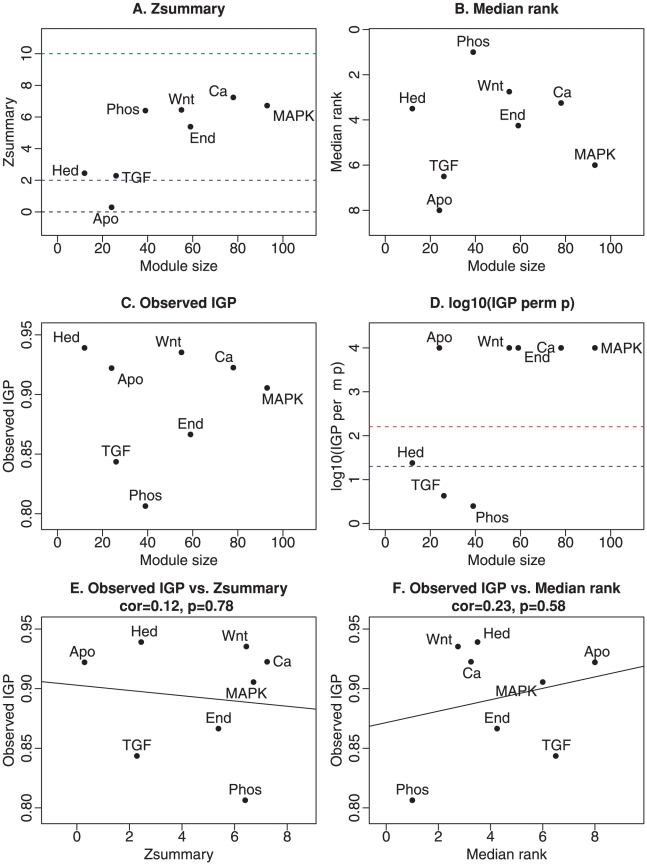
Composite preservation statistics for KEGG pathways between human and chimp brain networks. Here we present the composite statistics 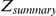 (panel A) and 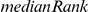 (panel B), and the IGP statistic (panels C and D). Panels E. and F. show scatter plots between the observed IGP statistic and 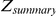 and 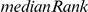, respectively. Here we find no significant relationship between the IGP statistic and the composite module preservation statistic. Since KEGG modules do not correspond to clusters, it is not clear whether cluster preservation statistics are useful in this example.

**Figure 7:**
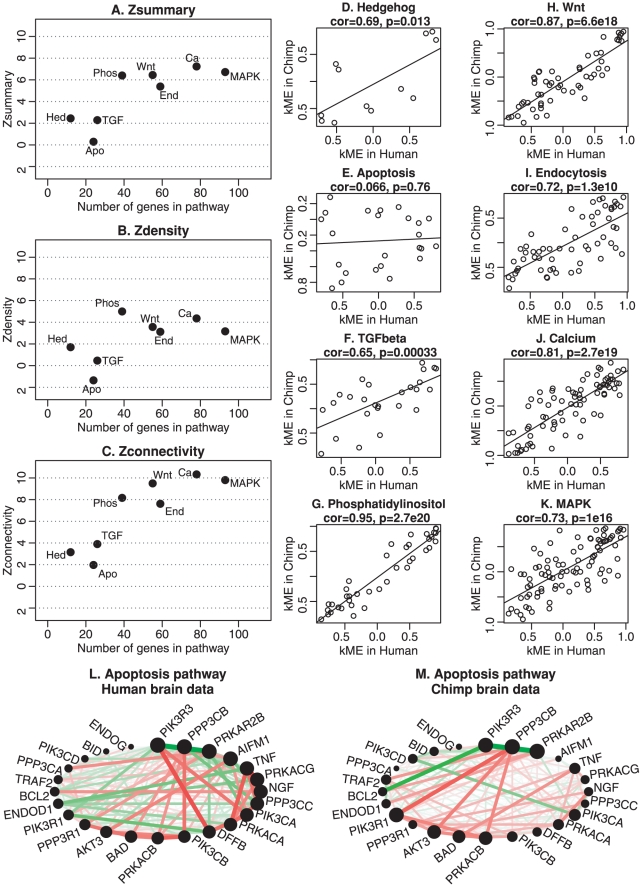
Detailed preservation analysis of KEGG pathways between human and chimp brain networks. The first column presents summary preservation 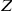 statistics (y-axis) for selected KEGG pathways (interpreted as modules) versus the number of genes in the pathway (x-axis). Panel A shows 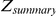 (Equation 1), panel B shows the density summary statistic 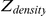 (Equation 30), and panel C shows the connectivity summary statistic 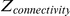 (Equation 31). Pathway names are shortened for readability. Panel A shows that MAPK, Calcium, Endocytosis, Wnt, and Phosphatidylinositol show strong evidence of preservation (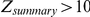) while the apoptosis module is not preserved. Panel C shows that this preservation signal mainly reflects connectivity preservation 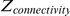 (Equation 31) while panel B reveals that most modules have weak to moderate density preservation (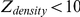) (Equation 30). Note that the apoptosis pathway shows no evidence of preservations. Panels D–H display scatter plots of eigengene-based connectivities in the chimpanzee data (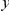-axis) vs. in the human data (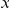-axis). Each point represents a gene in the pathway. Higher correlation means that the internal co-expression structure of the pathway is more strongly preserved. The apoptosis pathway has the lowest 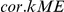 statistic, while the Phosphatidylinositol pathway has the highest. The circle plots in panels L and M show connection strengths among apoptosis genes in humans and chimpanzees, respectively.

Figures 6A,B show the composite preservation statistics 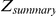 and 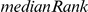. Both statistics indicate that the apoptosis module is the least preserved module. To visualize the lack of preservation, consider the circle plots of apoptosis genes in Figures 7 L, M that show pronounced differences in the connectivity patterns among apoptosis genes. While we caution the reader that additional data are needed to replicate these differences, prior literature points to an evolutionary difference for apoptosis genes. For example, a scan for positively selected genes in the genomes of humans and chimpanzees found that a large number of genes involved in apoptosis show strong evidence for positive selection [42]. Further, it has been hypothesized that natural selection for increased cognitive ability in humans led to a reduced level of neuron apoptosis in the human brain [43].

Figure 6A shows that 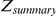 exhibits some dependence on module size. Since we want to compare module preservation irrespective of module size, we focus on the results for the 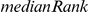 statistic (Figure 6B). A reviewer of this article hypothesized that gene sets (modules) known to be controlled by coexpression (such as Wnt, TGF-beta, SRF, interferon, lineage specific differentiation markers, and NF kappa B) would show stronger evidence of preservation than gene sets without a priori reason for suspecting such control (calcium signaling, MAPK, apoptosis, chemotaxis, endocytosis). Interestingly, the results for the 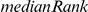 statistic largely validate this hypothesis. Specifically, the 4 most highly preserved pathways according to 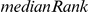 are Wnt (controlled by coexpression), calcium (not controlled), Hedgehog (controlled), and Phosphatidylinositol (not commented upon). The 4 least preserved pathways are apoptosis (not controlled), TGF-beta (controlled), MAPK (not controlled), endocytosis (not controlled).

Since KEGG pathways are not defined via a clustering procedure it is not clear whether cluster preservation statistics are appropriate for analyzing this example. But to afford a comparison, we also report the findings for the IGP statistic [24]. Figures 6C and D show that IGP identifies Phosphatidilinositol and TGF-beta as the least preserved modules while apoptosis genes are highly preserved. We find no significant relationship between the IGP statistic and our module preservation statistics 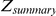 and 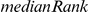 (Figures 6E and F). This example highlights that module preservation statistics can lead to very different results from cluster preservation statistics.

To understand which aspects of the pathways are preserved, one can study the preservation of density statistics (Figure 7B) and of connectivity statistics (Figure 7C). According to 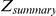, the coexpresssion network formed by apoptosis genes is not preserved. It neither shows evidence of connectivity preservation (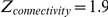) nor evidence of density preservation (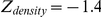, 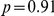). The Hedgehog pathway also shows no evidence of density preservation (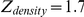, 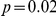) but it shows weak evidence of connectivity preservation (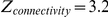, 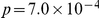). The relatively low preservation Z statistics of the Hedgehog pathway may reflect a higher variability due to a small module size (it contains only 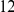 genes while the other pathways contain at least 22 genes). To explore this further, we studied the observed preservation statistics, which are less susceptible to network size effects than the corresponding 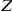 statistics. The scatter plots in Figure 7D–H show the correlations 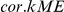 between eigengene based connectivity measures 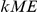 between the two species. For the Hedgehog pathway, we find that 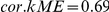 (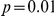) which turns out to be higher than that of the TGF-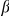 pathway.

The lack of preservation of the apoptosis pathway cannot be explained in terms of low module size. Figure 7E shows that it has the lowest observed 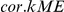 statistic, 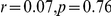.

This application outlines how module preservation statistics can be used to study the preservation of KEGG pathway networks. The analysis presented here is but a first step towards characterizing molecular pathway preservation between human and chimpanzee brains, and should be extended through more detailed analyses with additional data sets in the future. A limitation of our microarray data is that they measured expression levels in heterogeneous mixtures of cells. KEGG and GO (gene ontology) pathways all essentially describe interactions that take place within cells. So when data have been generated from a heterogeneous mixture of different cell types, it is possible that these relationships are somewhat obscured. It is not obvious that all of the elements of a KEGG pathway should be co-expressed, particularly since the pathways describe protein-protein interactions.

### Application 4: Preservation of modules between male and female cortex co-expression networks

We briefly describe an application that quantifies module preservation between male and female cortical samples. The details are described in Supplementary Text S3 and in Supplementary Table S2. We used microarray data from a recent publication [30] to construct consensus modules [44] in male samples from 2 different data sets. We then studied the preservation of these modules in the corresponding female samples. Cross-tabulation measures indicate that for 3 of the male modules there are no corresponding modules in the female data. However, our network preservation statistics show that in fact the three modules show moderate to strong evidence of preservation. Thus, in this application the network preservation statistics protect one from making erroneous claims of significant sex differences.

### Application 5: Preservation of female mouse liver co-expression modules in male mice

In Supplementary Text S4, we re-analyze the mouse liver samples of the F2 mouse intercross [13], [17] to study whether “female” co-expression modules (i.e., modules found in a network based on female mice) are preserved in the corresponding male network. This application demonstrates that module preservation statistics allow us to identify invalid, non-reproducible modules due to array outliers. A comprehensive table of module preservation statistics for this application is presented in Supplementary Table S3.

### Application 6: Preservation of consensus modules

Our preservation statistics allow one to evaluate whether a given module is preserved in another network. A related but distinct data analysis task is to construct modules that are present in several networks. By construction, a consensus module can be detected in each of the underlying networks. A challenge of many real data applications is that it is difficult to obtain independent information (a “gold standard”) that allows one to argue that a module is truly preserved. To address this challenge, we use the consensus network application where by construction, modules are known to be preserved. This allows us to determine the range of values of preservation statistics when modules are known to be preserved. In Supplementary Text S5 and Supplementary Table S4, we report three empirical studies of consensus modules [44] which are constructed in such a way that genes within consensus modules are highly co-expressed in all given input microarray data sets. The consensus module application provides further empirical evidence that module preservation statistics and the recommended threshold values provide sufficient statistical power to implicate preserved modules.

### Relationships among module preservation statistics

**Figure 8:**
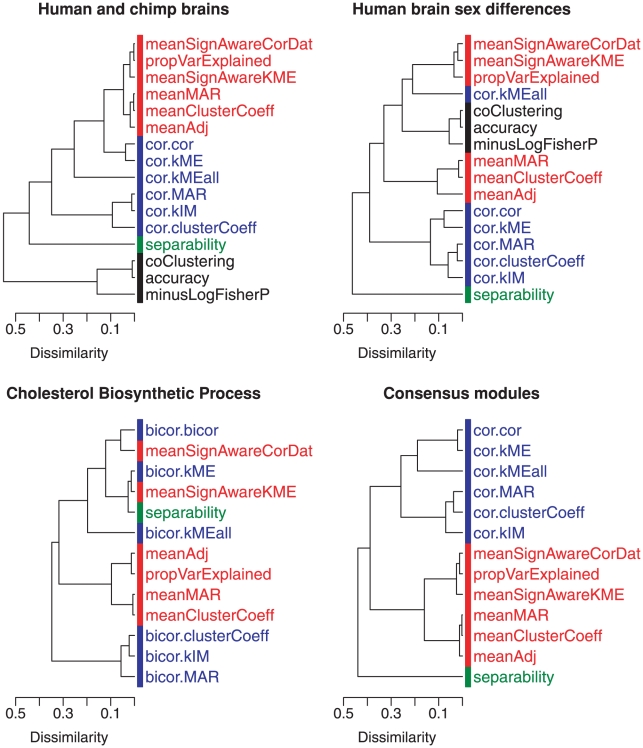
Relationships between module preservation statistics based on applications. The (average linkage) hierarchical cluster trees visualize the correlations between the preservation statistics. The preservation statistics are colored according to their type: density statistics are colored in red, connectivity preservation statistics are colored in blue, separability is colored in green, and cross-tabulation statistics are colored in black. Note that statistics of the same type tend to cluster together. A derivation of some of these relationships is presented in Supplementary Text S1.

In Table 1, we categorize the statistics according to which aspects of module preservation they measure. For example, we present several seemingly different versions of density and connectivity based preservation statistics. But for correlation network modules, close relationships exist between them as illustrated in Figure 8. The hierarchical clustering trees in Figure 8 show the correlations between the observed preservation statistics in our real data applications. As input of hierarchical clustering, we used a dissimilarity between the observed preservation statistics, which was defined as one minus the correlation across all studied reference and test data sets. Overall we observe that statistics within one category tend to cluster together. We also observe that separability appears to be weakly related to the density and connectivity preservation statistics. Cross-tabulation statistics correlate strongly with density and connectivity statistics in the study of human and chimpanzee brain data, but the correlation is weak in the study of sex differences in human brain data.

We derive relationships between module preservations statistics in the sixth section of Supplementary Text S1. In particular, the geometric interpretation of correlation networks [29], [45] can be used to describe situations when close relationship exist among the density based preservation statistics (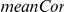, 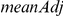, 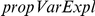, 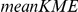), among the connectivity based preservation statistics (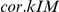, 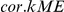, 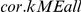, 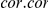), and between the separability statistics (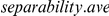, 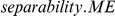). These relationships justify aggregating the module preservation statistics into composite preservation statistics such as 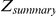 (Equation 1) and 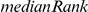 (Equation 34).

### Simulation studies and comparisons

**Figure 9:**
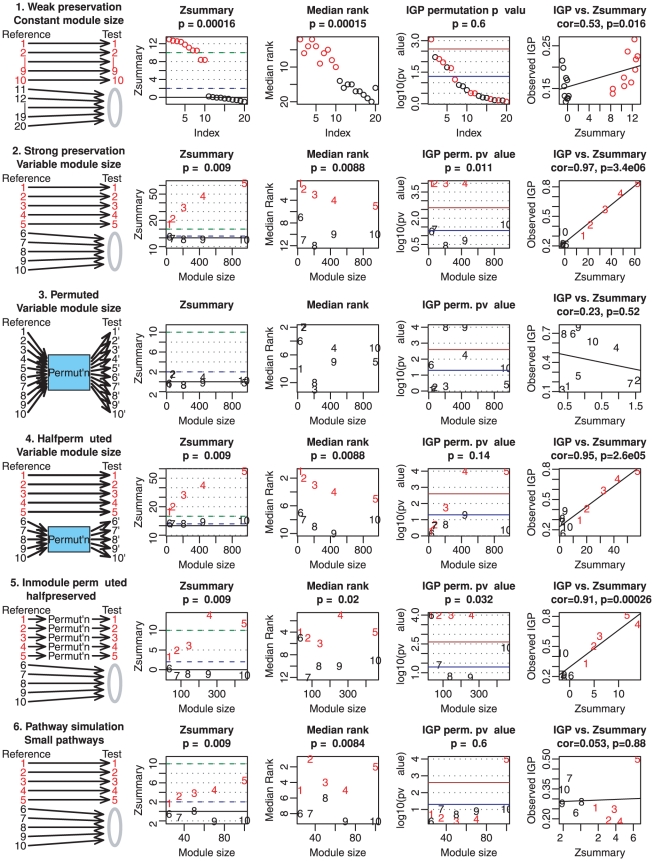
Design and main results of simulation studies of module preservation. The first column outlines 6 (out of 7) simulation scenarios. Results for the seventh simulation scenario can be found in Supplementary Text S6. Preserved and non-preserved modules are marked in red and black, respectively. The grey module (labeled 0) represents genes whose profiles are simulated to be independent (that is, without any correlation structure). The second and third columns report values of composite statistics 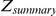 and 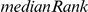, respectively, as a function of module size. The blue and green horizontal lines show the thresholds of 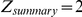 and 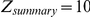, respectively. Each figure title reports the Kruskal-Wallis test p-value for testing whether the preservation statistics differ between preserved and non-preserved modules. Note that the proposed thresholds (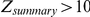 for preserved and 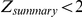 for non-preserved modules) work quite well. The fourth column shows the permutation p-values of IGP obtained by the R package clusterRepro. The blue and brown lines show p-value thresholds of 0.05 and its Bonferroni correction, respectively. The IGP permutation p-value is less successful than 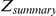 at distinguishing preserved from non-preserved modules. The fifth and last column shows scatter plots of observed IGP vs. 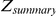. We observe that IGP and 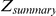 tend to be highly correlated when modules correspond to clusters with varying extents of preservation.

To illustrate the utility and performance of the proposed methods, we consider 7 different simulation scenarios that were designed to reflect various correlation network applications. An overview of these simulations can be found in Figure 9. A more detailed description of the simulation scenarios is provided below.

Table 3 shows the performance grades of module preservation statistics in the different simulation scenarios. The highest grade of 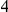 indicates excellent performance. We find that the proposed composite statistics 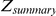 (mean grade 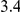) and 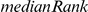 (mean grade 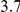) perform very well in distinguishing preserved from non-preserved modules. In contrast, cross-tabulation based statistics only obtain a mean grade of 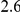. Since several simulation scenarios test the ability to detect connectivity preservation (as opposed to density preservation), it is no surprise that on average cluster validation statistics do not perform well in these simulations. For example, the IGP cluster validation statistic (Table 4) obtains a mean grade of 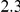 across the scenarios. But the IGP performs very well (grade 4) when studying the preservation of strongly preserved clusters (scenario 2).

**Table 3:** Overview of the performance of various module preservation statistics in our simulation studies.

No.	Statistic	Type	Network	Simulation scenario							Mean
				1	2	3	4	5	6	7	
1	coClustering	Cross-tab	not used	1	4	3	1	4	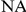 *	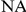 *	2.6
2	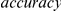	Cross-tab	not used	1	4	4	1	3	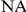 *	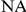 *	2.6
3	−log(p-value)	Cross-tab	not used	1	4	3	1	4	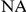 *	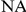 *	2.6
4	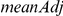	Density	general	4	4	3	3	4	1	1	2.9
6	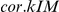	Connectiv.	general	2	3	4	3	3	3	4	3.1
12	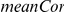	Den.+Con.	cor	4	4	4	4	1	1	2	2.9
13	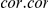	Connectiv.	cor	3	4	4	4	1	3	4	3.3
14	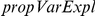	Density	cor	3	4	3	2	4	1	1	2.6
15	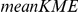	Den.+Con.	cor	4	3	4	4	3	1	2	3
16	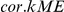	Connectiv.	cor	2	4	3	3	1	3	4	2.9
17	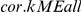	Connectiv.	cor	4	2	4	3	3	1	1	2.6
18	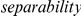	Separabil.	cor	1	1	3	3	1	1	1	1.6
19	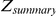	Composite	cor	3	4	4	3	3	3	4	3.4
21	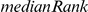	Composite	cor	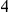 §	4	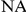 §	4	2	4	4	3.7

*Value not available since the pathway membership is determined beforehand and the same in both data sets.

**§:** Since no thresholds can be defined for the statistic 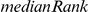, the top grade of 4 was assigned if the 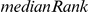 perfectly distinguished preserved from non-preserved modules, i.e. if the 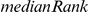 of each preserved module was smaller than that of all non-preserved modules. For simulated scenario 3 (Perm.), a grade is not available (NA) since none of the modules were simulated to be preserved.

Evaluating preservation statistics in different simulation scenarios. Best and worst performance correspond to grade 4 and 1, respectively. The columns report the name, type, and input of individual preservation statistics, and their performance our 7 simulation studies summarized in Figure 9: 1. weak module preservation, 2. strong module preservation, 3. permuted module membership, 4. half-permuted module membership, 5. in-module permutation, 6. small pathway, and 7. large pathway. We graded each statistic as follows: Statistics that accurately distinguish preserved and non-preserved modules within the thresholds of *Z*≲2 for non-preserved modules and *Z*≳10 for preserved modules get grade 4; statistics that accurately distinguish preserved and non-preserved modules but whose values may lie on the wrong side of the thresholds are graded 3; statistics that distinguish preserved and non-preserved modules with high accuracy (allowing 20%, that is 1 of 5, mis-classifications) are graded 2, and statistics that perform worse are graded 1. The grading of 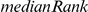 is explained in 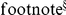. We only report selected statistics defined for correlation networks.

**Table 4:** Comparison of summary preservation statistics to in group proportion.

No.	Statistic	Type	Network	Simulation scenario							Mean
				1	2	3	4	5	6	7	
19	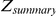	Composite	cor	3	4	4	3	3	3	4	3.4
21	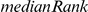	Composite	cor	4	4	NA	4	2	4	4	3.7
	IGP	Dens+Sep	General	1	4	3	3	3	1	1	2.3
	IGP perm. p	Dens+Sep	General	1	4	3	2	2	1	1	2.0

Comparison of summary preservation statistics to in group proportion (IGP) described in [24]. Column headings and performance grading are the same as in Table 3.

Table 3 also shows the performance of individual preservation statistics. Note that density based preservation statistics perform well in scenarios 1 through 5 but fail in scenarios 6 and 7. On the other hand, all connectivity based statistics perform well in scenarios 6 and 7. The relatively poor performance of 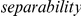 is one of the reasons why we did not include it into our composite statistics.

In the following, we describe the different simulation scenarios in more detail.

1. In the **weak preservation simulation** scenario, we simulate a total of 20 module in the reference data. Each of the 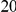 reference modules contains 200 nodes. But only 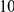 of the 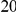 modules are simulated to be preserved in the test network. We call it the weak preservation simulation since the intramodular correlations of preserved modules are relatively low. The intramodular correlations of non-preserved modules are expected to be zero. Note that the summary statistic 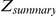 successfully distinguishes preserved from non-preserved modules (second column of Figure 9), with 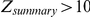 for 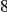 of the 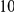 preserved modules. Similarly, the 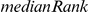 statistic distinguishes preserved from non-preserved modules (third column of Figure 9). In comparison, the IGP permutation p-value (fourth column of Figure 9) is less successful: only 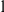 of the 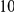 preserved modules pass the Bonferroni-corrected threshold; of the 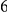 modules that pass the 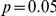 threshold, 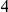 are preserved and 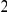 are non-preserved. In this simulation we observe a moderate relationship between the observed IGP and 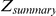, with Pearson correlation 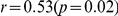.
2. In the **half-preserved simulation** scenario, we simulate 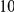 modules of varying sizes (between 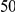 and 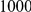 nodes), labeled 1–10. Modules 1–5 are preserved in the test set, while modules 6–10 are not preserved. All 5 preserved modules have 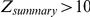, and all non-preserved modules have 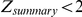. Likewise, 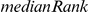 separates preserved and non-preserved modules. Permutation p-values of IGP are also successful with respect to the Bonferroni-corrected threshold. In this simulation we observe a strong correlation between IGP and 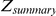: 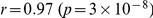.
3. In the **permuted simulation** scenario, none of the 10 modules are preserved. Specifically, we simulate 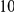 modules of varying sizes in the reference set and 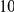 modules of the same sizes in the test set but there is no relationship between the modules: the module membership is randomly permuted between the networks. The low value of the summary preservation statistic 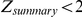 accurately reflects that none of the modules are preserved. In contrast, the IGP permutation p-value for 2 of the 10 modules is lower than the Bonferroni threshold 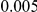. In this simulation the correlation between IGP and 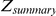 is not significant.
4. In the **half-permuted simulation** scenario, we simulate 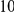 modules labeled 1–10 in the reference set. Modules 1–5 are preserved in the test set, while modules 6–10 are not. The test set contains modules 6′–10′ of the same sizes as modules 6–10, but their module membership is randomly permuted with respect to the modules 6–10. The summary preservation statistic 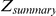 is quite accurate: all 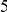 preserved modules have 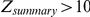 and 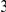 non-preserved modules have 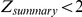. The observed values of the IGP statistic are highly correlated (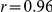, 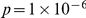) with 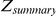 but the IGP permutation p-values do not work well: 2 preserved modules have an IGP p-value above 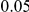.
5. In the **intramodular permuted** scenario, we simulate modules whose density is preserved but whose intramodular connectivity is not preserved. Specifically, we simulate a total of 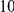 modules labeled 1–10 in the reference set. The density of modules 1–5 is preserved in the test set but the node labels inside each module are permuted, which entails that their intramodular connectivity patterns is no longer preserved in the test network. For modules 6–10 neither the density nor the connectivity is preserved. Both composite statistics 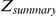 and 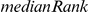 work well though not as good as in the previous studies. Both composite statistics successfully detect the density preservation. IGP performs quite well: it misclassifies only one non-preserved module as preserved. In this simulation we observe a strong correlation between IGP and 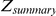: 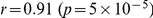.
6. In the **pathway simulations** scenario, we simulate 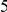 (preserved) modules whose connectivity patterns are preserved but whose density is not. Further, we simulate 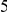 modules for which neither connectivity nor density are preserved. In the following description, we refer to the modules from scenario 4 as clusters to distinguish them from the non-cluster modules studied here. The 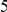 preserved (non-preserved) modules of the pathway scenario are created by randomly selecting nodes from the preserved (non-preserved) clusters in scenario 4. Thus, the preserved modules contain nodes from multiple preserved clusters of scenario 4. Since the pairwise correlations between and within the preserved clusters (of scenario 4) are preserved, the intramodular connectivity patterns of the resulting pathway modules are preserved in the test network. But since nodes from different clusters may have low correlations, the density of the pathway modules tends to be low. The two pathway simulations differ by the module sizes: in the small scenario, modules range from 25 to 100 nodes; in the large scenario, modules range from 100 to 500 nodes. Because module membership is trivially preserved between reference and test networks, cross-tabulation statistics are not applicable. The composite statistics 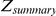 and 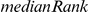 distinguish preserved from non-preserved modules (Figure 9 since they also measure aspects of connectivity preservation. By considering individual preservation statistics, we find that all connectivity preservation statistics successfully distinguish preserved from non-preserved modules. As expected, density based statistics and the IGP statistic fail to detect the preservation of the connectivity patterns of the 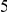 preserved modules (Figure 9) but these statistics correctly indicate that the density is not preserved. Detailed results are provided in Supplementary Text S6.

Additional descriptions of the simulations can be found Supplementary Text S6 and in Supplementary Table S5. As caveat, we mention that we only considered 7 scenarios that aim to emulate selected situations encountered in co-expression networks. The performance of these preservation statistics may change in other scenarios. A comprehensive evaluation in other scenarios is needed but lies beyond our scope. R software tutorials describing the results of our simulation studies can be found on our web page and will allow the reader to compare different methods using our simulated data.

### Software implementation

Preservation statistics described in this article have been implemented in the freely available statistical language and environment R. A complete evaluation of observed preservation statistics and their permutation 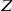 statistics is implemented in function modulePreservation, which is included in the updated WGCNA package originally described in [46]. For each user-defined reference network both preservation and quality statistics are calculated considering each of the remaining networks as test network. Our tutorials illustrate the use of the modulePreservation function on real and simulated data. All data, code and tutorials can be can be downloaded from http://www.genetics.ucla.edu/labs/horvath/CoexpressionNetwork/ModulePreservation.

## Discussion

This article describes powerful module preservation statistics that capture different aspects of module preservation. The network based preservation statistics only assume that each module forms a sub-network of the original network. Thus, we define a module as a subset of nodes with their corresponding adjacencies. In particular, our connectivity preservation statistics (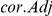, 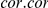, 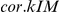, and 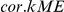) do not assume that modules are defined as clusters. While we have used connectivity based statistics in biologic applications (e.g., modular preservation in human and mouse networks [20], [21]), this article provides the first methodological description and evaluation of these and other module preservation statistics. We also demonstrate that it is advantageous to aggregate multiple preservation statistics into composite statistics 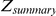 and 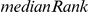. While we propose module preservation statistics for general networks (e.g., 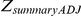), all of our applications involve gene co-expression networks.

For a special class of networks, called approximately factorizable networks, one can derive simple relationships between network concepts [29], [45]. Analogously, we characterize correlation modules where simple relationships exist between i) density-based preservation statistics, ii) connectivity based preservation statistics, and iii) separability based preservation statistics (see the sixth section of Supplementary Text S1). We also briefly describe relationships between preservation statistics in general networks.

Table 3 shows the performance grades of module preservation statistics in different simulation scenarios. We find that composite statistics 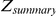 and 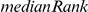 perform very well in distinguishing preserved from non-preserved modules. While the dependence of 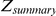 on the module size is often attractive, our applications show situations when it is unattractive. In this case, we recommend to use the composite statistic 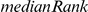, which has an added bonus: its computation is much faster than that of 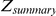 since it does not involve a permutation test procedure. Our applications provide evidence that the 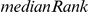 statistic can lead to biologically meaningful preservation rankings among modules.

### Uses of module preservation statistics

Our applications provide a glimpse of the types of research questions that can be addressed with the module preservation statistics. In general, methods for quantifying module preservation have several uses. First and foremost they can be used to determine which properties of a network module are preserved in another network. Thus, module preservation statistics are a valuable tool for validation as well as differential network analysis. Second, they can be used to define a global measure of module structure preservation by averaging the preservation statistic across multiple modules or by determining the proportion of modules that are preserved. A third use of module preservation statistics is to define measures of module quality (or robustness), which may inform the module definition. For example, to measure how robustly a module is defined in a given correlation network, one can use resampling techniques to create reference and test sets from the original data and evaluate module preservation across the resulting networks. Thus, any module preservation statistic naturally gives rise to a module quality statistic by applying it to repeated random splits (interpreted as reference and test set) of the data. By averaging the module preservation statistic across multiple random splits of the original data one arrives at a module quality statistic.

We briefly point out situations when alternative procedures may be more appropriate. To identify modules that are present in multiple data sets it can be preferable to consider all data sets simultaneously in a consensus module detection procedure. For example, the consensus module approach described in application 6 results in modules that are present in multiple networks by construction. To identify individual genes that diverge between two data sets, one can use standard discriminative analysis techniques. For example, differentially expressed genes can be found with differential expression analysis and differentially co-expressed genes can be found using differential co-expression analysis [17].

### Comparison to cluster preservation statistics

While cluster analysis and network analysis are different approaches for studying high-dimensional data, there are some commonalities. For example, it is straightforward to turn a network adjacency matrix (which is a similarity measure) into a dissimilarity measure which can be used as input of a clustering procedure (e.g., hierarchical clustering or partitioning around medoids) [25]. If a module is defined using a clustering procedure, one can use cluster preservation statistics as module preservation statistics. Conversely, our adjacency based module preservation statistics give rise to cluster preservation statistics since a dissimilarity measure (used for the cluster definition) can also be transformed into a network adjacency matrix. In some of our applications where modules are defined as clusters, we find that 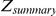 is highly correlated with the IGP cluster validation statistic [24] across modules. In our simulations, we observe that IGP and 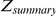 tend to be highly correlated when modules correspond to clusters with varying extents of preservation. This illustrates that 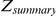 leads to sensible results in the special case when modules are defined as clusters. When modules are not defined via a clustering procedure (e.g. in our KEGG pathway application), we find pronounced differences between 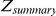 and the IGP statistic.

The proposed composite preservation statistics 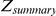 and 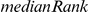 outperform (or tie with) the IGP statistic in all 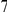 simulation scenarios (see Table 4). More comprehensive comparisons involving additional simulation scenarios and other cluster preservation statistics are needed but lie beyond our scope.

### Module quality measures

Although not the focus of this work, we mention that a major application of density-based statistics is to measure module *quality* in the reference data (for example, to compare various module detection procedures). Module quality measures can be defined using density-based and separability-based module preservation measures: the density and separability of a module in the *reference* network measures its homogeneity and separateness, respectively. In contrast, connectivity based measures (which contrast the reference adjacency matrix with the test network adjacency matrix) are not directly related to module quality measures (unless a data splitting approach is used in the reference data). Module quality measures based on density and separability measures can be used to confirm that the reference modules are well defined. A section in Supplementary Text S1 describes module quality measures that are implemented in the R function modulePreservation.

### Limitations

The proposed preservation statistics have several limitations including the following. First, our statistics only apply to undirected networks. Generalization of our statistics to directed networks is possible but outside of our scope.

A second limitation concerns statistics of connectivity preservation that are based on correlating network adjacencies, intramodular connectivities, etc, between the reference and the test networks. Because Pearson correlation is sensitive to outliers, it may be advantageous to use an outlier-resistant correlation measure, e.g., the Spearman correlation or the biweight midcorrelation [47], [48] implemented in the WGCNA package [46]. Robust correlation options have been implemented in the R function modulePreservation.

A third limitation is that a high value of a preservation statistic does not necessarily imply that the module could be found by a *de novo* module detection analysis in the test data set. For example, if a module is defined using cluster analysis, then the resulting test set modules may not have significant overlap with the original reference module in a cross-tabulation table. As explained before, this potential limitation is a small price to pay for making a module preservation analysis independent from the vagaries of module detection.

A fourth limitation is that it is difficult to pick thresholds for preservation statistics. To address this issue, we use permutation tests to adjust preservation statistics for random chance by defining Z statistics (Equation 29). The R function modulePreservation also calculates empirical p-values for the preservation statistics. A potential disadvantage of permutation test based preservation statistics (compared to observed statistics and 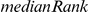) is that they typically depend on module sizes. The choice of thresholds is discussed in the Methods section.

A fifth limitation is computational speed when it comes to calculating permutation test based statistics (e.g. 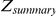). When only 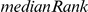 and observed preservation statistics are of interest, we recommend to avoid the computationally intensive permutation test procedure by setting nPermutations = 0 in the modulePreservation function.

A sixth limitation is that the different preservation statistics may disagree with regard to the preservation of a given module. While certain aspects of a module may be preserved, others may not be. In our simulation studies, we present scenarios where connectivity statistics show high preservation but density measures do not and vice versa. Since both types of preservation statistics will be of interest in practice, our R function modulePreservation outputs all preservation statistics. Although we aggregate several preservation statistics into composite statistics, we recommend to consider all of the underlying preservation statistics to determine which aspects of a module are preserved.

While we describe situations when cross-tabulation based preservation statistics are not applicable, we should point out that cross-tabulation statistics also have the following advantages. First, they are often intuitive. Second, they can be applied when no network structure is present. Third, they work well when module assignments are strongly preserved and the modules remain separate in the test network. In the first section of Supplementary Text S1, we describe cross-tabulation based module preservation statistics which we have found to be useful.

### Discussion of the functional significance of co-expression relationships

We note that the interpretation of gene co-expression relationships depends heavily on biological context. For example, in a dataset consisting of samples from multiple tissue types, co-expression modules (that is, modules defined by co-expression similarity) will often distinguish genes that are expressed in tissue-specific patterns (e.g., [32], [49]). In a dataset consisting of samples from a single tissue type, co-expression modules may distinguish sets of genes that are preferentially expressed in distinct cell types that comprise that tissue (e.g., [30]). In a dataset consisting of samples from a homogeneous cellular population, co-expression modules may correspond more directly to sets of genes that work in tandem to perform various intracellular functions. In many cases, co-expression modules may not present immediate functional interpretations. However, previous work has shown that many co-expression modules are conserved across phylogeny [4], [21], [32], [50], enriched with protein-protein interactions [7], [21], [30], and enriched with specific functional categories of genes, including ribosomal, mitochondrial, synaptic, immune, hypoxic, mitotic, and many others [7], [21], [30], [33].

Although elucidating the functional significance of identified co-expression modules requires substantial effort from biologists and bioinformaticians, the importance of co-expression modules lies not only in their functional interpretation, but also in their reproducibility. Because transcriptome organization in a given biological system is highly reproducible [30], co-expression modules provide a natural framework for comparisons between species, tissues, and pathophysiological states. This framework can reduce dimensionality by approximately three orders of magnitude (e.g., moving from say 40,000 transcripts to 40 modules) [29], [33], while simultaneously placing identified gene expression differences within specific cellular and functional contexts (inasmuch as the cellular and functional contexts of the modules are understood). The co-expression modules themselves are simply summaries of interdependencies that are already present in the data. Preservation statistics can be used to address an important question in co-expression module based analyses: how to show whether the modules are robust and reproducible across data sets.

### Conclusions

Given the above-mentioned limitations, it is reassuring that the proposed module preservation statistics perform well in 6 real data applications and in 7 simulation scenarios. Although it would be convenient to have a single statistic and a corresponding threshold value for deciding whether a module is preserved, this simplistic view fails to realize that module preservation should be judged according to multiple criteria (e.g., density preservation, connectivity preservation, etc). Individual preservation statistics provide a more nuanced and detailed view of module preservation. Before deciding on module preservation, the data analyst should decide which aspects of a module preservation are of interest.

## Methods

### Cross-tabulation based preservation statistics

Due to space limitations, we have moved our description of cross-tabulation based preservation statistics to the first section of Supplementary Text S1. We briefly mention related measures reported in the literature. Our co-clustering statistic (in the first section of Supplementary Text S1) is similar to the cluster robustness measure [23], [51] and the accuracy based measures are conceptually related to a cluster discrepancy measure proposed in [23]. Cluster validation measures and approaches are reviewed in [52]. Many cross-tabulation based methods have been proposed to compare two clusterings (module assignments), e.g., the Rand index [53] or prediction based statistics [26], [27].

### Review of network adjacency matrix and network concepts

Our methods are applicable to weighted or unweighted networks that are specified by an *adjacency matrix* 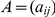, an 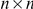 matrix with entries in 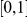. The component 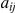 encodes the network connection strength between nodes 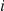 and 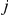. In an unweighted network, the nodes 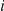, 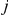 can be either connected (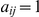) or disconnected (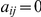). In a weighted network, the adjacency 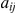 takes on a value in 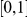 that encodes the connection strength between the nodes. Networks do not have to be defined with regard to correlations. Instead, they may reflect protein binding information, participation in molecular pathways, etc. In the following, we assume that we are dealing with an undirected network encoded by a symmetric adjacency matrix: 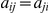. But several of our module preservation statistics can easily be adapted to the case of directed network represented by a non-symmetric adjacency matrix.

To simplify notation, we introduce the function 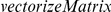 that takes a symmetric 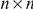 matrix 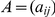 and turns it into a vector of non-redundant components,
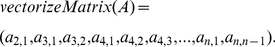
(2)We assume that the diagonal of the matrix 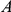 is fixed (for example, if 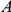 is an adjacency matrix, the diagonal is defined to be 1), so we leave the diagonal elements out. Thus, the vector 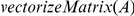 contains 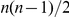 components.

A network represented by its adjacency matrix can be characterized by a number of network concepts (also known as network indices) [29], [45]. The *network density* is the mean adjacency,
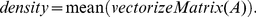
(3)Higher density means more (or more strongly) interconnected nodes.

The *connectivity* (also known as degree) 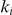 of node 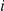 is defined as
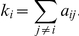
The connectivity of node 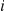 measures its connection strength with other nodes. The higher 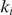 the more centrally located is the node in the network.

The Maximum Adjacency Ratio (MAR) [29] of node 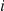 is defined as
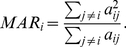
(4)The 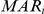 is only useful for distinguishing the connectivity patterns of nodes in a weighted network since it is constant (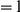) in unweighted networks.

The clustering coefficient [54] of node 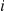 is defined as
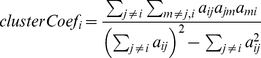
(5)While the clustering coefficient was originally defined for unweighted networks, Equation 5 can be used to extend its definition to weighted networks [5]: one can easily show that 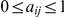 implies 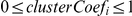.

### Intramodular network concepts

Many network analyses define modules, that is subsets of nodes that form a sub-network in the original network. Modules are labeled by integer labels 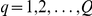, and sometimes by color labels. Color labels can be convenient for visualizing modules in network plots. For module 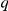 with 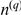 nodes, the 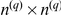 dimensional adjacency matrix between the module nodes is denoted by 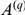. Denote by 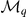 the set of node indices of the 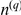 nodes in module 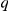. Network concepts (such as the connectivity, clustering coefficient, MAR etc) defined for 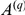 are defined as intramodular network concepts. For example, the density of module 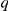 is defined as the mean adjacency of 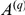:
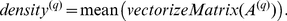
(6)The intramodular connectivity 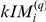 of node 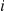 in module 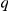 is defined as the sum of connection strengths to other nodes within the same module,
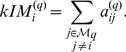
(7)Nodes with high intramodular connectivity are referred to as intramodular hub nodes.

### Module preservation statistics for general networks

Here we describe module preservation statistics that can be used to determine whether a module that is present in a reference network (with adjacency 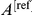) can also be found in an independent test network (with adjacency 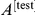). Specifically, assume the vector 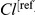 encodes the module assignments in the reference network. Thus 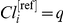 (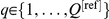) if node 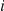 is assigned to module 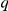. We reserve the label 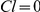 (and color grey) for nodes that are not assigned to any module. For a given module 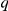 with 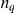 nodes, the 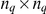 module adjacency matrices are denoted 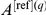 and 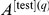 in the reference and test networks, respectively. We propose network concepts that can be useful for determining whether a module 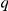 (found in the reference network) is preserved in the test network.

Intuitively, one may call a module 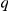 preserved if it has a high density in the test network. We define the *mean adjacency* for module 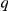 as the module density in the test network,
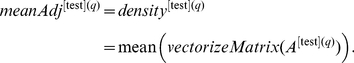
(8)Some of the density statistics such as the mean adjacency are similar to previously described methods based on within-cluster and between-cluster dissimilarities [22]. For example, the mean intramodular adjacency 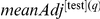 (Equation 8) is oppositely related to the within-module scatter used in assessing the quality of clusters based on a dissimilarity [55]. The network density measure can be considered a generalization of the cluster cohesiveness measure [28] to (possibly weighted) networks.

Other network concepts may be used to obtain a summary statistic of a module. For example, our R function modulePreservation also calculate preservation statistics based on the mean 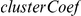 (Equation 5):
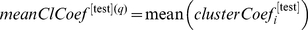
(9)and mean MAR (Equation 4):
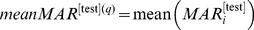
(10)in the test network.

Connectivity preservation statistics quantify how similar connectivity of a given module is between a reference and a test network. For example, module connectivity preservation can mean that, within a given module 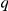, nodes with a high connection strength in the reference network also exhibit a high connection strength in the test network. This property can be quantified by the correlation of intramodular adjacencies in reference and test networks. Specifically, if the entries of the first adjacency matrix 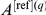 are correlated with those of the second adjacency matrix 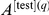 then the adjacency pattern of the module is preserved in the second network. Therefore, we define the *adjacency correlation* of the module 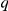 network as
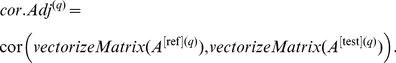
(11)High 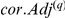 indicates that adjacencies within the module 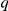 in the reference and test networks exhibit similar patterns.

If module 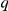 is preserved in the second network, the highly connected hub nodes in the reference network will often be highly connected hub nodes in the test network. In other words, the intramodular connectivity 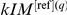 in the reference network should be highly correlated with the corresponding intramodular connectivity 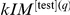 in the test network. Thus, we define the correlation of intramodular connectivities,
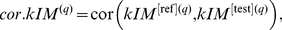
(12)where 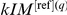 and 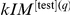 are the vectors of intramodular connectivities of all nodes in module 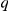 in the reference and test networks, respectively. Analogously, we define the correlation of clustering coefficients and maximum adjacency ratios,
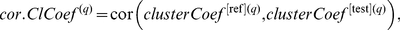
(13)

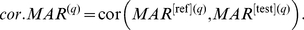
(14)


#### Correlation networks

Correlation networks are a special type of undirected networks in which the adjacency is constructed on the basis of correlations 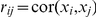 between quantitative measurements that can be described by an 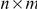 matrix 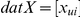 where the column indices correspond to network nodes (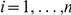) and the row indices (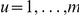) correspond to sample measurements. We refer to the 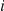-th column 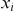 as the 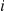-th *node profile* across 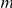 sample measurements. For example, if 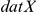 contains data from expression microarrays, the columns correspond to genes (or probes), the rows correspond to microarrays, and the entries report transcript abundance measurements. Networks based on gene expression data are often referred to as gene co-expression networks.

An important choice in the construction of a correlation network concerns the treatment of strong negative correlations. In *signed networks* negatively correlated nodes are considered unconnected. In contrast, in *unsigned networks* nodes with high negative correlations are considered connected (with the same strength as nodes with high positive correlations). As detailed in Supplementary Text S1, a signed weighted adjacency matrix can be defined as follows [5], [56]

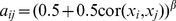
(15)and an unsigned adjacency by
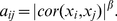
(16)The choice of signed vs. unsigned networks depends on the application; both signed [56] and unsigned [13], [30], [33] weighted gene networks have been successfully used in gene expression analysis. Weighted correlation networks enjoy several advantages over unweighted networks including the following: i) they preserve the continuous nature of the underlying correlation structure; ii) they are highly robust with regard to parameters (e.g. 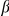) used in the network construction [5], iii) they allow for a geometric interpretation of network concepts [29].

The default method for defining modules in weighted correlation networks is to use average linkage hierarchical clustering coupled with dynamic branch cutting [5], [57].

#### Eigennode summarizes a correlation module and provides a measure of module membership

Many module construction methods lead to correlation network modules comprised of highly correlated variables. For such modules one can summarize the corresponding module vectors using the first principal component denoted by 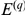 (fifth section of Supplementary Text S1), referred to as the module eigennode (ME) or (in gene co-expression networks) the module eigengene. For example, the gene expression profiles of a given co-expression module can be summarized with the module eigengene [19], [29], [44]. To visualize the meaning of the module eigengene, consider the heat map in Figure 5A. Here rows correspond to genes inside a given module and columns correspond to microarray samples. The heat map color-codes high (red) and low (green) gene expression values. The barplot underneath the heat map visualizes the expression level of the corresponding module eigengene. Note that the module eigengene has a high expression value for samples (columns) where the module genes tend to be over-expressed. The module eigengene can be considered the best summary of the standardized module expression data since it explains the maximum proportion of variance of the module expressions.

The module eigennode 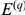 can be used to define a quantitative measure of module membership [29] of node 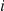 in module 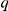:
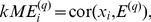
(17)where 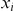 is the profile of node 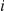. The module membership 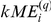 lies in 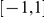 and specifies how close node 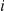 is to module 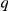. 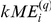 is sometimes referred to as module eigengene-based connectivity [13], [17].

Both *intra*modular network concepts (e.g., 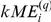) and *inter* modular network concepts (e.g., module separability Equation 27) can be used to study the preservation of network modules. By measuring how these network concepts are preserved from a reference network to a test network, one can define network module preservation statistics as described below.

### Module preservation statistics for correlation networks

The specific nature of correlation networks allows us to define additional module preservation statistics. The underlying information carried by the sign of the correlation can be used to further refine the statistics irrespective of whether a signed or unsigned similarity is used in network construction. To simplify notation, we define
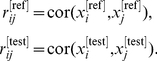
(18)We will use the notation 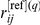 for the correlation matrix restricted to the nodes in module 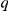. We define the mean correlation density of module 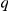 as
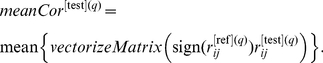
(19)Thus the correlation measure of module preservation is the mean correlation in the test network multiplied by the sign of the corresponding correlations in the reference network. We note that a correlation that has the same sign in the reference and test networks increases the mean, while a correlation that changes sign decreases the mean. Because the preservation statistic keeps track of the sign of the corresponding correlation in the reference network, we call it the mean *sign-aware* correlation.

To measure the preservation of connectivity patterns within module 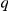 between the reference and test networks, we define a correlation-based measure 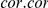 similar to the 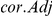 statistic (Equation 11):
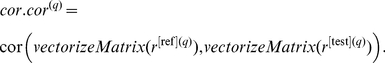
(20)In our applications we find that the correlation-based preservation statistic 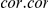 is preferable to its general network counterpart 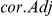; therefore, we only report 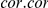.

#### Eigennode-based density preservation statistics

The concept of the module eigennode also gives rise to several preservation statistics that in effect measure module density, or, from a different point of view, how well the eigennode represents the whole module. For example, one can use the proportion of variance explained (defined in the fifth section in Supplementary Text S1) by the module eigennode to arrive at a density measure. In Supplementary Text S1, we prove that the *proportion of variance explained* (PVE) can also be calculated as mean squared 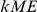 value:
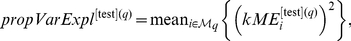
(21)where 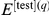 is the eigennode of module 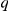 in the test network.

The *mean sign-aware module membership* is defined as
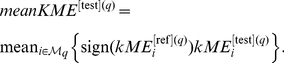
(22)It measures the mean module membership, Equation 17, in which nodes whose module memberships in the reference and test networks have the same sign contribute positively, and nodes whose module memberships in the reference and test networks have opposite signs contribute negatively.

Our statistic 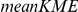 is conceptually related to the homogeneity score [22], [24] which is defined as the average correlation between a cluster's centroid and the members of the cluster. While [24] define the cluster centroid by an average, we use the first principal component (the module eigennode) as cluster centroid since it explains the maximum amount of variation. In several applications, we have found that the use of either cluster centroid leads to very similar results.

#### Eigennode-based connectivity preservation statistics

Intuitively, if the internal structure of a module is preserved between a reference and a test network, we expect that a variable with a high module membership in the reference network will have a high module membership in the test network as well; conversely, variables with relatively low module membership in the reference network should also have a relatively low module membership in the test network. In other words, intramodular hubs in the reference network should also be intramodular hubs in the test network. For a given module 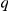 we define the 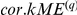 statistic as
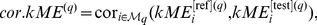
(23)where the correlation runs only over variables that belong to module 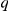. We also define an analogous statistic by correlating the module membership of *all* network variables in the reference and test networks:
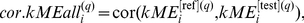
(24)The advantage of using all nodes is that the statistic is less dependent on cutoffs (for example, branch cut parameters) of the method used to define modules. On the other hand, for relatively small modules (compared to the size of the full network) the signal of the few nodes with high module membership may be overwhelmed by the noise contribution of the many nodes that have very low module membership.

#### Module separability statistics

A network module is distinct if it is well separated from the other modules in the network. A distinct module in a reference network may be considered well preserved in a test network if it remains well separated from the other modules in the test network. In the following, we describe several separability based preservation statistics. Denote by 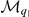 and 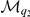 the sets of node indices that correspond to modules 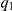 and 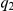, respectively. Our separability statistics contrast *inter* modular adjacencies with *intra*modular adjacencies. To measure the intermodular adjacencies between modules 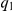 and 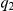, we use
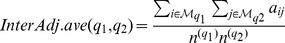
(25)but alternative measures based on the minimal or the maximal intermodular adjacency could also be defined. As measure of mean *intra*modular adjacency in the two modules, we use the geometric mean of the two module densities (Equation 8):
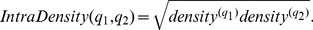
(26)We define separability statistics as 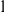 minus the ratio of intermodular adjacency divided by intramodular density:
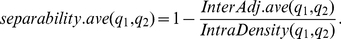
(27)The separability statistics take on (possibly negative) values smaller than 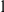. The closer a separability statistic value is to 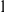, the more separated (distinct) are the two modules. Since 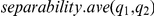 is statistically more robust than the maximum or the minimum based separability measures, it is in general preferable, but in specific applications the minimum and maximum based measures may be useful as well.

In clustering applications based on Euclidean distance it is customary to measure module distinctiveness, or separability, by the between-cluster distance. For correlation networks we propose to measure module separability by 1 minus the correlation of their respective eigennodes. Specifically, for two modules 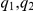, their test separability is defined as
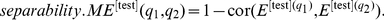
(28)Low test separability suggests the modules are not preserved as separate clusters. Differences in separability between networks may also reflect biologically interesting differences in correlation relationships between whole modules [44].

In the sixth section of Supplementary Text S1, we outline when close relationships exist between 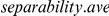 and eigennode based separability 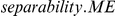. Since the eigennode based separability can be computed much more efficiently, we focus on the eigennode based separability in our applications.

Our separability statistic is conceptually related to the separability score used in [22], [24] which for cluster 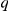 is the weighted average of the correlation between the centroid of cluster 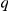 and every other centroid 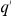,
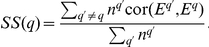
Since we wanted to put all modules on the same footing irrespective of module size, we do not use module size in our definition of the separability statistics. Having said this, it straightforward to adapt our definitions to include module size.

### Assessing significance of observed statistics by permutation tests

Typical values of module preservation statistics depend on many factors, for example on network size, module size, number of observations etc. Thus, instead of attempting to define thresholds for considering a preservation statistic significant, we use permutation tests. Specifically, we randomly permute the module labels in the test network and calculate corresponding preservation statistics. This procedure is repeated 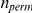 times. For each statistic labeled by index 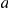 we then calculate the mean 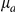 and the standard deviation 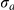 of the permuted values. We define the corresponding 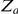 statistic as
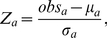
(29)where 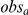 is the observed value for the statistic 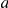. Under certain conditions, one can prove that under the null hypothesis of no preservation the statistic 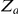 asymptotically follows the standard normal distribution 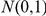. Thus, under the assumption that the number of permutations is large enough to approximate the asymptotic regime, one can convert the 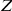 statistics to p-values using the standard normal distribution. Our R function modulePreservation outputs the asymptotic p-values for each statistic. But we should point out that it would be preferable to use a full permutation test to calculate permutation test p-values. We often report Z statistics (instead of p-values) for the following two reasons: First, permutation p-values of preserved modules are often astronomically significant (say 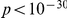) and it is more convenient to report the results on a Z scale. The second reason is computational speed. The calculation of a Z statistic only requires one to estimate the mean and variance under the null hypothesis, for which fewer permutations are needed. To estimate a permutation test p-value accurately would require computational time far beyond practical limits.

### Composite preservation statistic 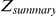 for correlation networks

In the sixth section of Supplementary Text S1, we describe when close relationships exist between many of the preservation statistics presented above. This suggests that one can combine the individual preservation statistics into a composite preservation statistic. We propose two composite preservation statistics. The first composite statistic 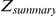 (Equation 1) summarizes the individual Z statistic values that result from the permutation test. The second composite statistic 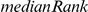 (Equation 34) summarizes the ranks of the observed preservation statistics.

The relationships derived in Supplementary Text S1 suggest to summarize the density based preservation statistics as follows:
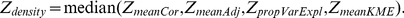
(30)Similarly, the connectivity based preservation statistics can be summarized as follows:
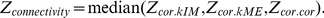
(31)When density and connectivity based preservation statistics are equally important for judging the preservation of a network module, one can consider the composite 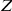 summary statistic (Eq. 1)
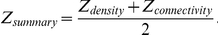
Alternatively, a weighted average between 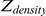 and 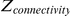 can be formed to emphasize different aspects of module preservation. Future research could investigate alternative ways of aggregating preservation statistics. While our simulations and applications show that 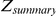 works well for distinguishing preserved from non-preserved modules, we do not claim that it is optimal. In practice, we recommend to consider all individual preservation statistics.

Our simulated as well as empirical data show that the separability tends to have low agreement (as measured by correlation) with the other preservation statistics (Figure 8). Since the 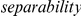 statistic often performs poorly, we did not include it in our composite statistics.

### Calculating empirical p-values for module preservation

Since 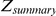 is not a permutation statistic but rather the median of other 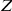 statistics, we do not use it to calculate a p-value. Instead, the R function modulePreservation calculates a summary p-value (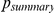) as follows. For each permutation Z statistic, it calculates the corresponding p-value assuming that, under the null, the Z statistic has a normal distribution 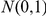. The normal distribution can be justified using relatively weak assumptions described in statistics textbooks. As a caveat, we mention that we use preservation p-values as descriptive (and not inferential) measures. On the other hand, we cannot assume normality for 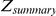. Hence, instead of calculating a p-value corresponding to 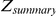, we calculate a summary log-p-value instead, given as the median of the log-p-values of the corresponding permutation 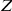 statistics. Because of the often extremely significant p-values associated with the permutation 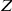 statistics, it is desirable to use logarithms (here base 10). We emphasize that the summary log-p-value is not directly associated with 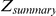; rather, it is a separate descriptive summary statistic that summarizes the p-values of the individual permutation 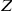 statistics.

### Thresholds for 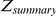


It seems intuitive to call a module with 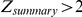 preserved, but our simulation studies argue for a more stringent threshold. We recommend the following threshold guidelines: if 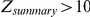, there is strong evidence that the module is preserved. If 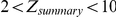 there is weak to moderate evidence of preservation. If 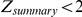, there is no evidence that the module preserved. As discussed below, these threshold values should be considered rough guidelines since more (or less) stringent thresholds may be required depending on the application.

The modulePreservation R function calculates multiple preservation 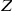 statistics and corresponding asymptotic p-values. Similar to the case of 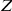 statistics, a threshold that is appropriate in one context may not be appropriate in another. The choice of thresholds depends not only on the desired significance level but also on the research question. When several preservation statistics are analyzed individually for any indication of module preservation then the threshold should correct for the these multiple comparisons. Since several “tests” for preservation are considered, an obvious choice is to use one of the standard correction approaches (e.g., Bonferroni correction) for determining the threshold that should be put on multiple tests. Toward this end, one can use the uncorrected, individual preservation statistics and p-values output by the modulePreservation function. A Bonferroni correction would be a conservative but probably too stringent approach in light of the fact that many of the preservation statistics are closely related (see the 6th section in Supplementary Text S1). Given the strong relationships among some preservation statistics, we have found it useful to aggregate the 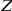 statistics (and optionally the empirical p-values) in a statistically robust fashion using the median but many alternative procedures are possible. To provide some guidance, we recommend thresholds for 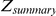 that we have found useful in our simulations studies (Supplementary Text S6) and in our empirical studies.

### Composite preservation statistic 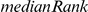


In some applications such as the human vs. chimpanzee comparison described above, one is interested in ranking modules by their overall preservation in the test set, i.e., one is interested in a relative measure of module preservation. Since our simulations and applications reveal that 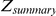 (Equation 1) strongly depends on module size, this statistic may not be appropriate when comparing modules of very different sizes. Here we define an alternative rank-based measure that relies on observed preservation statistics rather than the permutation 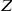 statistics. For each statistic 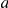, we rank the modules based on the observed values 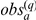. Thus, each module is assigned a rank 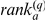 for each observed statistic. We then define the median density and connectivity ranks
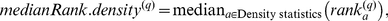
(32)

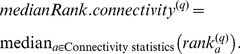
(33)Analogously to the definition of 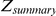, we then define the statistic 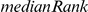 as the mean of 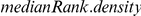 and 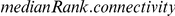,
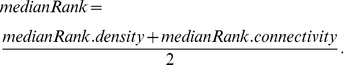
(34)Alternatively, a weighted average of the ranks could be formed to emphasize different aspects of module preservation. It is worth repeating that a composite rank preservation statistic is only useful for studying the relative preservation of modules, e.g., we use 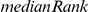 for studying which human brain co-expression modules are least preserved in chimpanzee brain networks.

### Composite preservation statistic 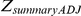 for general networks

While all examples in this article relate to correlation (in particular, co-expression) networks, we have also implemented methods and R function that can be applied to general networks specified only by an adjacency matrix. For example, this function could be used to study module preservation in protein-protein interaction networks. We also define a composite statistic 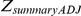, which is defined for a general network specified by an adjacency matrix (Eq. 35).
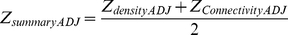
(35)where 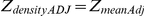 and 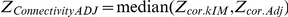. Note that 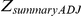 is only computed with regard to a subset of the individual statistics. To invoke this preservation statistic, set dataIsExpr = FALSE in the modulePreservation R function.

### Detailed methods description in Supplementary Text S1

A detailed description of the methods is provided Supplementary Text S1 which contains the following sections. In the first section of Supplementary Text S1, we describe standard cross-tabulation based module preservation statistics. Specifically, we present three basic cross-tabulation based statistics for determining whether modules in a reference data set are preserved in a test data set. These statistics do not assume that a test network is available. Instead, module assignments in both the reference and the test networks are needed.

In the second section, we briefly review a hierarchical clustering procedure for module detection. Many methods exist for defining network modules. In this section, we describe the method used in our applications but it is worth repeating that our preservation statistics apply to most alternative module detection procedures.

In the third section, we review the definition of signed and unsigned correlation networks. Correlation networks are a special case of general undirected networks in which the adjacency is constructed on the basis of correlations between quantitative variables.

In the fourth section, we present module quality statistics, which we are implemented in the modulePreservation R function. While our main article focuses on statistics that measure preservation of modules between a reference and a test network, we briefly discuss the application of some of the preservation statistics to the related but distinct task of measuring module quality in a single (reference) network. More precisely, the density and separability statistics can be applied to the reference network without a reference to a test network. The results can then be interpreted as measuring module quality, that is how closely interconnected the nodes of a module are or how well a module is separated from other modules in the network.

In the fifth section, we review the notation for the singular value decomposition and for defining a module eigennnode. The section describes conditions when the eigenvector 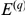 is an optimal way of representing a correlation module. It also reviews the definition of 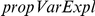 (the proportion of the variance explained by the eigennode). We derive a relationship between 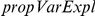 and the module membership measures 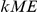, which will be useful for deriving relationships between preservation statistics.

In the sixth section, we investigate relationships between preservation statistics in correlation networks.

### Brief overview of KEGG pathways studied in Application 3

The KEGG database and many textbooks describe these fundamental pathways in more detail but the following terse descriptions may be helpful. The Wnt signaling pathway describes a network of proteins most well known for their roles in embryogenesis and cancer, but also involved in normal physiological processes in adult animals. The Hedgehog signaling pathway is one of the key regulators of animal development conserved from flies to humans. The apoptosis pathway mediates programmed cell death. Endocytosis is the process by which cells absorb molecules (such as proteins) from outside the cell by engulfing them with their cell membrane. The Transforming growth factor beta (TGF-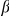) signaling pathway is involved in many cellular processes in both the adult organism and the developing embryo including cell growth, cell differentiation, apoptosis, cellular homeostasis and other cellular functions. The Phosphatidylinositol signaling system facilitates environmental information processing and signal transduction. The mitogen-activated protein kinase (MAPK) cascade is a highly conserved pathway that is involved in various cellular functions, including cell proliferation, differentiation and migration. The Calcium signaling pathway describes how calcium can act in signal transduction after influx resulting from activation of ion channels, or as a second messenger caused by indirect signal transduction pathways such as G protein-coupled receptors.

## Supporting Information

Table S1 Preservation statistics of human brain modules in chimpanzee samples and vice-versa. This table contains observed preservation statistics and their permutation Z scores of human brain modules in chimpanzee samples and vice-versa. Columns indicate the reference data set, test data set, module label (color), module type, module size, observed preservation statistics, their Z scores, empirical p-values, and Bonferoni-corrected empirical p-values. The grey (improper) modules contain all unassigned genes, and the gold module is a random sample representing the entire network as a single module.
(0.03 MB CSV)

Table S2 Preservation statistics of male human brain modules in the corresponding female samples and vice-versa. This table contains observed preservation statistics and their permutation Z scores of male human brain modules in the corresponding female samples and vice-versa. Columns indicate the reference data set, test data set, module label (color), module type, module size, observed preservation statistics, their Z scores, empirical p-values, and Bonferoni-corrected empirical p-values. The grey (improper) modules contain all unassigned genes, and the gold module is a random sample of genes representing the entire network as a single module.
(0.26 MB CSV)

Table S3 Preservation statistics of female mouse liver modules in the corresponding male samples. This table contains observed preservation statistics and their permutation Z scores of female mouse liver modules in the corresponding male samples. Columns indicate the reference data set, test data set, module label (color), module size, observed preservation statistics, their Z scores, empirical p-values, and Bonferoni-corrected empirical p-values.
(0.02 MB CSV)

Table S4 Preservation statistics of consensus modules across the data sets in which they were identified. This table contains observed preservation statistics and their permutation Z scores of consensus modules across the data sets from which the consensus modules were obtained. Columns indicate the reference data set, test data set, module label (color), module type, module size, observed preservation statistics, their Z scores, empirical p-values, and Bonferoni-corrected empirical p-values. The grey (improper) modules contain all unassigned genes, and the gold module is a random sample representing the entire network as a single module.
(0.34 MB CSV)

Table S5 Preservation statistics of simulated modules. This table contains observed preservation statistics and their permutation Z scores of simulated modules in our simulation studies. Columns indicate simulation model, module label, simulated status (preserved or non-preserved), observed preservation statistics, Z scores, empirical p-values, and Bonferoni-corrected empirical p-values. The grey (improper) modules contain all unassigned genes, and the gold module is a random sample representing the entire network as a single module.
(0.16 MB CSV)

Text S1 Detailed methods description. A detailed description of the methods is provided which contains the following sections. First, we describe standard cross-tabulation based module preservation statistics. Specifically, we present three basic cross-tabulation based statistics for determining whether modules in a reference data set are preserved in a test data set. These statistics do not assume that a test network is available. Instead, module assignments in both the reference and the test networks are needed. Second, we briefly review a hierarchical clustering procedure for module detection. Many methods exist for defining network modules. In this section, we describe the method used in our applications but it is worth repeating that our preservation statistics apply to most alternative module detection procedures. Third, we review the definition of signed and unsigned correlation networks. Correlation networks are a special case of general undirected networks in which the adjacency is constructed on the basis of correlations between quantitative variables. Fourth, we present module quality statistics, which we are implemented in the modulePreservation R function. While our main article focuses on statistics that measure preservation of modules between a reference and a test network, we briefly discuss the application of some of the preservation statistics to the related but distinct task of measuring module quality in a single (reference) network. More precisely, the density and separability statistics can be applied to the reference network without a reference to a test network. The results can then be interpreted as measuring module quality, that is how closely interconnected the nodes of a module are or how well a module is separated from other modules in the network. Fifth, we review the notation for the singular value decomposition and for defining a module eigennnode. The section describes conditions when the eigenvector E is an optimal way of representing a correlation module. It also reviews the definition of the proportion of the variance explained by the eigennode). We derive a relationship between PVE and the module membership measures kME, which will be useful for deriving relationships between preservation statistics. Sixth, we investigate relationships between preservation statistics in correlation networks. An advantage of an (unsigned) weighted correlation network is that it allows one to derive simple relationships between network concepts (Horvath and Dong 2008). We characterize correlation modules where simple relationships exist between i) density-based preservation statistics, ii) connectivity based preservation statistics, and iii) separability based preservation statistics. Apart from studying relationships among preservation statistics in correlation networks, we also briefly describe relationships between preservation statistics in general networks.
(0.17 MB PDF)

Text S2 Details regarding module preservation between human and chimpanzee brain networks. In this document we provide detailed results regarding the preservation of human brain modules in chimpanzee brains.
(0.22 MB PDF)

Text S3 Detailed description of the human brain. In this document we provide detailed results of Application 4: Preservation of cortical modules between male and female samples.
(2.51 MB PDF)

Text S4 Detailed description of female mouse liver modules in male mice. Detailed results of Application 5: Preservation of female mouse liver modules in male mice.
(3.82 MB PDF)

Text S5 Detailed description of the consensus module application. Here we study preservation of consensus modules constructed previously, namely the consensus modules across human and chimpanzee brain samples, across samples from 4 tissues of female mice, and across samples from male and female mouse livers.
(1.41 MB PDF)

Text S6 Detailed description of the simulation study. Detailed performance analysis of the proposed module preservation statistics in seven simulation scenarios. The design and main results of the simulations are summarized in Figure 9 of the main text.
(2.78 MB PDF)
