# Supplementary material for: Is My Network Module Preserved and Reproducible?
Source: PLoS Comput Biol. 2011 Jan 20;7(1):e1001057. doi: 10.1371/journal.pcbi.1001057 (PMC3024255; doi:10.1371/journal.pcbi.1001057)
Supplement: Text S1 — Detailed methods description. A detailed description of the methods is provided which contains the following sections. First, we describe standard cross-tabulation based module preservation statistics. Specifically, we present three basic cross-tabulation based statistics for determining whether modules in a reference data set are preserved in a test data set. These statistics do not assume that a test network is available. Instead, module assignments in both the reference and the test networks are needed. Second, we briefly review a hierarchical clustering procedure for module detection. Many methods exist for defining network modules. In this section, we describe the method used in our applications but it is worth repeating that our preservation statistics apply to most alternative module detection procedures. Third, we review the definition of signed and unsigned correlation networks. Correlation networks are a special case of general undirected networks in which the adjacency is constructed on the basis of correlations between quantitative variables. Fourth, we present module quality statistics, which we are implemented in the modulePreservation R function. While our main article focuses on statistics that measure preservation of modules between a reference and a test network, we briefly discuss the application of some of the preservation statistics to the related but distinct task of measuring module quality in a single (reference) network. More precisely, the density and separability statistics can be applied to the reference network without a reference to a test network. The results can then be interpreted as measuring module quality, that is how closely interconnected the nodes of a module are or how well a module is separated from other modules in the network. Fifth, we review the notation for the singular value decomposition and for defining a module eigennnode. The section describes conditions when the eigenvector E is an optimal way of representing a cor [file pcbi.1001057.s006.pdf]

Supplementary text S1:
Methods description and relationships between module preservation statistics

Peter Langfelder, Luo Rui, Michael C. Oldham, and Steve Horvath*
*Corresponding author: shorvath@mednet.ucla.edu

Contents of the Supplementary Text

Here, we provide additional methodological details regarding the module preservation statistics. In the first section, we describe standard cross-tabulation based module preservation statistics. Specifically, we present three basic cross-tabulation based statistics for determining whether modules in a reference data set are preserved in a test data set. These statistics do not assume that a test network is available. Instead, module assignments in both the reference and the test networks are needed.

In the second section, we briefly review a hierarchical clustering procedure for module detection. Many methods exist for defining network modules. In this section, we describe the method used in our applications but it is worth repeating that our preservation statistics apply to most alternative module detection procedures.

In the third section, we review the definition of signed and unsigned correlation networks. Correlation networks are a special case of general undirected networks in which the adjacency is constructed on the basis of correlations between quantitative variables.

In the fourth section, we present module quality statistics that we are implemented in the `modulePreservation` R function. While our main article focuses on statistics that measure preservation of modules between a reference and a test network, we briefly discuss the application of some of the preservation statistics to the related but distinct task of measuring module quality in a single (reference) network. More precisely, the density and separability statistics can be applied to the reference network without a reference to a test network. The results can then be interpreted as measuring module quality, that is how closely interconnected the nodes of a module are or how well a module is separated from other modules in the network.

In the fifth section, we review the notation for the singular value decomposition and for defining a module eigennnode. The section describes conditions when the eigenvector $E^{(q)}$ is an optimal way of representing a correlation module. It also reviews the definition of *propVarExpl* (the proportion of the variance explained by the eigennode). We derive a relationship between *propVarExpl* and the module membership measures *kME*, which will be useful for deriving relationships between preservation statistics.

In the sixth section, we investigate relationships between preservation statistics in correlation networks. An advantage of an (unsigned) weighted correlation network is that it allows one to derive simple relationships between network concepts [1, 2]. We characterize correlation modules where simple relationships exist between i) density-based preservation statistics, ii) connectivity based preservation statistics, and iii) separability based preservation statistics. Apart from studying relationships among preservation statistics in correlation networks, we also briefly describe relationships between preservation statistics in general networks.

In the seventh section we briefly review the In-Group Proportion method [3].

1 Cross-tabulation based module preservation statistics

Here we present three basic cross-tabulation based statistics for determining whether modules in a reference data set are preserved in a test data set. These statistics do not assume that a test network is available. Instead, module assignments in both the reference and the test networks are needed. For each object i, $Cl_i^{[\mathrm{ref}]}$ denotes the module label in the reference module assignment. The module are labeled by $q = 1, 2, \ldots, Q^{[\mathrm{ref}]}$ where $Q^{[\mathrm{ref}]}$ is the number of modules in the reference set. The number of objects in module q will be denoted by $n^{(q)}$. Assume that the module

assignment of the test data ($Cl^{[\text{test}]}$) leads to $Q^{[\text{test}]}$ modules labelled by $q' = 1, 2, \ldots, Q^{[\text{test}]}$. The goal is to determine whether a module q in the reference clustering $Cl^{[\text{ref}]}$ can be matched to a module q' in the test clustering $Cl^{[\text{test}]}$. Since many cross tabulation based statistics have been described in the context of cluster preservation and validation procedures (a review can be found in [3]), we will use the words "cluster" and "module" interchangeably in this section.

For each module specified in $Cl^{[\text{ref}]}$, a cross-tabulation based preservation statistic defines a value. As convention, we assume that the higher the preservation value of cluster (module) q, the stronger the evidence that it corresponds to a cluster specified in $Cl^{[\text{test}]}$. Here we will describe two approaches that are based on cross-tabulating $Cl^{[\text{ref}]}$ and $Cl^{[\text{test}]}$, that is creating a contingency table in which every row corresponds to a reference module, and each column to a test module.

We start with a module preservation statistic based on keeping track of the co-clustering of pairs of objects. For the q-th module of $Cl^{[\text{ref}]}$, one can form $\binom{n^{(q)}}{2} = \frac{n^{(q)}\,(n^{(q)}-1)}{2}$ different pairs of objects. Let $n_{qq'}$ denote the number of objects which are in the q-th module of $Cl^{[\text{ref}]}$ and in the q'-th module of $Cl^{[\text{test}]}$. The number of *pairs* of objects in the q-th module of $Cl^{[\text{ref}]}$ that are also part of the q'-th module of $Cl^{[\text{test}]}$ is given by $\binom{n_{qq'}}{2}$. The proportion of pairs of objects in module q that also cluster in module q' is given by

$$propCoClustering(q, q') = \binom{n_{qq'}}{2} \Big/ \binom{n^{(q)}}{2} .$$

Apart from pairs of objects ($tupletsize = 2$), one can also calculate the proportion of triplets ($tupletsize = 3$) or quadruplets of objects ($tupletsize = 4$) from the q-th module of $Cl^{[\text{ref}]}$ that co-cluster in the q'-th module of $Cl^{[\text{test}]}$:

$$propCoClustering(q, q', tupletsize) = \binom{n_{qq'}}{tupletsize} \Big/ \binom{n^{(q)}}{tupletsize} .$$

Using the above notation, we define the co-clustering based module preservation statistic as

$$coClusteringPreservation(q, tupletsize) = \sum_{q'=1}^{Q^{[\text{test}]}} propCoClustering(q, q', tupletsize) , \tag{1}$$

which depends on the tuplet size. For the case $tupletsize = 2$ (pairs) the co-clustering preservation statistic is related to the prediction strength statistic in [3, 4].

An alternative cross-tabulation statistic is the accuracy and the related Fisher exact test p-value. For each proper reference module q with n_q objects we find the proper test module q' with the highest number of objects common to both the reference and the test module, $n_{qq'}$. We define the *accuracy* of the reference module q in the test clustering as

$$accuracy_q = \frac{n_{qq'}}{n_q} . \tag{2}$$

By definition, the accuracy lies in $[0, 1]$, and $accuracy_q = 1$ indicates that all objects that form the reference module q are part of the same module in the test network. We emphasize that this definition is only used for the proper modules. For the improper module that contains all objects not assigned to any of the proper modules (labeled by the label 0), we define its accuracy as

$$accuracy_0 = \frac{n_{00'}}{n_0} . \tag{3}$$

where n_0 is the number of unassigned objects, and $n_{00'}$ is the number of objects unassigned both in the reference and in the test clusterings.

A potential disadvantage of the accuracy measure is that it does not take into account how likely it is to observe a particular maximum overlap by chance. As an example, consider a case in which there are say 10 clusters in the reference clustering, and a test clustering in which the clusters are perfectly reproduced, so the accuracy of all 10 reference clusters equals 1. Now consider another test clustering in which all objects belong to a single cluster. The accuracies of all clusters with respect to the second test clustering again equal 1, but intuitively the second test clustering is less interesting than the first clustering. To address this issue, we define a p-value based preservation statistic as follows. For each reference-test pair q, q' of proper modules, we calculate the one-sided Fisher p-value of

the observed overlap of the two modules, $p_{qq'}$. For each proper reference module q, we then report minus the logarithm of the lowest (most significant) p-value:

$$mlfp_q = -\log\left(\min_{q'} p_{qq'}\right) . \tag{4}$$

For the improper module of unassigned variables that carry the label 0 we define

$$mlfp_0 = -\log p_{00'} . \tag{5}$$

2 Using hierarchical clustering for module detection

Many methods exist for defining network modules; here we describe the method used in our applications. We define modules as clusters of "similar" nodes. To measure the dissimilarity of nodes, one can define the adjacency matrix based dissimilarity:

$$dissA_{ij} = 1 - a_{ij} . \tag{6}$$

However, more elaborate dissimilarities can be constructed from an adjacency. For example, several measures of network interconnectedness are described in [5]. In our applications and simulations we use the topological overlap measure [5–8] as input to hierarchical clustering [9]; branches of the hierarchical clustering dendrogram correspond to modules and can be identified using one of a number of available branch cutting methods, for example the constant-height cut or two Dynamic Branch Cut methods [10]. Our branch cutting algorithm only assigns module labels to branches whose size exceeds a user-specified threshold parameter. Not all nodes necessarily belong to a module. Nodes that do not belong to a module are called "unassigned" and conventionally carry the label 0. Thus, the clustering results in assigning a module (or zero) label Cl_i to each node i. In practice, it is advisable to vary the minimum module size and other branch cutting parameters to determine how the results are affected by different parameter choices. This module detection approach has led to biologically meaningful modules in several applications [1, 7, 11–15].

3 Signed and unsigned correlation networks

Methods surrounding the weighted correlation network analysis are implemented in the R package `WGCNA` [16]. Correlation networks are a special case of general undirected networks in which the adjacency is constructed on the basis of correlations between quantitative measurements that can be described by an $n \times m$ matrix $datX = [x_{ui}]$ where the column indices correspond to network nodes ($i = 1, \ldots, n$) and the row indices ($u = 1, \ldots, m$) correspond to sample measurements:

$$datX = [x_{ui}] = (x_1 x_2 \cdots x_n) . \tag{7}$$

We refer to the i-th column x_i as the i-th *node profile* across m sample measurements.

A correlation network adjacency matrix is constructed on the basis of the pairwise correlations $\mathrm{cor}(x_i, x_j)$ between the columns of $datX$. We distinguish two types of correlation network adjacencies, signed and unsigned. The elements of an unsigned adjacency matrix can be written as a non-decreasing function of the absolute correlations, i.e. $a_{ij} = nonDecreasingF(|\mathrm{cor}(x_i, x_j)|)$. In contrast, the elements of a signed adjacency matrix can be written as a non-decreasing function of the correlation, i.e. $a_{ij} = nonDecreasingF(\mathrm{cor}(x_i, x_j))$. These non-decreasing functions are required to yield adjacencies that continue to satisfy our conditions imposed on a general adjacency matrix (in particular the components are required to lie within $[0, 1]$), but are otherwise in principle arbitrary.

We define the unsigned and signed adjacencies in terms of a general non-decreasing function *nonDecreasingF* of $|\mathrm{cor}(x_i, x_j)|$ and $\mathrm{cor}(x_i, x_j)$, respectively. Several forms of the non-decreasing function have been proposed in the literature. For example, a common choice is the step function with threshold τ,

$$nonDecreasingF(s) = \begin{cases} 0 & \text{for } s < \tau \\ 1 & \text{for } s \geq \tau \end{cases} . \tag{8}$$

This approach to constructing the adjacency is known as hard-thresholding and leads to an unweighted network in which the adjacency only takes on values 0 (unconnected) or 1 (connected). In contrast, in our applications we use a continuous function that results in a weighted network. The following *nonDecreasingF* leads to an unsigned adjacency:

$$a_{ij}^{unsigned} = |\mathrm{cor}(x_i, x_j)|^{\beta} , \tag{9}$$

that is

$$nonDecreasingF^{unsigned}(s) = s^{\beta}\,. \tag{10}$$

In contrast, the signed adjacency is given by

$$a_{ij}^{signed} = \left(\frac{1+\mathrm{cor}(x_i, x_j)}{2}\right)^{\beta}\,, \tag{11}$$

that is

$$nonDecreasingF^{signed}(s) = \left(\frac{1+s}{2}\right)^{\beta}\,. \tag{12}$$

The choice of signed versus unsigned networks depends on the application; both signed [17] and unsigned [18] gene networks have been successfully used in gene expression analysis. The above definitions of adjacency can be modified, for example, by replacing the Pearson correlation by an outlier-resistant correlation. When dealing with large networks (comprised of thousands of genes) one can use default choices for the power β: 6 for an unsigned network and 12 for a signed network. These powers implement a soft-thresholding approach. An advantage of weighted networks is that the network construction is highly robust with regard to the choice of β [7]. The default choices are implemented in our R function `modulePreservation`. The default choices have worked in many publications but one can also develop criteria heuristics for choosing a threshold. For example, [7] proposed the scale free topology criterion for choosing β.

4 Module quality statistics defined for the reference network

In most of this work we have focused on statistics that measure preservation of modules between a reference and a test network. In this section we briefly discuss the application of some of the preservation statistics to the related but distinct task of measuring module quality in a single (reference) network. More precisely, the density and separability statistics can be applied to the reference network without a reference to a test network. The results can then be interpreted as measuring module quality, that is how closely interconnected the nodes of a module are or how well a module is separated from other modules in the network. In Table 1, we provide an overview of the input required to calculate each of the module quality statistics presented here. All statistics require the module assignment (label) from the reference data and output a quality statistic based on the reference data (i.e., no test set is used). Specifically, the module quality statistics are given by

$$\begin{aligned}
meanAdj^{[\mathrm{ref}](q)} &= \mathrm{mean}\left(vectorizeMatrix(A^{[\mathrm{ref}](q)})\right)\,, && (13)\\
meanCor^{[\mathrm{ref}](q)}_{unsigned} &= \mathrm{mean}\left\{vectorizeMatrix\left(|r_{ij}^{[\mathrm{ref}](q)}|\right)\right\}\,, && (14)\\
meanCor^{[\mathrm{ref}](q)}_{signed} &= \mathrm{mean}\left\{vectorizeMatrix\left(r_{ij}^{[\mathrm{ref}](q)}\right)\right\}\,, && (15)\\
meanKME^{[\mathrm{ref}](q)}_{unsigned} &= \mathrm{mean}_{i\in\mathcal{M}_q}\left\{|kME_i^{[\mathrm{ref}](q)}|\right\}\,, && (16)\\
meanKME^{[\mathrm{ref}](q)}_{signed} &= \mathrm{mean}_{i\in\mathcal{M}_q}\left\{kME_i^{[\mathrm{ref}](q)}\right\}\,, && (17)\\
propVarExpl^{[\mathrm{ref}](q)} &= \mathrm{mean}_{i\in\mathcal{M}_q}\left\{(kME_i^{[\mathrm{ref}](q)})^2\right\}\,, && (18)\\
separability^{[\mathrm{ref}]}(q_1,q_2) &= 1-\mathrm{cor}(E^{[\mathrm{ref}](q_1)}, E^{[\mathrm{ref}](q_2)})\,. && (19)
\end{aligned}$$

The mean adjacency (Equation 13) applies to a general network while the remaining statistics assume a correlation network. The *meanCor* and *meanKME* statistics each have slightly different versions for signed and unsigned correlation networks.

Thus the correlation statistic of module quality is simply the mean of (the absolute values of) the variable-variable correlations within the module. Low reference separability may suggest that the two modules q_1, q_2 are not really distinct and should be merged.

5 Singular value decomposition and module eigengenodes

Assume an $m \times n$ dimensional matrix $datX$ whose i-th column x_i is a numeric vector with m components. The **singular value decomposition (SVD)** of $datX$ is the decomposition

$$datX = UDV^T, \tag{20}$$

where U and V are matrices of **left** and **right singular vectors**, respectively, and the matrix D is a diagonal matrix that contains the **singular values** $|d_1|, |d_2|, \ldots$:

$$\begin{aligned} U &= (u_1 u_2 \ldots u_{\min(m,n)}) \\ V &= (v_1 v_2 \ldots v_{\min(m,n)}) \\ D &= diag\{|d_1|, |d_2|, \ldots, |d_{\min(m,n)}|\}. \end{aligned} \tag{21}$$

We use the absolute value sign around the singular values to remind the reader that the singular values are *non-negative* real numbers. In the following, we assume that the singular values are arranged in a decreasing order so $|d_1|$ is the largest value. The $m \times \min(m,n)$ dimensional matrix U and the $n \times \min(m,n)$ dimensional matrix V contain orthonormal columns. Non-degenerate singular values always have unique left and right singular vectors, up to multiplication by a sign. Consequently, if all singular values of $datX$ are non-degenerate and non-zero, its singular value decomposition is unique, up to multiplication of a column of U by a sign and simultaneous multiplication of the corresponding column of V by the same sign. In applications the first singular value $|d_1|$ is typically non-degenerate. In this case, u_1 and v_1 are uniquely defined up to a sign. In practice, we fix the sign of u_1 by requiring that its average correlation with the columns of $datX$ is positive.

In the following, we describe an important use of the left singular vectors of a singular value decomposition. Since it is widely used when dealing with network modules (which represent clusters of vectors), we find convenient to introduce notation for the case where the numeric vectors represent a subset of the original set of vectors. Thus the columns of the $m \times n^{(q)}$ matrix $datX^{(q)}$ represent a subset of the original n vectors. We typically scale the columns of $datX^{(q)}$ so that they have mean zero ($\text{mean}(x_i) = 0$) and $\text{mean}((x_i)^2) = 1$. The singular value decomposition

$$datX^{(q)} = U^{(q)} D^{(q)} (V^{(q)})^T. \tag{22}$$

provides an $m \times \min(m, n^{(q)})$ dimensional matrix $U^{(q)}$ of left singular vectors. The sign of the first left singular vector $u_1^{(q)}$ is fixed by requiring that its average correlation with the columns of $datX^{(q)}$ is positive. When $datX^q$ corresponds to the gene expression data of a network module, $u_1^{(q)}$ (the first column of $U^{(q)}$) is referred to as the **module eigengene**. More generally, we refer to the vector

$$E^{(q)} = u_1^{(q)}$$

as the **eigenvector** or the **eigennode** in the context of a correlation network. While $E^{(q)}$ is in general *not* an eigenvector of $datX^{(q)}$ it turns out to be an eigenvector of the $m \times m$ dimensional matrix $datX^{(q)}(datX^{(q)})^T$ corresponding to the largest eigenvalue. The eigenvector $E^{(q)}$ is an optimal way of summarizing the scaled columns of $datX^{(q)}$ in the sense that it explains the highest amount of the variation in the scaled columns.

Using the fact that the columns are scaled ($\sum_u x_{ui} = 0$ and $\sum_u x_{ui}^2/m = 1$), one can show:

$$\begin{aligned} \text{cor}(x_i, x_j) &= \sum_l v_{l,i} |d_l|^2 v_{l,j}/m, & (23) \\ kME_i &= \text{cor}(x_i, E^{(q)}) = v_{1,i}|d_1|/\sqrt{m}, & (24) \\ \sum_i (kME_i)^2/n^{(q)} &= \frac{|d_1|^2}{mn^{(q)}}. & (25) \end{aligned}$$

The proportion of variance explained by the eigenvector $E^{(q)}$ is given by

$$propVarExpl(E^{(q)}) = \frac{|d_1^{(q)}|^2}{\sum_{j=1}^{\min(m,n^{(q)})} |d_j^{(q)}|^2} = \frac{|d_1|^2}{mn^{(q)}}. \tag{26}$$

We now derive the following relationship between *propVarExpl* (Eq. 26) and the mean squared *kME* value:

$$propVarExpl^{[\text{test}](q)} = \text{mean}_{i \in \mathcal{M}_q} \left\{ \left(kME_i^{[\text{test}](q)} \right)^2 \right\} , \tag{27}$$

where $E^{[\text{test}](q)}$ is the eigennode of module q in the test network. The derivation is rather straightforward:

$$\begin{aligned} propVarExpl^{[\text{test}](q)} &= \frac{|d_1|^2}{mn} = \sum_i \boldsymbol{v}_{1,i}^2 \frac{|d_1|^2}{mn} \\ &= \text{mean}_{i \in \mathcal{M}_q} \left\{ \left(kME_i^{[\text{test}](q)} \right)^2 \right\} \end{aligned}$$

where $E^{[\text{test}](q)}$ is the eigennode of module q in the test network.

The singular value decomposition of $datX^{(q)}$ is closely related to the principal component analysis of the correlation matrix $cor.datX^{(q)} = (\text{cor}(x_i^{(q)}, x_j^{(q)}))$ whose entries correspond to the pairwise correlations between the columns of $datX^{(q)}$. The eigenvalues of the correlation matrix $cor.datX^{(q)}$ are squares of corresponding singular values $|d_l^{(q)}|$. $E^{(q)}$ explains a high proportion of the variation of the scaled columns of $datX^{(q)}$ if these columns have high pairwise correlations.

6 Relationships preservation statistics in correlation networks

One can derive theoretical relationships between preservations statistics in the case of approximately factorizable adjacency matrices ($a_{ij} \approx CF_i\, CF_j$) and correlation modules ($\text{cor}(x_i, x_j) \approx kME_i\, kME_j$) [1,2]. In particular, the geometric interpretation of correlation networks [2] shows when close relationship exist among the density based preservation statistics (*meanCor*, *meanAdj*, *propVarExpl*, *meanKME*), among the connectivity based preservation statistics (*cor.kIM*, *cor.kME*, *cor.kMEall*, *cor.cor*), and between the separability statistics (*separability.ave*, *separability.ME*). In the following, we briefly outline how to derive relationships.

Relationships among density preservation statistics

A theoretical advantage of an (unsigned) weighted correlation network with adjacency $a_{ij} = |\text{cor}(x_i, x_j)|^\beta$ (Eq. 9) is that it allows one to derive simple relationships between network concepts [1, 2]. In [2], it is shown that correlation module networks with high **eigennode factorizability** $EF(E^{(q)})$, where

$$EF(E^{(q)}) = \frac{|d_1^{(q)}|^4}{\sum_j |d_j^{(q)}|^4} , \tag{28}$$

lead to an approximately factorizable correlation matrix

$$\begin{aligned} cor(x_i^{(q)}, x_j^{(q)}) &\approx \text{cor}(x_i^{(q)}, E^{(q)}) \, \text{cor}(x_j^{(q)}, E^{(q)}) \\ &\approx kME_i \, kME_j \, . \end{aligned} \tag{29}$$

We now show that $EF(E^{(q)}) \approx 1$ implies that mean correlation

$$meanCor^{[\text{test}](q)} = \text{mean} \left\{ vectorizeMatrix \left(\text{sign}(r_{ij}^{[\text{ref}](q)}) r_{ij}^{[\text{test}](q)} \right) \right\} \tag{30}$$

of a correlation module that contains only positively-correlated genes is proportional to the proportion of variance explained *propVarExpl* (Eq. 27). Then

$$\begin{aligned}
meanCor &= \frac{1}{n^{(q)}(n^{(q)}-1)}\sum_{i=1}^{n^{(q)}}\sum_{j\neq i}^{n^{(q)}} cor(x_i,x_j) \\
&\approx \frac{1}{(n^{(q)})^2}\sum_{i=1}^{n^{(q)}}\sum_{j=1}^{n^{(q)}} cor(x_i,x_j) \\
\text{(by approximate factorizability)} &\approx \frac{1}{(n^{(q)})^2}\sum_{i=1}^{n^{(q)}}\sum_{j=1}^{n^{(q)}} cor(x_i,E^{(q)})\mathrm{cor}(x_j,E^{(q)}) \\
&\approx \frac{1}{(n^{(q)})^2}\sum_{i=1}^{n^{(q)}}\sum_{j=1}^{n^{(q)}} \boldsymbol{v}_{1,i}\boldsymbol{v}_{1,j}|d_1|^2/m \\
&= \left(\frac{\sum_{i=1}^{n^{(q)}}\boldsymbol{v}_{1,i}}{\sqrt{n^{(q)}}}\right)^2\frac{|d_1|^2}{mn^{(q)}} = \left(\frac{\sum_{i=1}^{n^{(q)}}\boldsymbol{v}_{1,i}}{\sqrt{n^{(q)}}}\right)^2 propVarExpl \\
&= (cos(\theta^{(q)}))^2 propVarExpl, \qquad (31)
\end{aligned}$$

where $\theta^{(q)}$ is the angle between the vector $\boldsymbol{v}_1$ and the vector $\mathbf{1}$ whose components equal 1. This derivation assumed that all correlation in the reference as well as in the test networks are positive.

Approximate factorizability $\mathrm{cor}(x_i^{(q)},x_j^{(q)}) \approx kME_i\, kME_j$ (Eq.29) also implies that

$$\begin{aligned}
meanCor &\approx \frac{1}{(n^{(q)})^2}\sum_{i=1}^{n^{(q)}}\sum_{j=1}^{n^{(q)}} kME_i\, kME_j \qquad (32) \\
&= \left(\frac{\sum_i kME_i}{n^{(q)}}\right)^2 = (meanKME)^2 \ .
\end{aligned}$$

For an unsigned weighted network, $\mathrm{cor}(x_i^{(q)},x_j^{(q)}) \approx kME_i\, kME_j$ (Eq.29) implies that

$$meanAdj \approx \frac{1}{(n^{(q)})^2}\sum_{i=1}^{n^{(q)}}\sum_{j=1}^{n^{(q)}} |kME_i|^\beta |kME_j|^\beta = \left(\frac{\sum_i |kME_i|^\beta}{n^{(q)}}\right)^2$$

With Eq. 27, one can easily show that for $\beta = 2$

$$meanAdj \approx \left(\frac{\sum_i |kME_i|^2}{n^{(q)}}\right)^2 = propVarExpl^{[\mathrm{test}](q)} \ . \qquad (33)$$

Relationships among connectivity preservation statistics in correlation networks

If the eigennode factorizability of a module is high, one can show that [2]

$$\frac{kIM_i}{\sqrt{\sum_j kIM_j}} \approx |kME|_i^\beta \qquad (34)$$

for an unsigned weighted correlation network constructed with the soft threshold β (Eq. 9) [2].

For the correlation between intramodular connectivities

$$cor.kIM^{(q)} = \mathrm{cor}\left(kIM^{[\mathrm{ref}](q)}, kIM^{[\mathrm{test}](q)}\right) \ , \qquad (35)$$

this implies

$$cor.kIM^{(q)} \approx \mathrm{cor}\left(|kME^{[\mathrm{ref}](q)}|^\beta, |kME^{[\mathrm{test}](q)}|^\beta\right) \ . \qquad (36)$$

Thus, for the special case of a weighted module network with $\beta = 1$ and *positive* values of *kME*

$$cor.kIM \approx cor.kME\,. \tag{37}$$

The relationships between *cor.kME* and *cor.kMEall* depends on the extramodular nodes, i.e., the nodes outside the module network under consideration. Only if their contribution to the correlation *cor.kMEall* is negligible then

$$cor.kMEall \approx cor.kME\,. \tag{38}$$

Based on approximate factorizability (Eq. 29) one can show that

$$cor.cor^{(q)} \approx cor.kME^{(q)}\,, \tag{39}$$

where

$$cor.cor^{(q)} = \mathrm{cor}\left(vectorizeMatrix(r^{[\mathrm{ref}](q)}), vectorizeMatrix(r^{[\mathrm{test}](q)})\right)\,. \tag{40}$$

and

$$cor.kME^{(q)} = \mathrm{cor}_{i\in\mathcal{M}_q}(kME_i^{[\mathrm{ref}](q)}, kME_i^{[\mathrm{test}](q)})\,, \tag{41}$$

Similarly, approximate factorizability (Eq 29) of an unsigned weighted correlation network $a_{ij} = |\mathrm{cor}(x_i, x_j)|^\beta$ implies that

$$cor.Adj \approx \mathrm{cor}(|kME_i|^\beta, |kME_j|^\beta)\,. \tag{42}$$

Thus, in the special case where $\beta = 1$ and the module is comprised of genes with positive values of kME, *cor.Adj* is approximately equal to *cor.kME*.

Relationships among separability statistics in correlation networks

Here we derive a relationship between the average separability

$$separability.ave(q_1, q_2) = 1 - \frac{InterAdj.ave(q_1, q_2)}{IntraDensity(q_1, q_2)} \tag{43}$$

and an eigennode based analog for an unsigned weighted correlation network whose adjacency is given by $a_{ij} = |\mathrm{cor}(x_i, x_j)|^\beta$. If modules q_1 and q_2 have high eigennode factorizability (Eq. 28), one can show [2]

$$separability^{average}(q_1, q_2) \approx 1 - |\mathrm{cor}(E^{(q_1)}, E^{(q_2)})|^\beta. \tag{44}$$

Eq. 44 can be used to argue that by increasing the soft threshold β, correlation modules tend to become more separated. Further, note that for a weighted network with $\beta = 1$, we find that

$$separability.ave \approx separability.ME.$$

Preservation statistics in general factorizable module networks

Most of our results regarding the relationships between module preservation statistics critically depend on the approximate factorizability of the correlation matrix (Eq. 29), i.e., $cor(x_i^{(q)}, x_j^{(q)}) \approx kME_i\, kME_j$. We now outline a generalization of these derivations to general adjacency matrices that satisfy a related property: approximate factorizability. A network is referred to as approximately factorizable if its adjacency can be approximated as [1, 2]

$$a_{ij} \approx CF_i\, CF_j\,. \tag{45}$$

The quantity CF_i is called the *conformity* of node i. In this work we focus on networks in which individual module networks, rather than the whole network, are approximately factorizable. In this context, CF_i is called the *module conformity* of node i. Empirical evidence suggests that many module networks (in particular those defined as clusters) satisfy approximate factorizability [1]. For example, many module networks satisfy Equation 45 with

$$CF_i \approx \frac{kIM_i}{\sqrt{\sum_j kIM_j}}.$$

If the adjacency matrix A is approximately factorizable

$$a_{ij} \approx \frac{kIM_i \, kIM_j}{\sum_l kIM_l}$$

, then one can show that

$$cor.Adj \approx cor.kIM \,, \tag{46}$$

where

$$cor.Adj = \mathrm{cor}\left(vectorizeMatrix(A^{[\mathrm{ref}]}), vectorizeMatrix(A^{[\mathrm{test}]}) \right) . \tag{47}$$

In [2], it is shown that weighted correlation modules with high eigennode factorizability satisfy

$$a_{ij} = |\mathrm{cor}(x_i, x_j)|^{\beta} \approx CF_i^{(q)} \, CF_j^{(q)} \,, \tag{48}$$

where

$$CF_i \approx |kME_i|^{\beta} \tag{49}$$

For a general module network, the conformity CF_i can be interpreted as a generalization of the eigennode based connectivity. This suggests to define **conformity based preservation statistics** by replacing kME_i with CF_i in the definitions of

$$meanKME^{[\mathrm{test}](q)} \quad = \quad \mathrm{mean}_{i \in \mathcal{M}_q} \left\{ \mathrm{sign}(kME_i^{[\mathrm{ref}](q)}) kME_i^{[\mathrm{test}](q)} \right\} \tag{50}$$

and $cor.kME$ (Eq. 41).

7 Brief review of In-group proportion

Kapp and Tibshirani [3] have introduced a prediction-based method for measuring cluster preservation between a reference and a test data set. They define a cluster quality measure called the in-group proportion (IGP) and introduce a general procedure for individually validating clusters.

The method is formulated directly in terms of expression data. Let $X^{[\mathrm{ref}]}$ denote the $m \times n$ matrix of microarray data in the reference data, where m is the number of samples and n is the number of genes. Assume that a subset of the genes in X have been partitioned into Q clusters (labeled $1, 2, ..., Q$) and $C^{[\mathrm{ref}]}$ is the $m \times Q$ matrix of the centroids. Denote by $X^{[\mathrm{test}]}$ is an $p \times n$ matrix of microarray data independent of $X^{[\mathrm{ref}]}$, then all of the genes (columns) of $X^{[\mathrm{test}]}$ can be classified to one of the Q clusters or to a below-cutoff group using the centroids $C^{[\mathrm{ref}]}$ and a cutoff c. For gene i, define its cluster in the test data by

$$Cl_i^{[\mathrm{test}]} = \begin{cases} 0 & \text{if } \max\limits_{1 \le q \le Q} \mathrm{cor}(X_j^{[\mathrm{test}]}, C_q^{[\mathrm{test}]}) < c \\ \operatorname*{argmax}\limits_{1 \le q \le Q} \mathrm{cor}(X_j^{[\mathrm{test}]}, C_q^{[\mathrm{test}]}) & \text{if } \max\limits_{1 \le q \le Q} \mathrm{cor}(X_j^{[\mathrm{test}]}, C_q^{[\mathrm{test}]}) \ge c \end{cases} . \tag{51}$$

Thus, every gene in the test data is classified to the cluster represented by the nearest centroid, or to the below-cutoff group if the correlation of the gene with the nearest centroid is below the cutoff c. In this way, a new cluster assignment is obtained in the test data. The quality of these clusters is then evaluated using the IGP, defined as the proportion of genes in a cluster whose nearest neighbors are also in the same cluster. Denote by $nn(i)$ the nearest neighbor of gene i in the test data set, i.e., $nn(i) = \mathrm{argmax}\ _{k \neq i} \mathrm{cor}(X_k^{[\mathrm{test}]}, X_i^{[\mathrm{test}]}$. Then the IGP of cluster q is defined as

$$IGP(q, X^{[\mathrm{test}]}) = \frac{\#\{i | Cl_i^{[\mathrm{test}]} = Cl_{nn(i)}^{[\mathrm{test}]} = q\}}{\#\{i | Cl_i^{[\mathrm{test}]} = q\}} . \tag{52}$$

The authors of [3] compared the in-group proportion is compared to four other popular cluster quality measures (homogeneity score, separation score, silhouette width, and WADP score). They found the in-group proportion is the best measure of prediction accuracy. The algorithm is implemented in the package `clusterRepro` available from the Comprehensive R Archive Network (http://cran.r-project.org).

References

1. Dong J, Horvath S (2007) Understanding Network Concepts in Modules. BMC Systems Biology 1: 24.
2. Horvath S, Dong J (2008) Geometric interpretation of gene co-expression network analysis. PLoS Computational Biology 4.
3. Kapp AV, Tibshirani R (2007) Are clusters found in one dataset present in another dataset? Biostatistics 8: 9-31.
4. Tibshirani R, Walther G (2005) Cluster validation by prediction strength. Journal of Computational and Graphical Statistics 14: 511–528.
5. Yip A, Horvath S (2007) Gene network interconnectedness and the generalized topological overlap measure. BMC Bioinformatics 8: 22.
6. Ravasz E, Somera AL, Mongru DA, Oltvai ZN, Barabasi AL (2002) Hierarchical organization of modularity in metabolic networks. Science 297: 1551-5.
7. Zhang B, Horvath S (2005) General framework for weighted gene coexpression analysis. Statistical Applications in Genetics and Molecular Biology 4.
8. Li A, Horvath S (2007) Network neighborhood analysis with the multi-node topological overlap measure. Bioinformatics 23: 222-231.
9. Kaufman L, Rousseeuw P (1990) Finding Groups in Data: An Introduction to Cluster Analysis. New York: John Wiley and Sons, Inc.
10. Langfelder P, Zhang B, Horvath S (2007) Defining clusters from a hierarchical cluster tree: the Dynamic Tree Cut library for R. Bioinformatics 24: 719–20.
11. Carlson M, Zhang B, Fang Z, Mischel P, Horvath S, et al. (2006) Gene connectivity, function, and sequence conservation: Predictions from modular yeast co-expression networks. BMC Genomics 7: 40.
12. Horvath S, Zhang B, Carlson M, Lu K, Zhu S, et al. (2006) Analysis of oncogenic signaling networks in glioblastoma identifies aspm as a novel molecular target. PNAS 103: 17402–7.
13. Ghazalpour A, Doss S, Zhang B, Plaisier C, Wang S, et al. (2006) Integrating genetics and network analysis to characterize genes related to mouse weight. PloS Genetics 2: 8.
14. Gargalovic P, Imura M, Zhang B, Gharavi N, Clark M, et al. (2006) Identification of inflammatory gene modules based on variations of human endothelial cell responses to oxidized lipids. PNAS 103: 12741–6.
15. Oldham M, Horvath S, Geschwind D (2006) Conservation and evolution of gene coexpression networks in human and chimpanzee brains. PNAS 103: 17973-17978.
16. Langfelder P, Horvath S (2008) WGCNA: an R package for weighted correlation network analysis. BMC Bioinformatics 9: 559.
17. Mason M, Fan G, Plath K, Zhou Q, Horvath S (2009) Signed weighted gene co-expression network analysis of transcriptional regulation in murine embryonic stem cells. BMC Genomics 10: 327.
18. Oldham MC, Konopka G, Iwamoto K, Langfelder P, Kato T, et al. (2008) Functional organization of the transcriptome in human brain. Nature Neuroscience 11: 1271–1282.

Tables

Table 1. Overview of module quality statistics.

No.	Statistic	Statistic type	Network type	Reference netw. input		
				Label	Adj	*datX*
1	*meanAdj*	Density	general	yes	yes	no
2	$separability^{average}$	Separability	general	yes	yes	no
3	*meanCor*	Density	correlation	yes	no	yes
4	*propVarExpl*	Density	correlation	yes	no	yes
5	*meanKME*	Density	correlation	yes	no	yes
6	*separability*	Separability	correlation	yes	no	yes

The table reports the input needed for module quality measures that either measure module density or separability in the reference network. Note that the density based and separability based module quality measures (defined in the reference network) correspond to preservation statistics (evaluated in the test network).
