# Supplementary material for: Is My Network Module Preserved and Reproducible?
Source: PLoS Comput Biol. 2011 Jan 20;7(1):e1001057. doi: 10.1371/journal.pcbi.1001057 (PMC3024255; doi:10.1371/journal.pcbi.1001057)
Supplement: Text S2 — Details regarding module preservation between human and chimpanzee brain networks. In this document we provide detailed results regarding the preservation of human brain modules in chimpanzee brains. (0.22 MB PDF) [file pcbi.1001057.s007.pdf]

Supplementary text S2
Detailed results of Application 2: Preservation of co-expression modules between human and chimpanzee brains

Peter Langfelder, Rui Luo, Michael C. Oldham, and Steve Horvath*
*Corresponding author: shorvath@mednet.ucla.edu

In this document we provide detailed results of Application 2: Preservation of human brain modules in chimpanzee brains. For this study we used the expression data originally collected in [1] and whose network analysis was published in [2]. The data consist of 18 human and 18 chimpanzee samples collected from 6 matching brain regions (3 samples per region in each species). The human brain modules were identified in [2], whereas the chimpanzee brain modules were identified in this work using the same module detection parameters that were used for the human modules.

In Figure 1 we show the contingency tables of human modules vs. chimpanzee modules. A complete table of calculated module preservation statistics can be found in the accompanying Supplementary Table S1. This is a flat comma separated value (CSV) text file that can be viewed in most standard spreadsheet software such as MS Excel and OpenOffice Calc. The columns indicate the reference set, test set, module, module size, observed preservation statistics, and their Z scores.

In Figures 2 and 3 we present the Z scores of quality statistics graphically, while in Figures 4 and 5 we present the Z scores of preservation statistics. In each figure, we plot the module quality and preservation Z scores (y-axis) as a function of module size (x-axis). The reference and test data sets are indicated in the title of each plot. Modules are labeled by their color and numeric labels (turquoise module also carries the numeric label 1, blue 2, brown 3, yellow 4, green 5, red 6, black 7). The dashed blue and green lines indicates the thresholds $Z = 2$ and $Z = 10$, respectively.

References

1. Khaitovich P, Muetzel B, She X, Lachmann M, Hellmann I, et al. (2004) Regional Patterns of Gene Expression in Human and Chimpanzee Brains. Genome Res 14: 1462-1473.

2. Oldham M, Horvath S, Geschwind D (2006) Conservation and evolution of gene coexpression networks in human and chimpanzee brains. PNAS 103: 17973-17978.

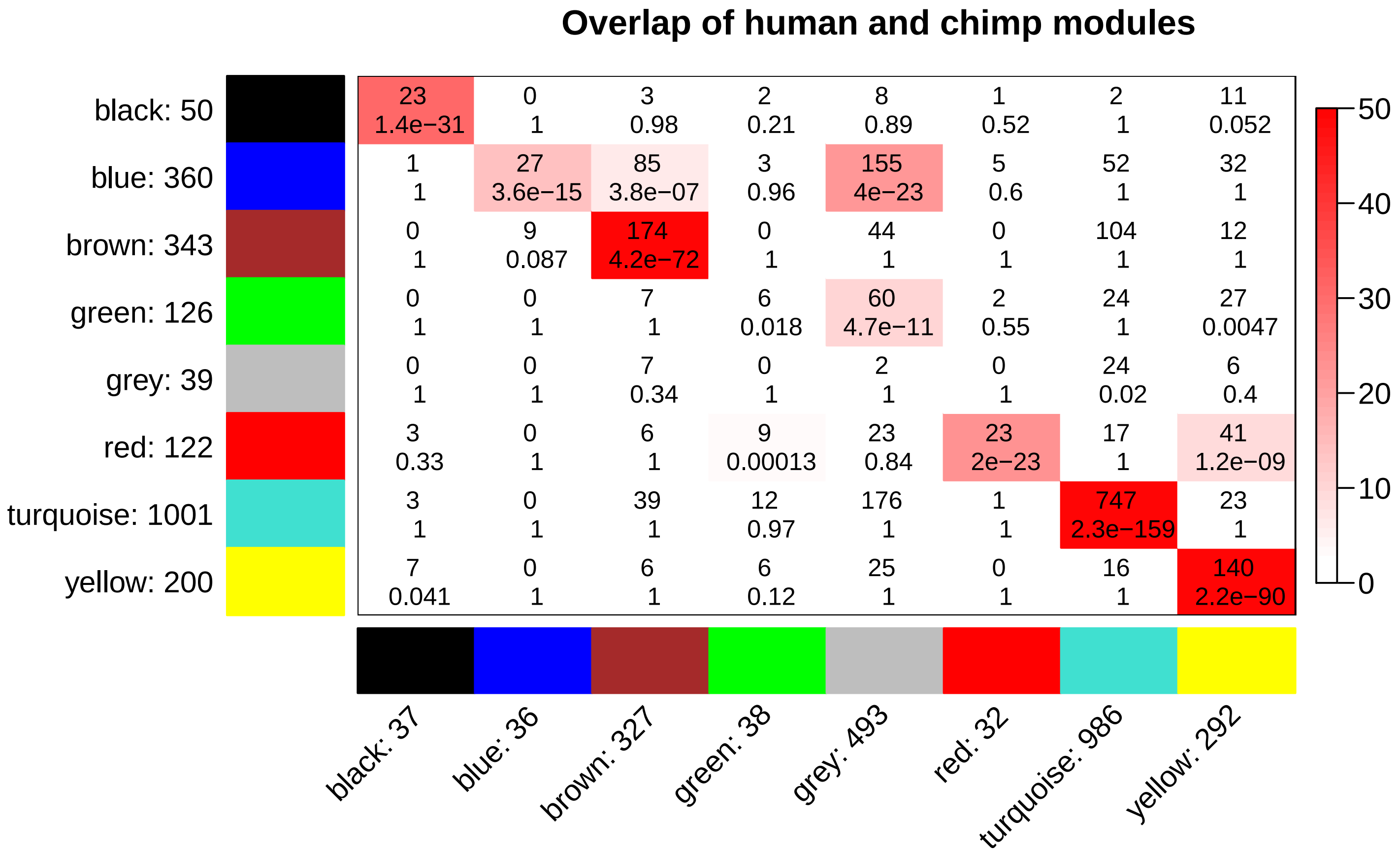

Figure 1. Overlap of human modules identified in [2] with chimpanzee modules identified using the same module detection settings. Each row corresponds to a human brain module, and each column corresponds to a chimpanzee brain module. Each module is labeled by its color label; we also indicate the total number of genes in the module. Within the each cell, the top number indicates the number of genes in the overlap of the corresponding row and column modules, while the bottom number indicates the significance of the overlap, quantified by Fisher's exact test. The significance is also indicated by the color of each cell. Darker red indicates more significant overlap; the color legend is based on $-log_{10}(p)$.

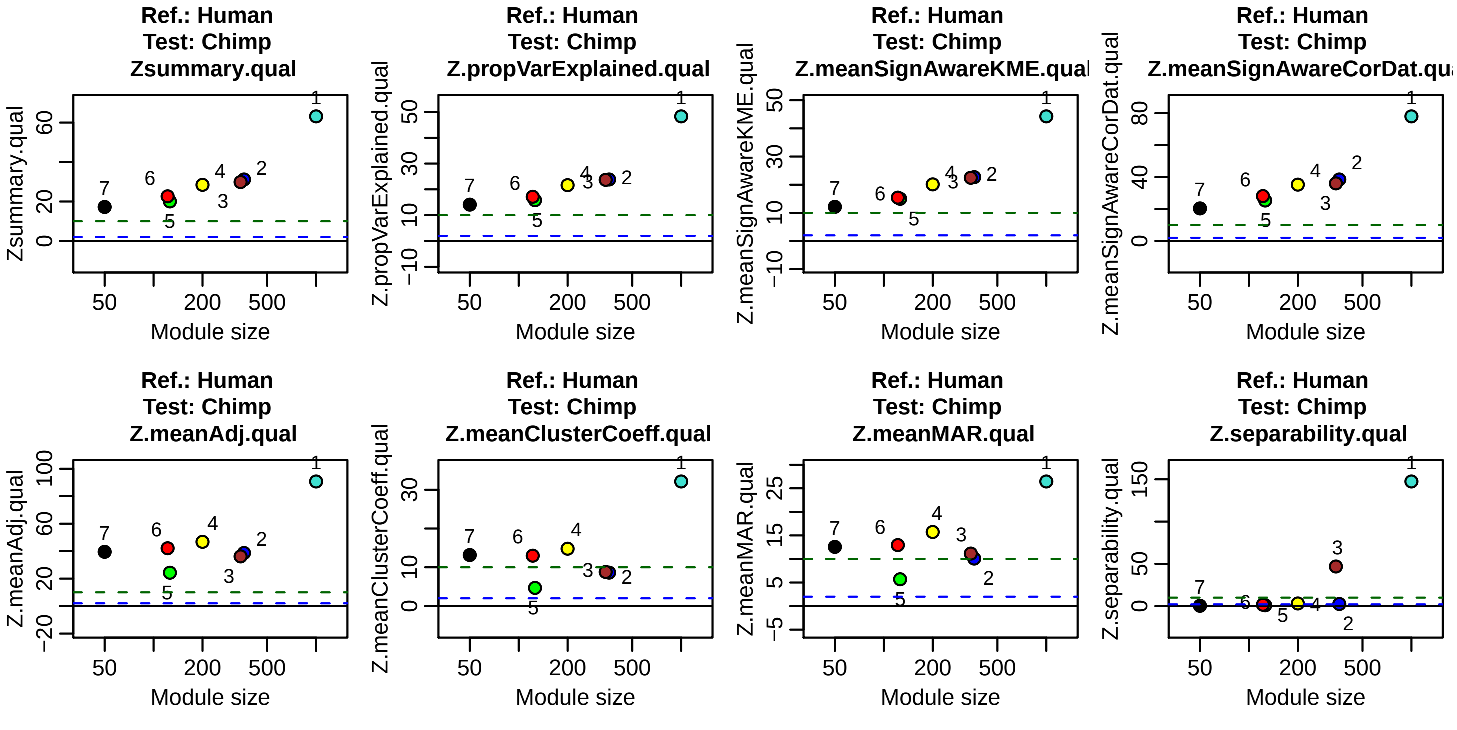

Figure 2. Z scores of quality of human brain modules.

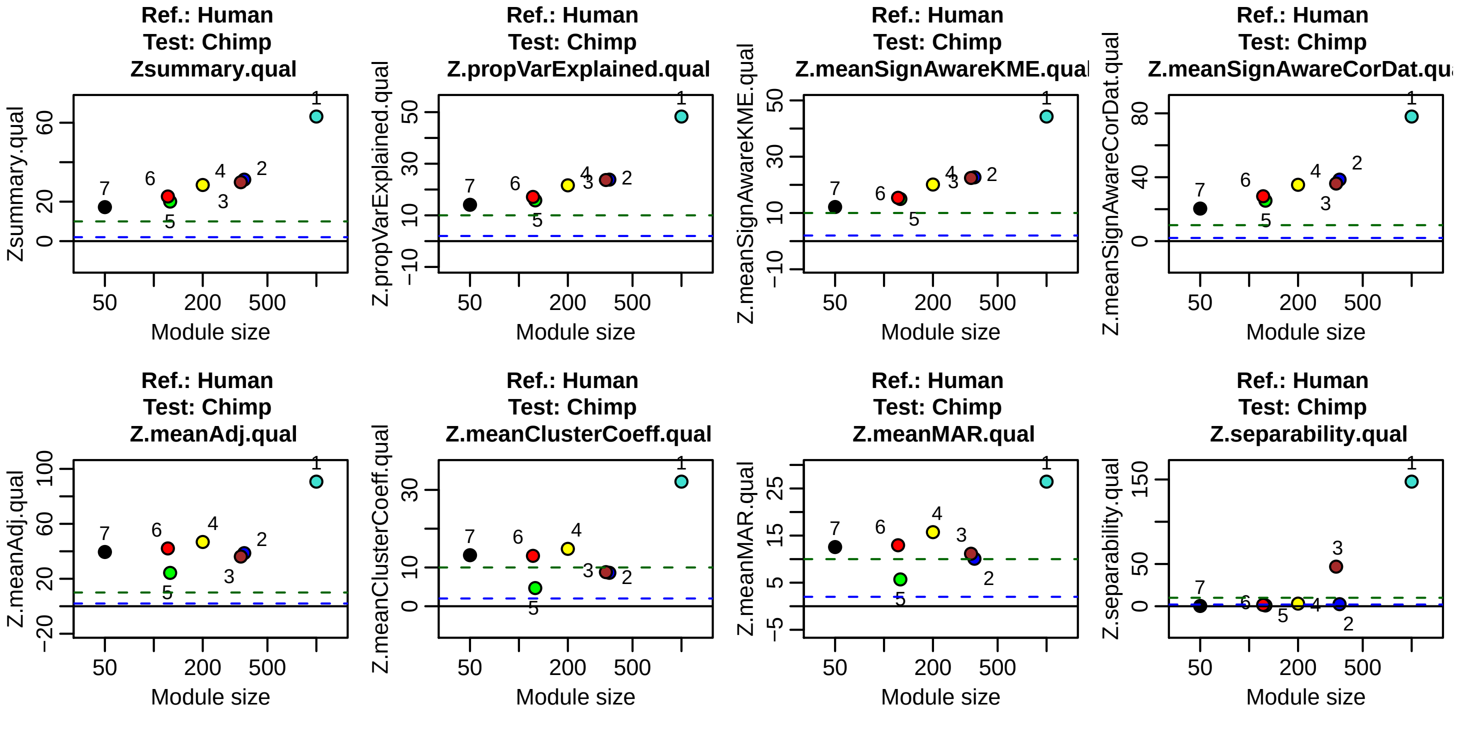

Figure 3. Z scores of quality of chimpanzee brain modules.

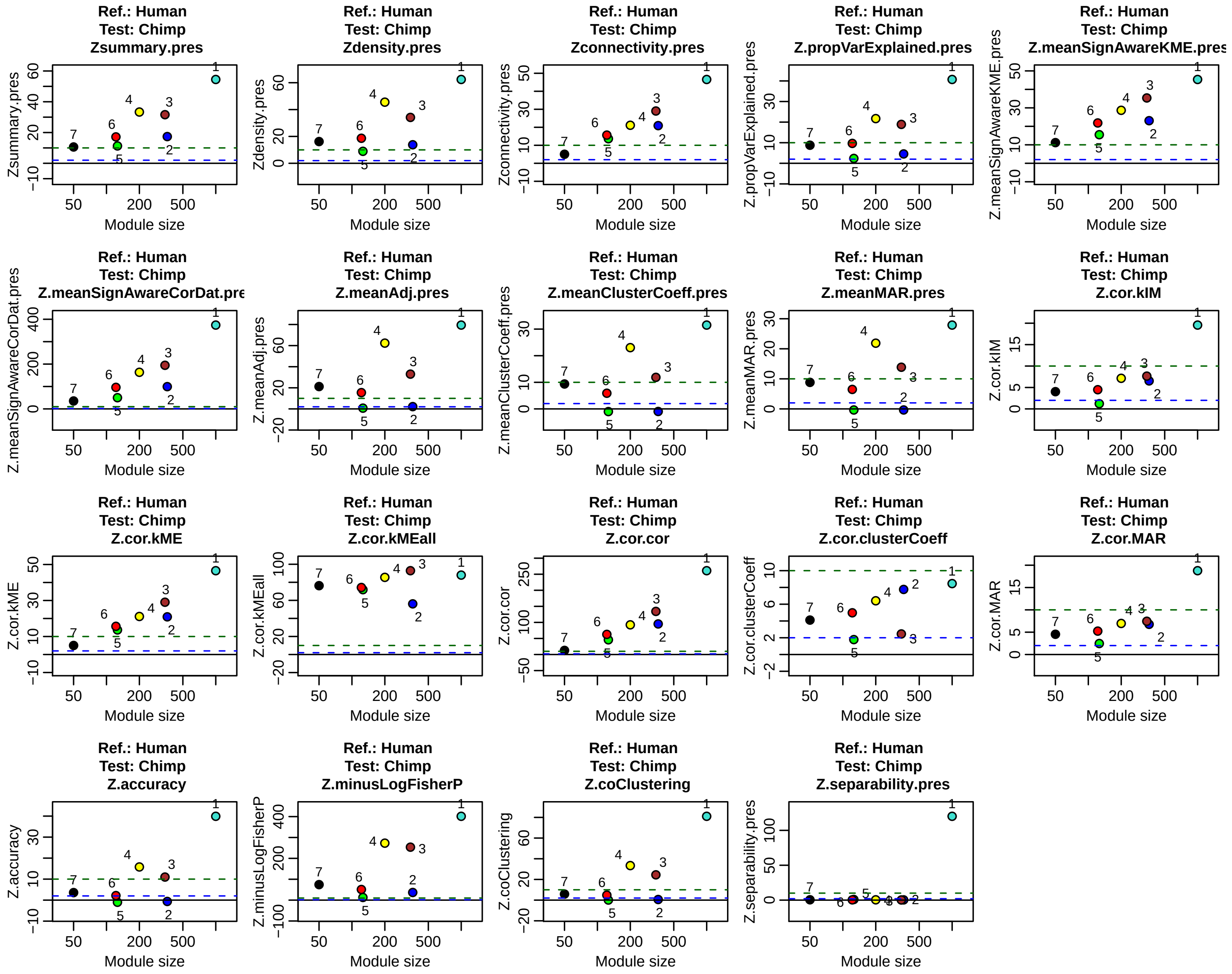

Figure 4. *Z* scores of preservation of human brain modules in chimpanzee brains.

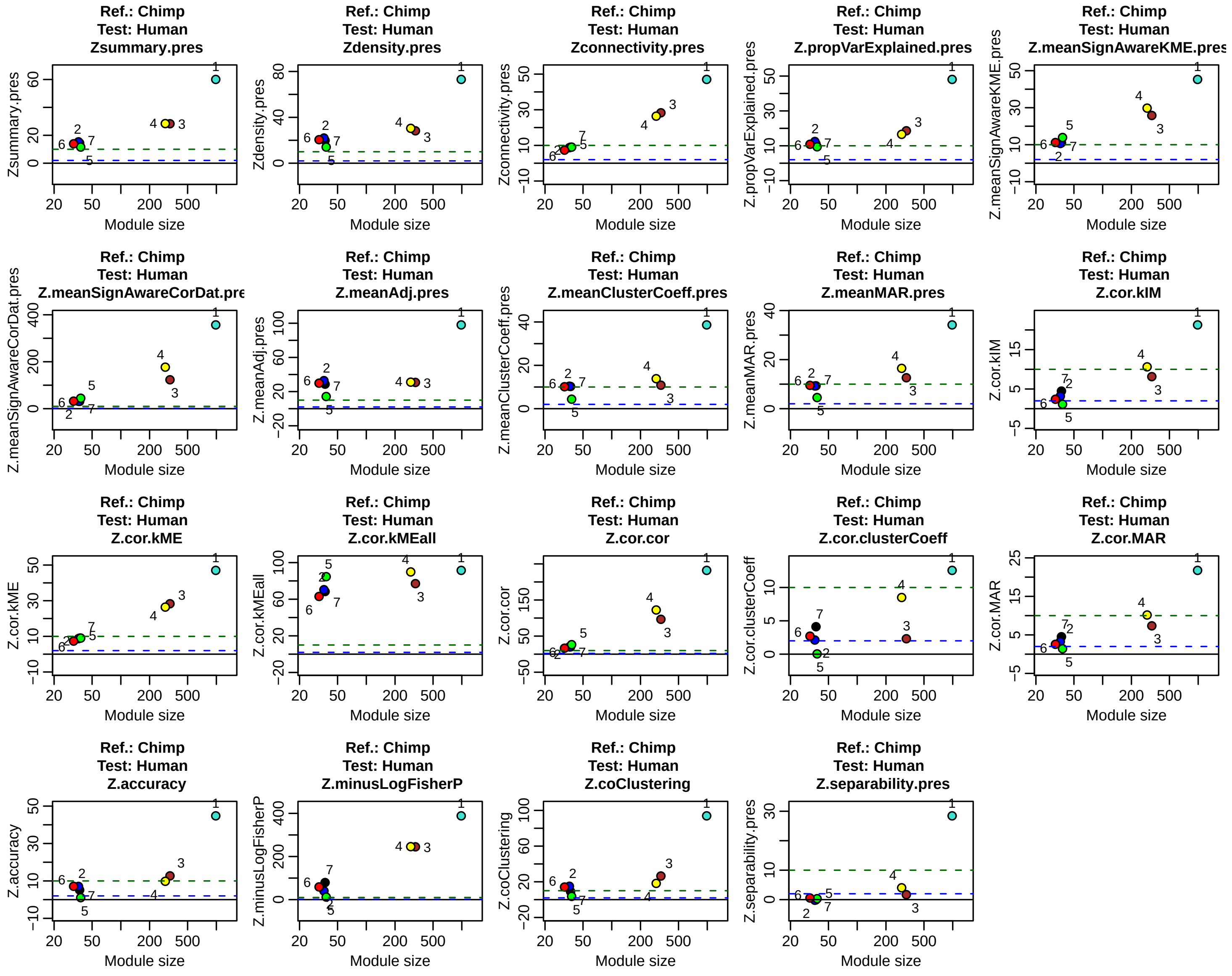

Figure 5. Z scores of preservation of chimpanzee brain modules in human brains.
