# Supplementary material for: Is My Network Module Preserved and Reproducible?
Source: PLoS Comput Biol. 2011 Jan 20;7(1):e1001057. doi: 10.1371/journal.pcbi.1001057 (PMC3024255; doi:10.1371/journal.pcbi.1001057)
Supplement: Text S3 — Detailed description of the human brain. In this document we provide detailed results of Application 4: Preservation of cortical modules between male and female samples. (2.51 MB PDF) [file pcbi.1001057.s008.pdf]

Supplementary text S3
Detailed results of Application 4: Preservation of cortical modules between male and female samples

Peter Langfelder, Rui Luo, Michael C. Oldham, and Steve Horvath*
*Corresponding author: shorvath@mednet.ucla.edu

In this document we provide detailed results of Application 4: Preservation of cortical modules between male and female samples.

It is not known whether the large-scale transcriptional organization in human cortex is the same in men and women. To answer one aspect of this question, we studied whether gene co-expression modules found in networks based on male cortical samples are preserved in networks based on female cortical samples. We used microarray gene expression data from a recent publication [1] in which we constructed coexpression networks and modules in different human brain regions. In [1] we found that many of the resulting co-expression modules are biologically meaningful, for example some are enriched with cell type specific genes for major cell classes (neurons, oligodendrocytes, astrocytes and microglia). In our original analysis we disregarded sex differences and analyzed brain samples from both men and women together.

To identify sex-specific modules one must ensure that the modules tested for preservation are robust since modules may arise due to technical artifacts and array outliers. To counter the concern of technical artefacts, it is useful to show that co-expression modules are microrray platform independent, i.e., that they are preserved between data sets generated with different microarray chips. Fortunately, we had two independent data sets of cortical samples from [1]: the first data set (labelled CTX) used Affymetrix HGU133a microarrays and contains 48 male and 19 female samples, and the second data set (labelled CTX95) used Affymetrix HGU95av2 microarrays and contains 31 male and 11 female samples. Using a cross-tabulation based approach, we found that modules identified in the CTX network are highly preserved in the CTX95 network (and further in a network constructed from Illumina microarrays [1]). Given this high extent of module preservation between the CTX and the CTX95 data, we constructed a "consensus" network based on male CTX and CTX95 samples [2] whose modules are referred to as consensus modules. Roughly speaking, the consensus modules are comprised of genes that are tightly co-expressed in both male samples from the CTX and CTX95 data sets. By construction, consensus modules across the CTX and the CTX95 data should exhibit a high preservation between the two networks. Our results discussed below show that this is indeed the case.

The male consensus module construction identified 14 modules in the male network comprising a total of 1735 genes, while 6289 genes were not assigned to a module. The clustering dendrogram and module assignment is shown in Figure 1A. The female CTX network analysis identified 16 modules containing a total of 2770 genes. The 14 male and 16 female modules show strong overlap with the modules found in the original analysis [1] that combined both sexes. The corresponding overlap tables are shown in Figures 3 and 4. The contingency table of male and female modules in Figure 1B shows that there is relatively high agreement between the male and female module assignments. However, there are 3 male modules (labelled 6, 20, 23) that, based on the table, appear to be not preserved in females since most of their genes are classified as unassigned (label 0 or grey color) in the female network. The p-values of their overlaps with other modules are not significant (the best p-value is, after Bonferroni correction, 0.05). Were one restricted to cross-tabulation measures, one would conclude that the male modules 6, 20, 23 are absent in the female cortical network. However, network module preservation statistics suggest that the modules are in fact preserved in the female network, and could perhaps be found

using a different module identification. For example, for the male module 6 we find high density preservation statistics $Z_{meanAdj} = 11$ ($p_{meanAdj} = 1.5 \times 10^{-25}$), $Z_{propVarExpl} = 4.7$ ($p_{propVarExpl} = 1.1 \times 10^{-6}$), $Z_{meanKME} = 13$ ($p_{meanKME} = 6.4 \times 10^{-39}$), $Z_{meanCor} = 31$ ($p_{meanCor} < 10^{-200}$), and overall somewhat lower, but still significant connectivity preservation statistics $Z_{cor.cor} = 12$ ($p_{cor.cor} = 5 \times 10^{-32}$), $Z_{cor.kIM} = 4.2$ ($p_{cor.kIM} = 1.2 \times 10^{-5}$), $Z_{cor.kME} = 3.0$ ($p_{cor.kME} = 0.0015$), $Z_{cor.kMEall} = 41$ ($p_{cor.kMEall} < 10^{-200}$). Scatter plots of intramodular correlations (Figure 1C), intramodular connectivities kIM (Figure 1D), and eigengene-based connectivities (Figure 1E) provide a visual confirmation that connectivity patterns in male module 6 are indeed preserved in the female CTX network. The composite preservation statistic is $Z_{summary} = 8$ ($p_{summary} = 3.4 \times 10^{-19}$), indicating a moderate to strong evidence for module preservation. The preservation statistics for male modules 20 and 23 are qualitatively similar, with summary statististics $Z_{summary} = 5.4$ ($p_{summary} = 4.5 \times 10^{-10}$) for module 20 and $Z_{summary} = 6.2$ ($p_{summary} = 2.8 \times 10^{-12}$) for module 23. A table summarizing the values of all preservation statistics is provided in Supplementary Table S2.

Recall that the modules 6, 20, 23 do not have corresponding modules in the female CTX network. However, overall the network preservation statistics for the modules 6, 20, and 23 suggest that the modules are in fact preserved between the male and female samples, and their apparent absence in female data can be attributed to the particular choice of module identification parameters. This application illustrates that cross-tabulation based preservation statistics can be inferior to network based preservation statistics when it comes to making statements about module non-preservation.

The composite preservation statistic $Z_{summary}$ provides a convenient summary of the findings described above. Figure 2 shows $Z_{summary}$ for with the male CTX set as reference, and all other data sets as test sets. Panel A indicates that consensus modules across the male CTX and CTX95 data sets are preserved in the two data sets, which is expected given the consensus module construction method. Panels B and C present the preservation of male consensus modules in the female CTX and CTX95 data sets. These panels address our original biological question: do male cortical networks exhibit strong sex-specific modules, that is modules strongly non-preserved in the female samples? The answer is negative: all male consensus modules show at least weak to moderate evidence of preservation ($Z_{summary} > 2$) in the female network. Conversely, we have also studied the preservation of female modules in the male network. But since far fewer female array samples were available, module detection in the female network is less reliable and we relegate the corresponding results to Supplement Supplementary text S3.

Overall, we find no evidence for sex specific co-expression modules in human cortical co-expression networks. Limitations of our study include the low sample size and the focus on cortical tissue. In other tissues, there may be more pronounced differences, e.g., stronger evidence of co-expression module differences were found in mouse liver tissues [3]. We only studied relatively large co-expression modules whose biological function is described in [1]. Many of these human brain modules correspond to cell types and it would be very surprising to find a strong sex effect. In mouse brain, co-expression modules were also found to be roughly preserved across sex which is congruent with our findings [3]. It is beyond our scope to investigate differences in mean gene expression levels between the sexes but we refer the interested reader to several microarray papers that report lists of genes that are differentially expressed between men and women in whole brain or part of brain [4–6]. Most human studies are poorly powered, which may explain why relatively few genes have been identified. A recent relatively large study involving mice yielded much larger estimates of the percentage of sexually dimorphic genes (70% in mouse liver but only 15% in brain) but the mean size of the sex difference in level of expression was only about 8% [3]. Only a small number of genes reached 2 fold higher expression in one sex in brain or any tissue disregarding gonads, since they are different tissues in males and females [7].

Comparison to other methods

As mentioned above, cross-tabulation methods suggest that modules 6, 20, and 23 are not preserved between the male and female samples. Separability statistics suggest module 6 is separated from other modules ($Z_{separability} = 4.8$) but modules 20 and 23 are not ($Z_{separability} = 0.5$ and -0.5, respectively). The In-Group Proportion [8] permutation p-values are shown in Figure 2, panels D–F, for the three tests sets Male CTX95,

female CTX, and female CTX95. Although the IGP permutation p-values suggests results broadly similar to those found by network preservation statistics, there are some discrepancies. For example, IGP suggests that the consensus module 23 is in fact very weakly preserved in the male CTX95 data (permutation p-value $p = 0.045$). The corresponding network preservation statistic is $Z_{summary} = 9.4$ ($p = 2.6 \times 10^{-38}$), indicating fairly strong preservation. Similarly, IGP indicates that module 19 is very weakly preserved in the female CTX data (permutation p-value $p = 0.049$), while the network preservation summary statistic is $Z_{summary} = 6.4$ ($p = 3.7 \times 10^{-14}$), indicating moderate to strong preservation. Module 19 is associated with the gene ontology terms Membrane/signal transduction with p-value 7×10^{-8} [1]. Since this is a basic biological function that is common to males and females, it is reasonable to expect that this module should be preserved between male and female samples. For module 5, the IGP permutation p-value in the female CTX data is $p = 0.038$, also indicating very weak preservation. This module is highly enriched in immune response genes (enrichment p-value 10^{-41}) [1], and both cross-tabulation and network preservation statistics suggest this module is preserved. Since the separability of this module is relatively low, it is likely that the significant permutation p-value obtained by IGP reflects that IGP combines aspects of density and separability.

Plots of selected Z statistics

A complete table of results can be found in the accompanying Supplementary Table S2. This is a flat comma separated value (CSV) text file that can be viewed in most standard spreadsheet software such as MS Excel and OpenOffice Calc. The columns indicate the reference set, test set, module, module size, observed preservation statistics, and their Z scores.

In Figures 6–14 we present the Z scores of quality statistics, while Figures 15–23 show the Z scores of preservation statistics. We note that although module quality in principle does not depend on the test network, in our implementation the module quality is only measured on genes that are also present in the test network. This makes the quality measures comparable the corresponding preservation measures. However, this also means that module quality statistics in a given reference network vary with the test set.

In each figure, we plot the module quality and preservation Z scores (y-axis) as a function of module size (x-axis). The reference and test data sets are indicated in the title of each plot. Modules are labeled by their numeric labels. Modules that could be matched (using the function `matchLabels` in the WGCNA R package) to the CTX modules presented in Oldham *et al* [1] are also labeled by the color of the corresponding CTX module in Oldham *et al.* The dashed blue and green lines indicate the thresholds $Z = 2$ and $Z = 10$, respectively.

References

1. Oldham MC, Konopka G, Iwamoto K, Langfelder P, Kato T, et al. (2008) Functional organization of the transcriptome in human brain. Nature Neuroscience 11: 1271–1282.

2. Langfelder P, Horvath S (2007) Eigengene networks for studying the relationships between co-expression modules. BMC Systems Biology 1: 54.

3. van Nas A, GuhaThakurta D, Wang SS, Yehya N, Horvath S, et al. (2009) Elucidating the Role of Gonadal Hormones in Sexually Dimorphic Gene Coexpression Networks. Endocrinology 150: 1235-1249.

4. Vawter MP, Evans S, Choudary P, Tomita H, Meador-Woodruff J, et al. (2004) Gender-Specific Gene Expression in Post-Mortem Human Brain: Localization to Sex Chromosomes. Neuropsychopharmacology 29: 373–384.

5. Reinius B, Saetre P, Leonard JA, Blekhman R, Merino-Martinez R, et al. (2008) An evolutionarily conserved sexual signature in the primate brain. PLoS Genet 4: e1000100.

6. Simunovic F, Yi M, Wang Y, Stephens R, Sonntag KC (2010) Evidence for gender-specific transcriptional profiles of nigral dopamine neurons in parkinson disease. PLoS ONE 5: e8856.

7. Arnold AP, van Nas A, Lusis AJ (2009) Systems biology asks new questions about sex differences. Trends in Endocrinology Metabolism 20: 471 - 476.

8. Kapp AV, Tibshirani R (2007) Are clusters found in one dataset present in another dataset? Biostat 8: 9-31.

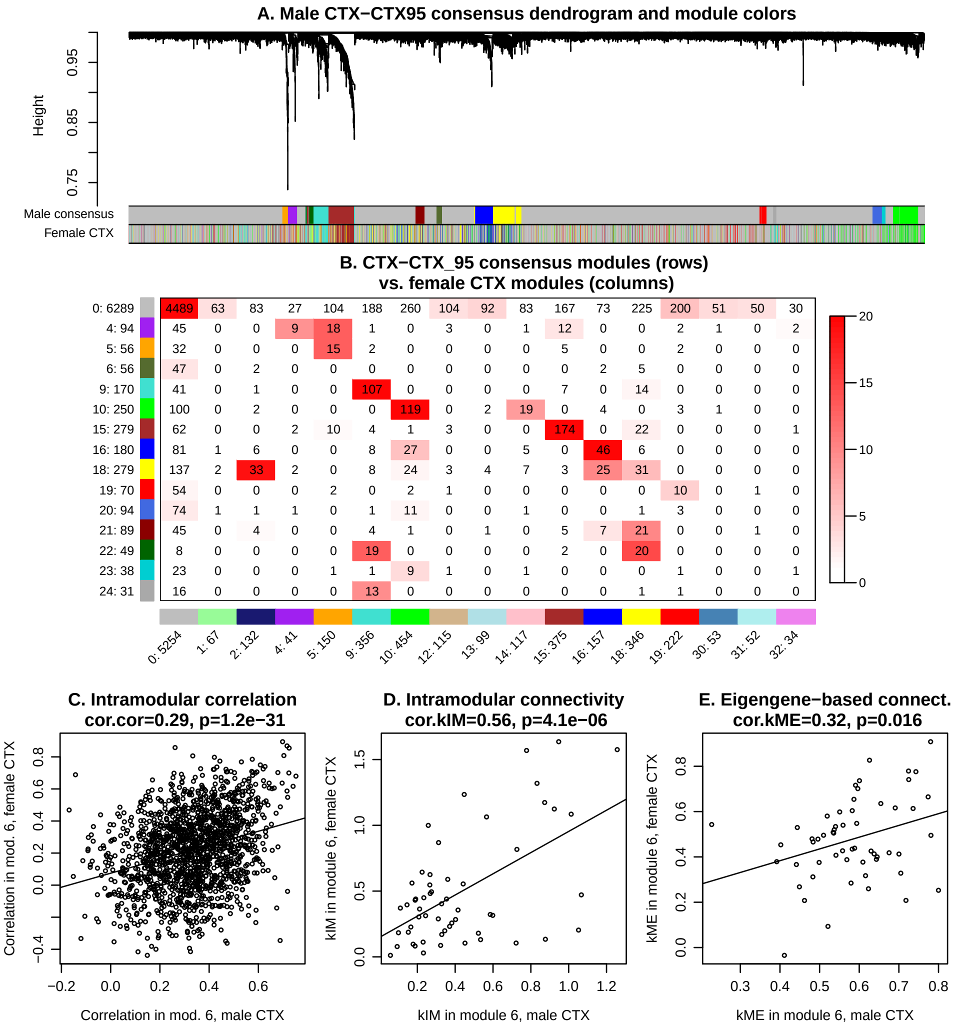

Figure 1. Network analysis in male and female cortical samples and an example of preserved modules. A. Hierarchical clustering tree (dendrogram) based on consensus gene dissimilarity across male CTX and CTX95 samples. Each "leaf" (short vertical line) corresponds to one gene. The color rows below the dendrogram indicate module membership in the consensus modules (defined by cutting branches of this dendrogram) and in the female CTX modules (defined by branch cutting of an analogous dendrogram based on the female CTX samples). The color rows show that some male consensus and female modules overlap (for example, the brown module), but other appear to be sex-specific. B. Cross-tabulation of male consensus modules (rows) and female CTX modules (columns). Each row and column is labelled by the corresponding numeric module label, the total number of genes in the module, and the module color. In the table, numbers give counts of genes in the intersection of the corresponding row and column module. The table is color-coded by $-log(p)$, the Fisher exact test p value, according to the color legend on the right. C. Scatter plot of gene-gene correlations based on the male CTX samples (x-axis) and the female CTX samples (y-axis) within male consensus module 6. Each point corresponds to a gene-gene pair. The scatter plot exhibits a significant correlation (cor.cor and p-value displayed in the title), indicating that the connectivity of male consensus module 6 is preserved in the female CTX samples. D. Scatter plot of intramodular connectivities of genes in male consensus module 6, in the male CTX samples (x-axis) and in the female CTX samples (y-axis). Each point corresponds to one gene. The scatter plot exhibits a significant correlation (cor.kIM and p-value displayed in the title), indicating that the hub gene status in male consensus module 6 is preserved in the female CTX samples. E. Scatter plot of eigengene-based connectivities of genes in male consensus module 6, in the male CTX samples (x-axis) and in the female CTX samples (y-axis). Each point corresponds to one gene. The scatter plot exhibits a significant correlation (cor.kME and p-value displayed in the title), indicating that the hub gene status in male consensus module 6 is preserved in the female CTX samples.

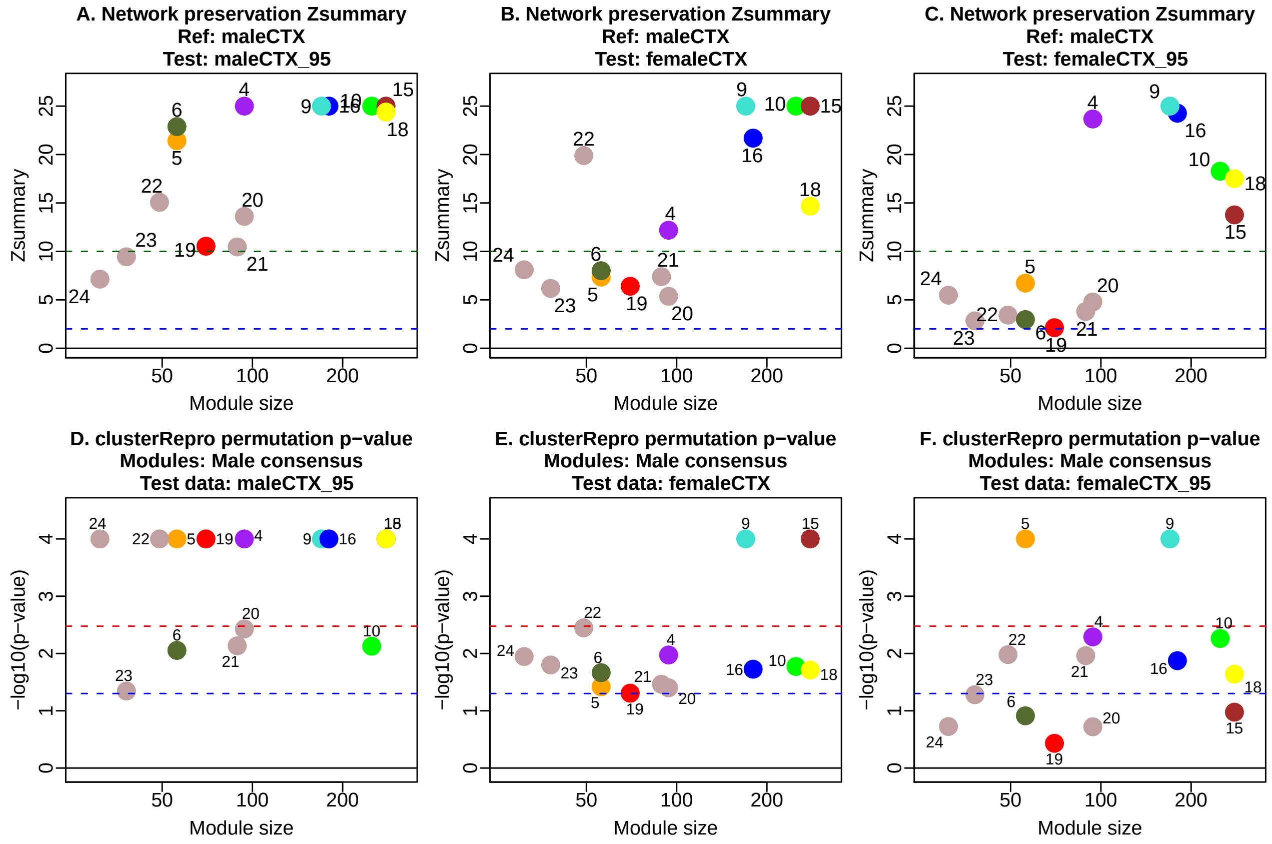

Figure 2. Preservation of modules between male and female cortical networks and comparison to IGP. Panels A–C present the summary preservation statistics $Z_{summary}$ for module preservation (y-axis), as a function of the module size (x-axis). The dashed blue and green lines indicate the thresholds $Z_{summary} = 2$ and 10, respectively. We used the male CTX data set as the reference; the corresponding test set is indicated in the title of each plot. Panels D–F present the IGP permutation p-values in the same test data sets. The dashed blue and red lines represent thresholds $p = 0.05$ and 0.0033 (Bonferroni correction for the number of modules). The male modules were identified using a consensus module analysis of the CTX and CTX95 data sets. This is reflected in the high preservation statistics in panels A and D. Overall, the male consensus modules are preserved in the female data, indicating that this analysis does not find strong sex-specific modules. Each module is labelled by its numeric label. If a module can be matched to one of Oldham *et al*'s modules [1], the circle is colored by the corresponding color in Oldham *et al.*

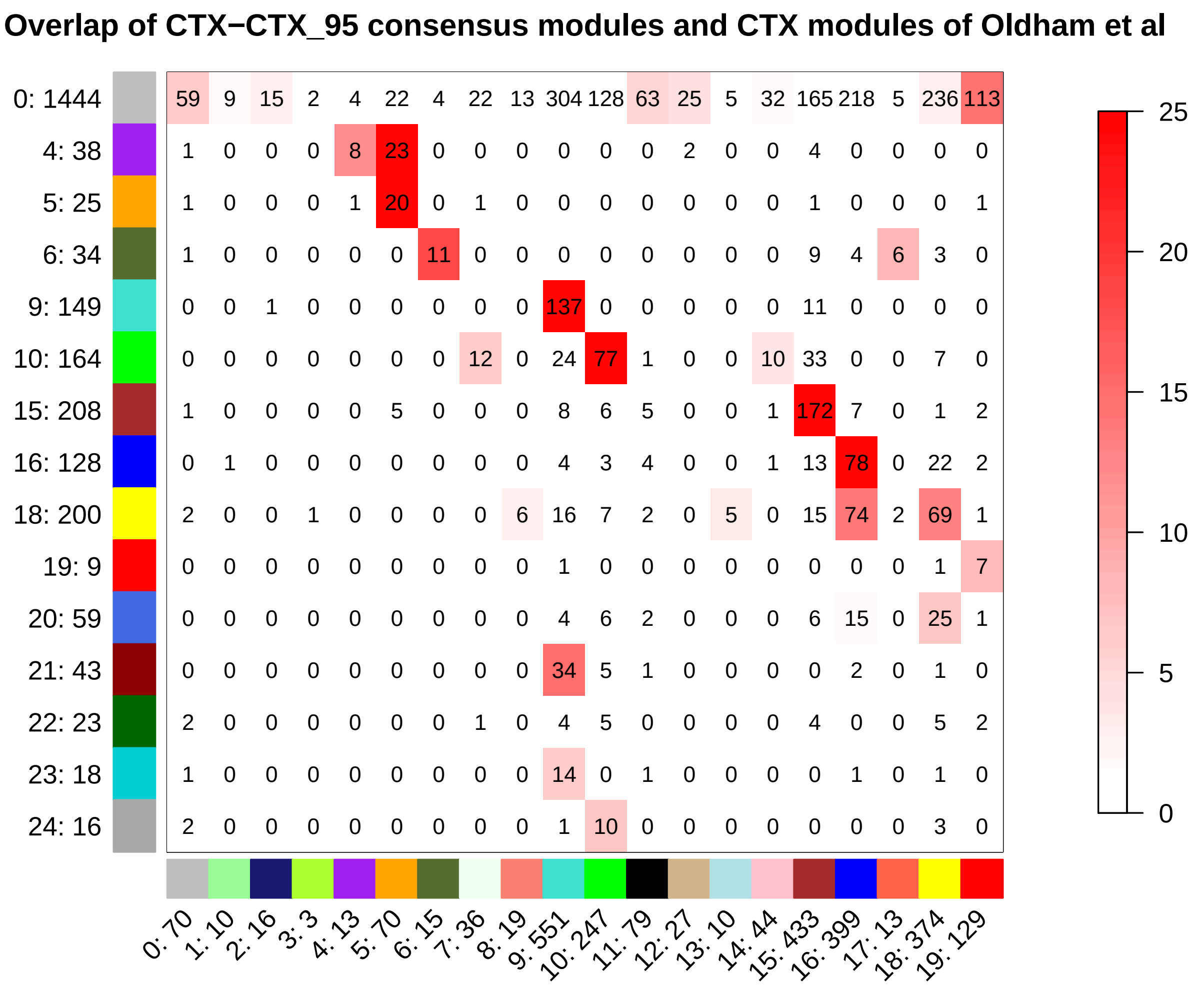

Figure 3. Overlap of male CTX–CTX95 consensus modules with the CTX modules in Oldham *et al* [1]. Each row corresponds to a male CTX–CTX95 consensus module, and each column corresponds to a CTX module presented in Oldham *et al.* Each module is labeled by its numeric and color label; we also indicate the total number of genes in the module. Because we have collapsed Oldham *et al*'s probeset-level expression data to gene-level, Oldham *et al*'s modules in this comparison are smaller than those published in [1]. Within the table, numbers indicate the number of genes in the overlap of the corresponding row and column modules. The significance of the overlap, quantified by Fisher's exact test, is indicated by the color. Darker red indicates more significant overlap; the color legend is based on $-log_{10}(p)$. The table shows that many of the consensus modules can be matched to their corresponding CTX modules in Oldham *et al.*

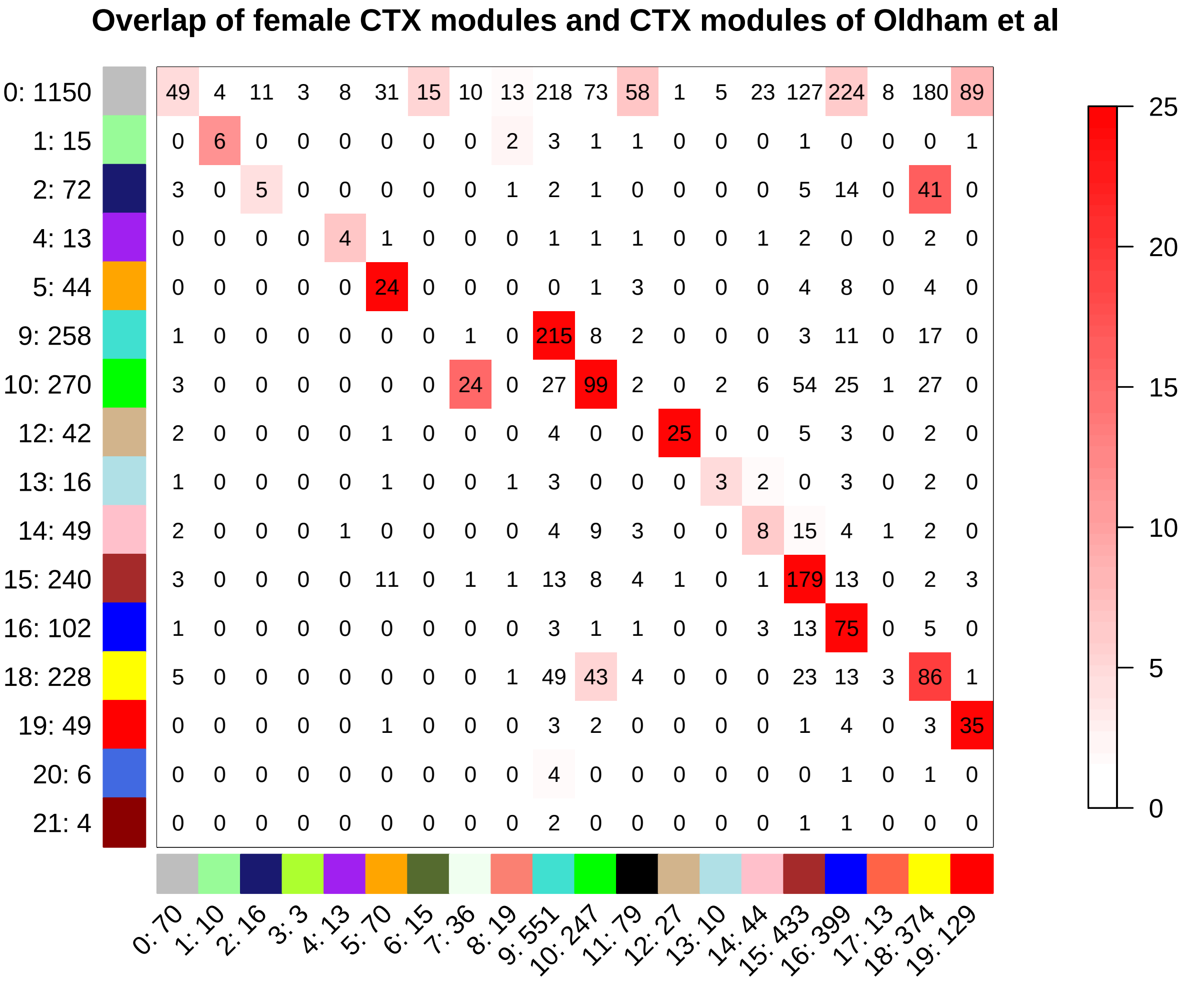

Figure 4. Overlap of female CTX modules with the CTX modules in Oldham *et al* [1]. Each row corresponds to a female CTX module, and each column corresponds to a CTX module presented in Oldham *et al.* Each module is labeled by its numeric and color label; we also indicate the total number of genes in the module. Because we have collapsed Oldham *et al*'s probeset-level expression data to gene-level, Oldham *et al*'s modules in this comparison are smaller than those published in [1]. Within the table, numbers indicate the number of genes in the overlap of the corresponding row and column modules. The significance of the overlap, quantified by Fisher's exact test, is indicated by the color. Darker red indicates more significant overlap; the color legend is based on $-log_{10}(p)$. The table shows that many of the female CTX modules can be matched to their corresponding CTX modules in Oldham *et al.*

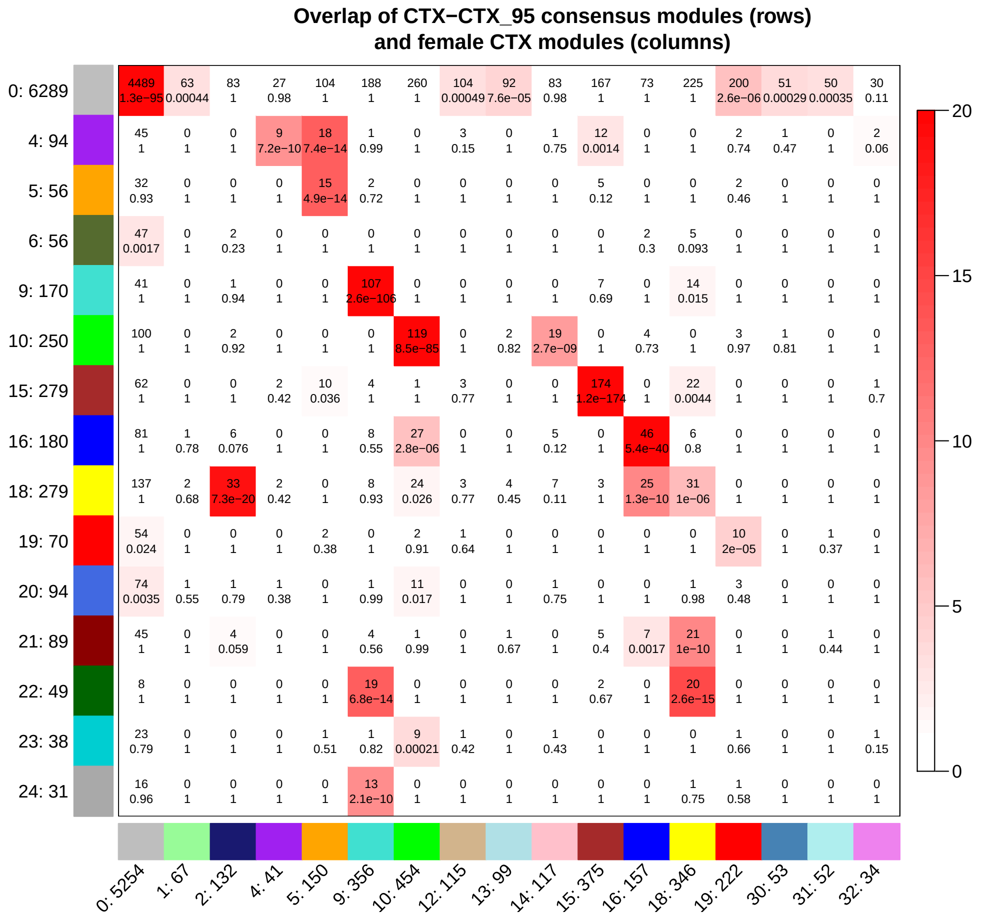

Figure 5. Overlap of female CTX modules with the male CTX–CTX95 consensus modules. Each row corresponds to a male CTX–CTX95 consensus module, and each column corresponds to a female CTX module. Each module is labeled by its numeric and color label; we also indicate the total number of genes in the module. Within the table, numbers indicate the number of genes in the overlap of the corresponding row and column modules and the Fisher exact test p-value. The Fisher test p-value is also indicated by the color. Darker red indicates more significant overlap; the color legend is based on $-log_{10}(p)$. The table shows that most male CTX–CTX95 modules have a female counterpart with which they have a very significant overlap. However, modules 6, 20, and 23 do not have a significant female counterpart.

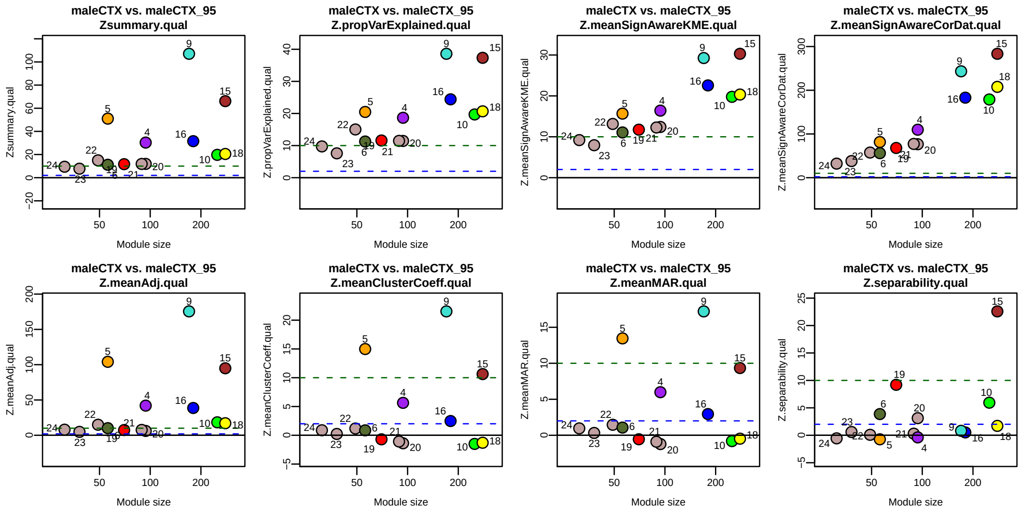

Figure 6. Module quality in the male CTX reference data set with the male CTX95 test set.

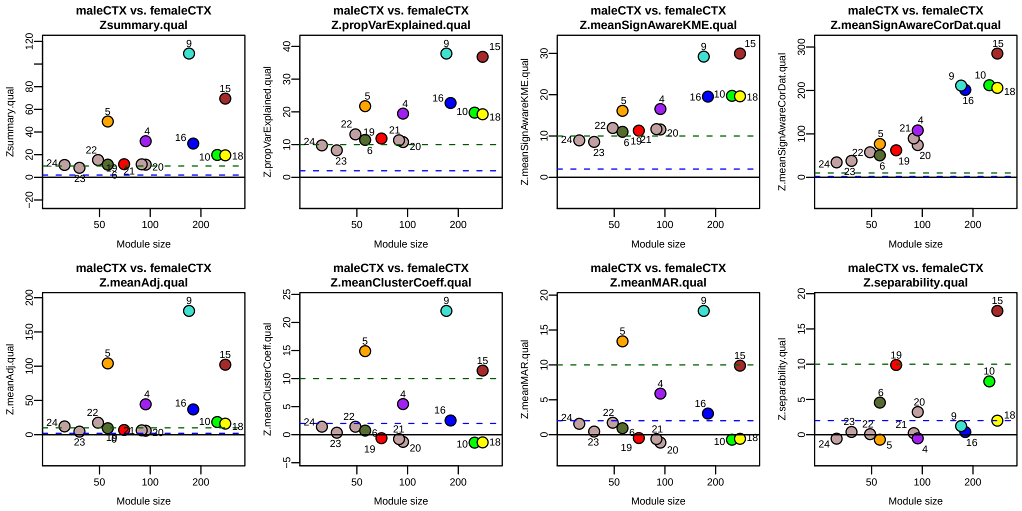

Figure 7. Module quality in the male CTX reference data set with the female CTX test set.

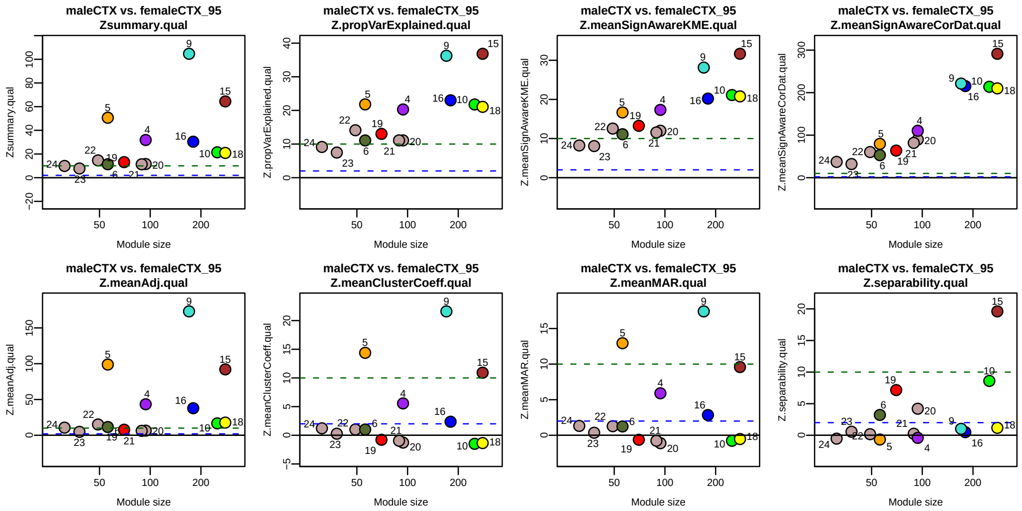

Figure 8. Module quality in the male CTX reference data set with the female CTX95 set as the test network.

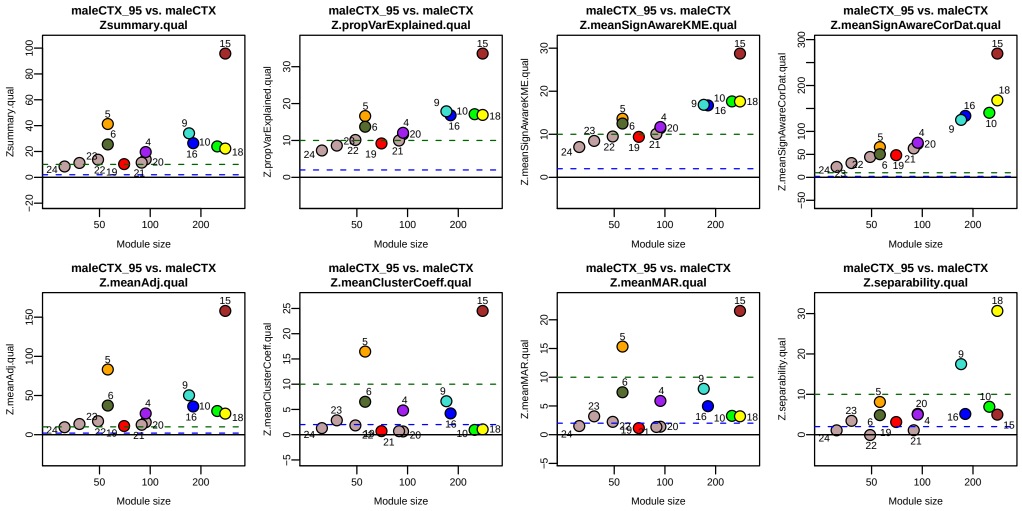

Figure 9. Module quality in the male CTX95 reference data set with the male CTX set as the test network.

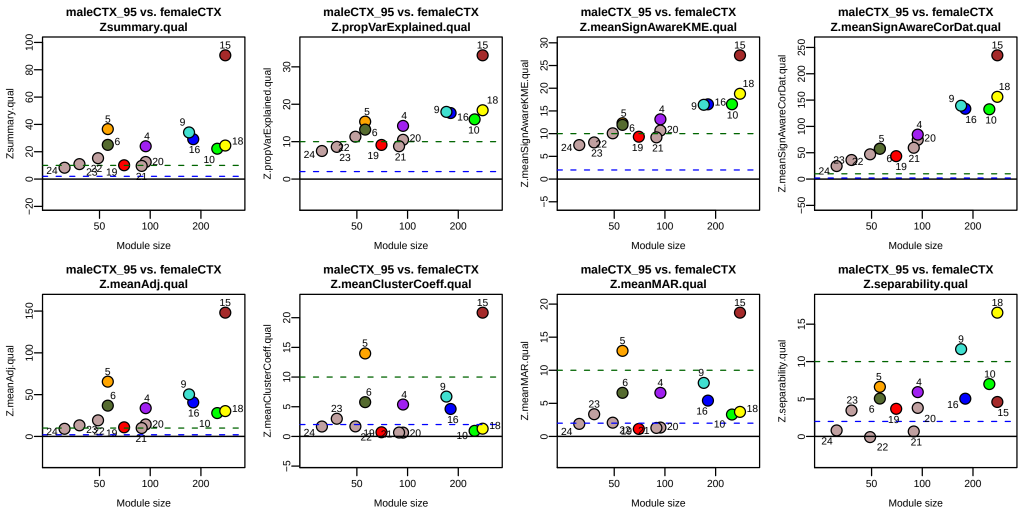

Figure 10. Module quality in the male CTX95 reference data set with the female CTX set as the test network.

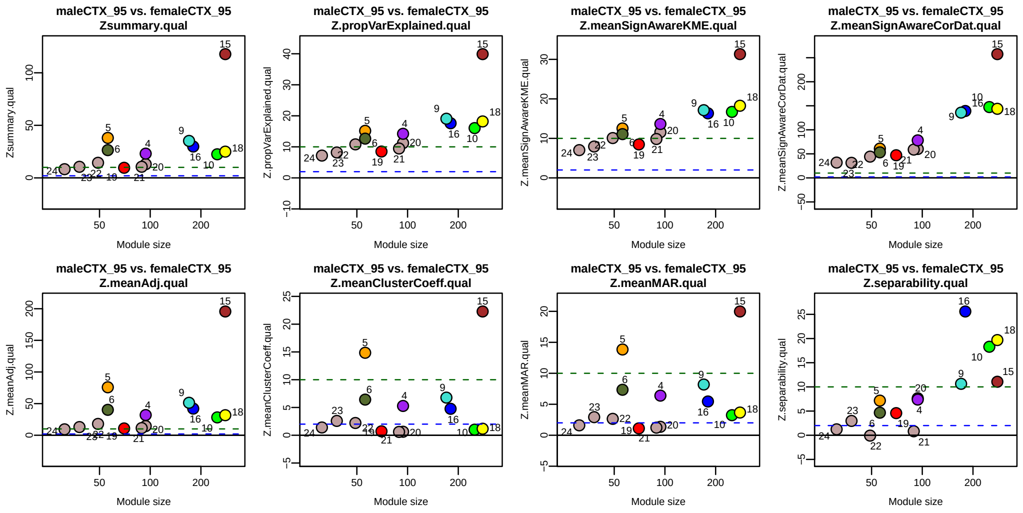

Figure 11. Module quality in the male CTX95 reference data set with the female CTX95 set as the test network.

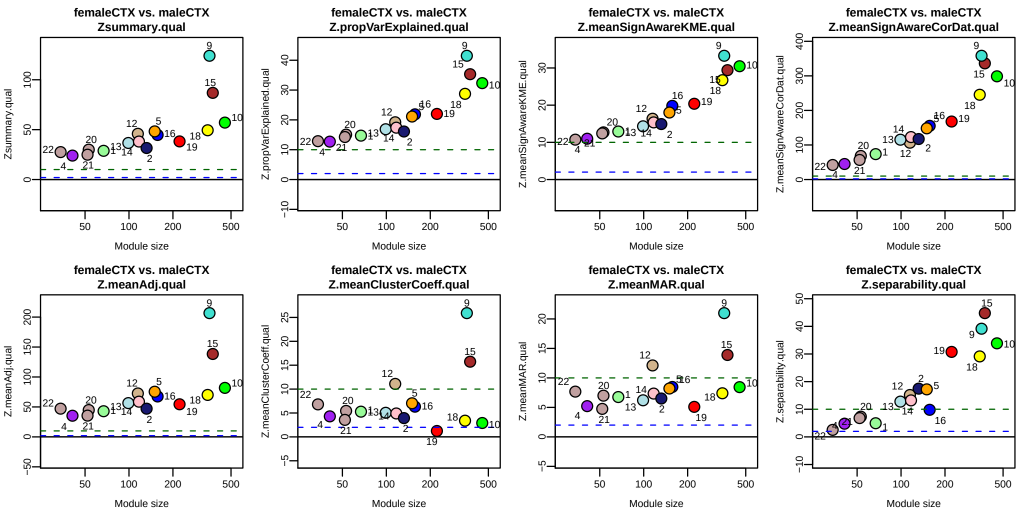

Figure 12. Module quality in the female CTX reference data set with the male CTX set as the test network.

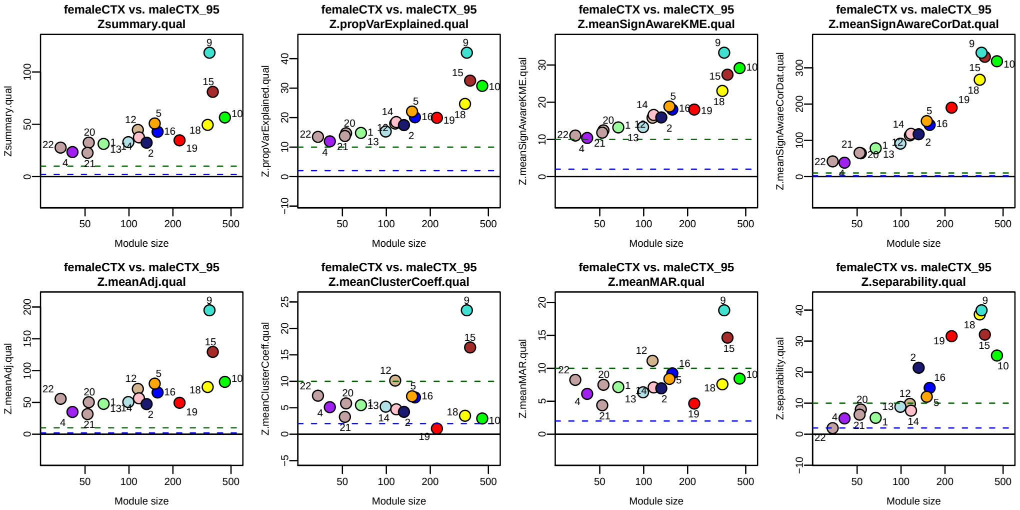

Figure 13. Module quality in the female CTX reference data set with the male CTX95 set as the test network.

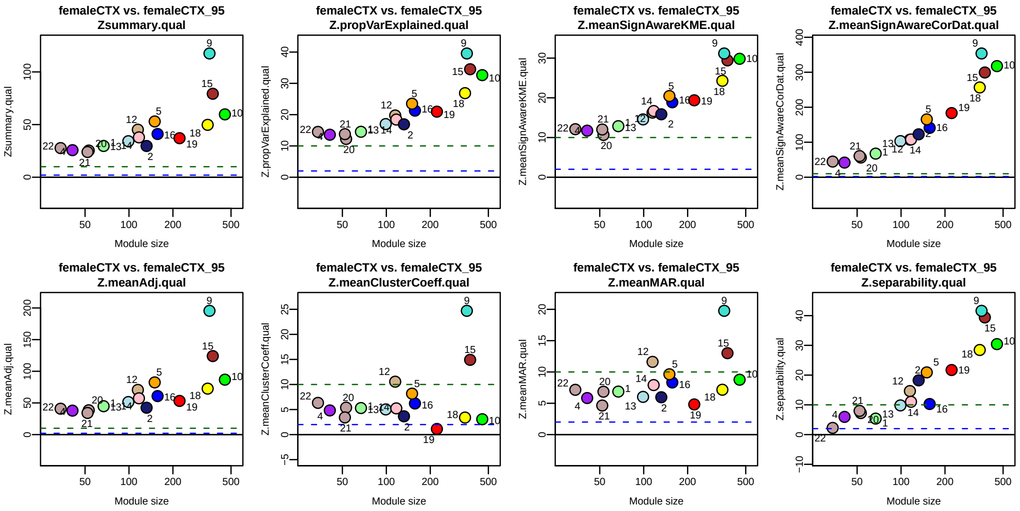

Figure 14. Module quality in the female CTX reference data set with the female CTX95 set as the test network.

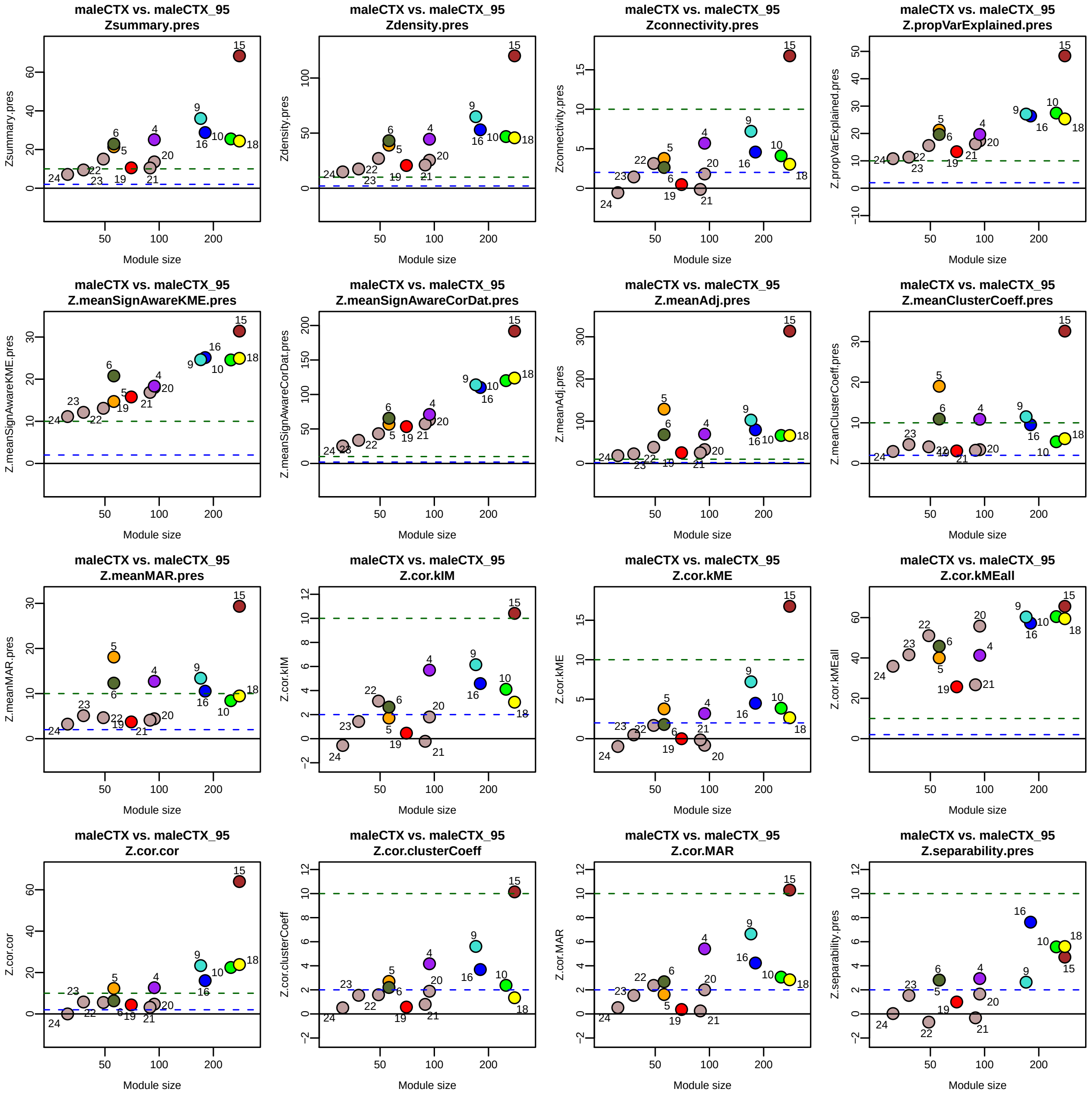

Figure 15. Module preservation between the male CTX reference data set and the male CTX95 test set.

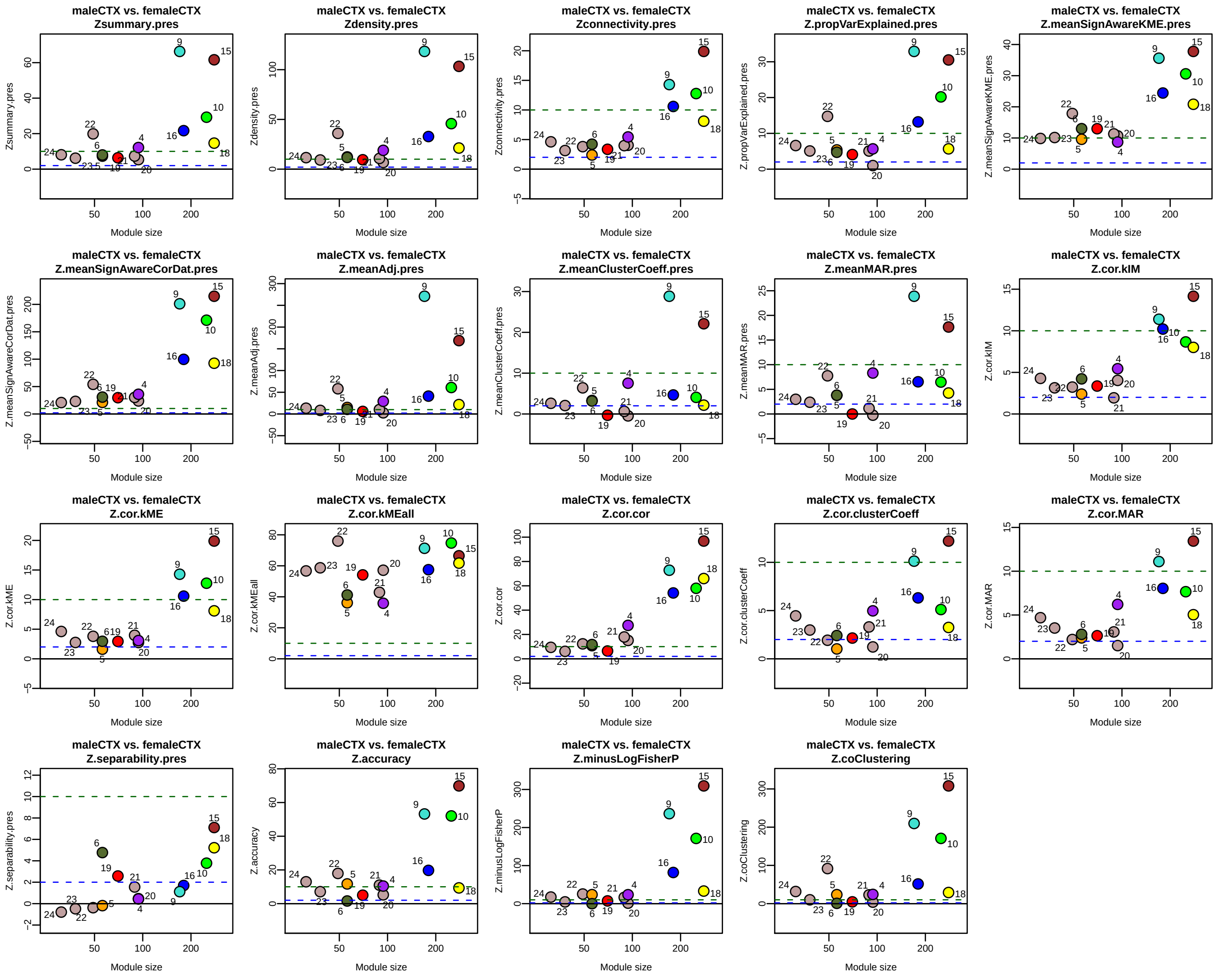

Figure 16. Module preservation between the male CTX reference data set and the female CTX test set.

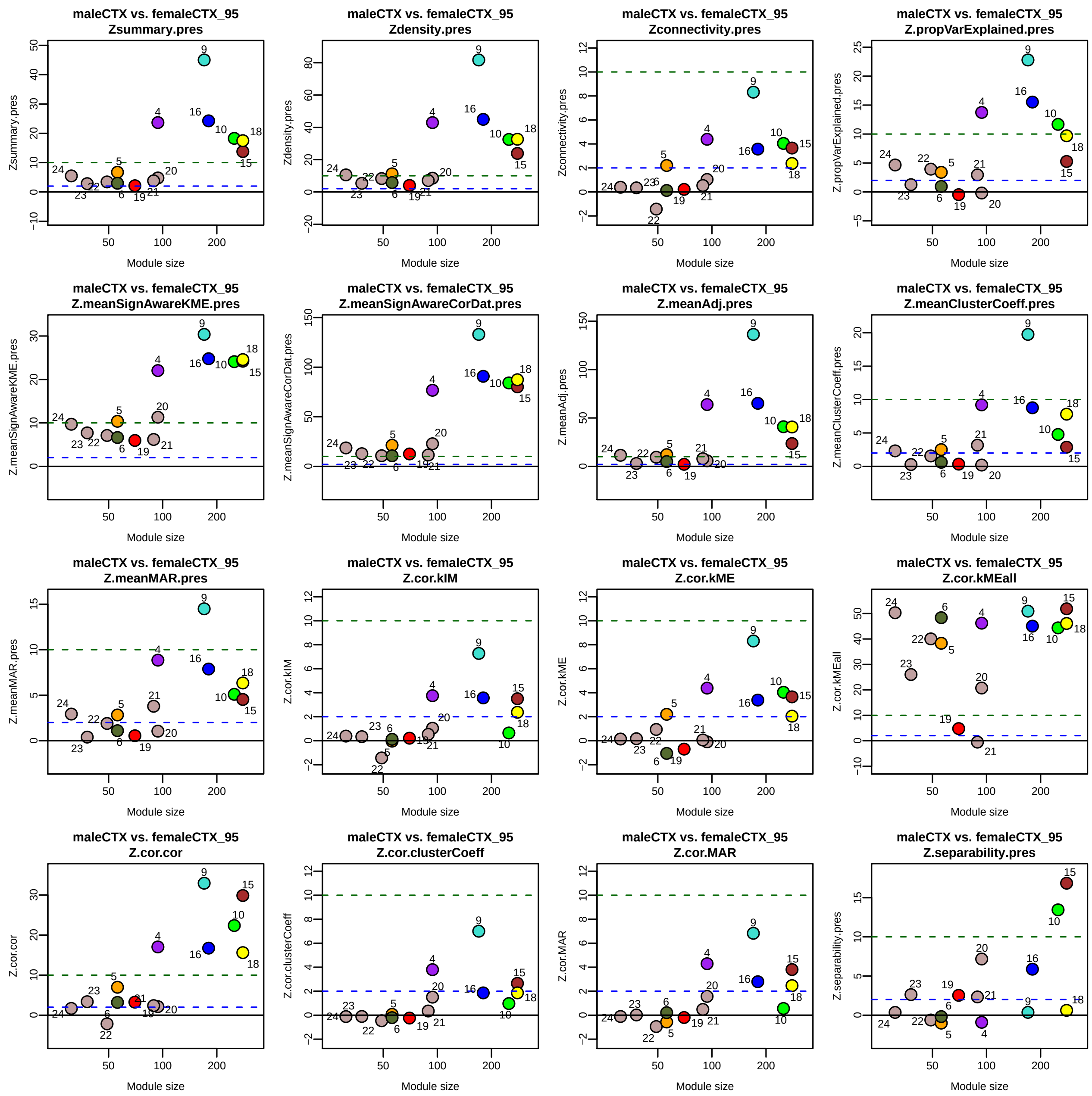

Figure 17. Module preservation between the male CTX reference data set and the female CTX95 test set.

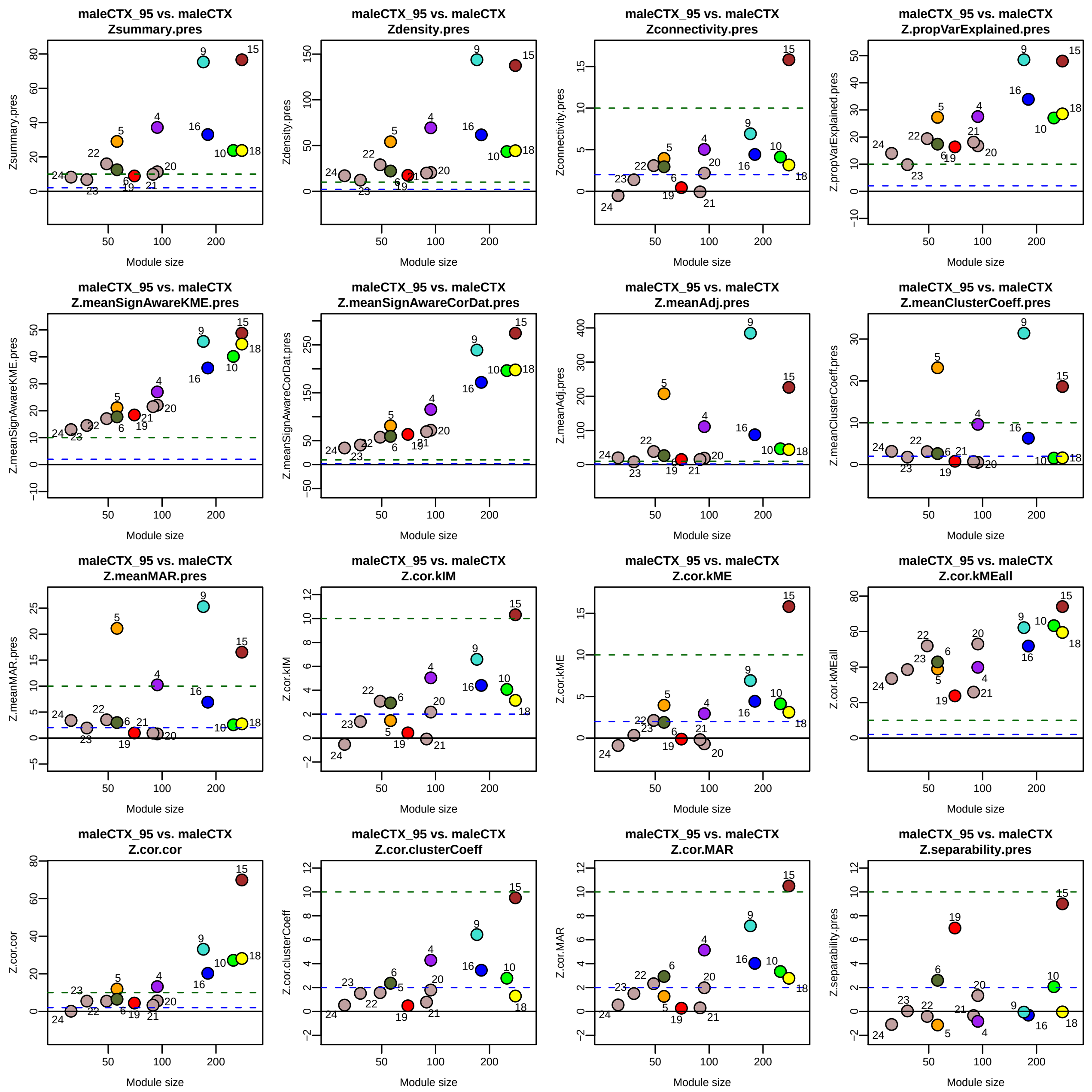

Figure 18. Module preservation between the male CTX95 reference data set and the male CTX test set.

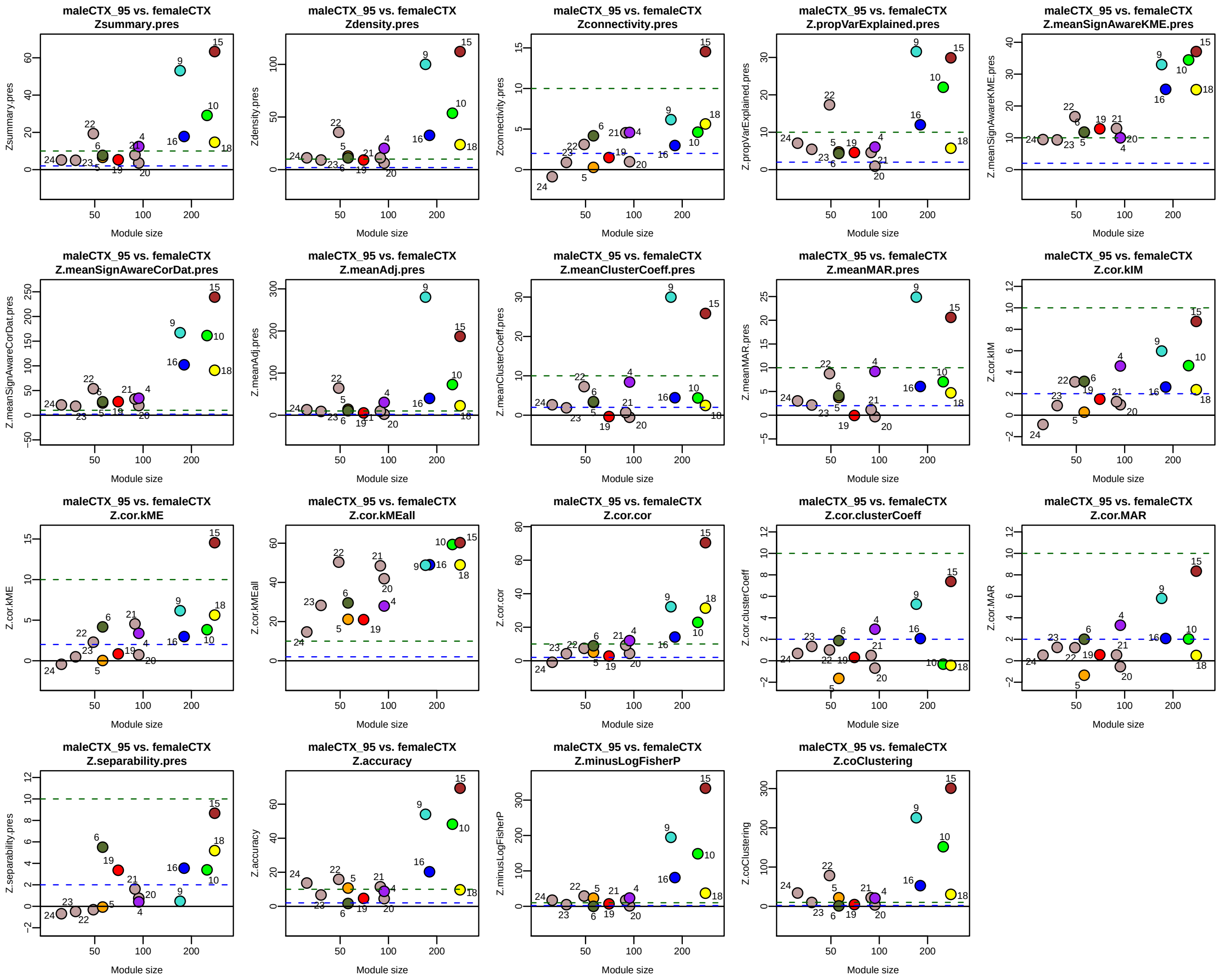

Figure 19. Module preservation between the male CTX95 reference data set and the female CTX test set.

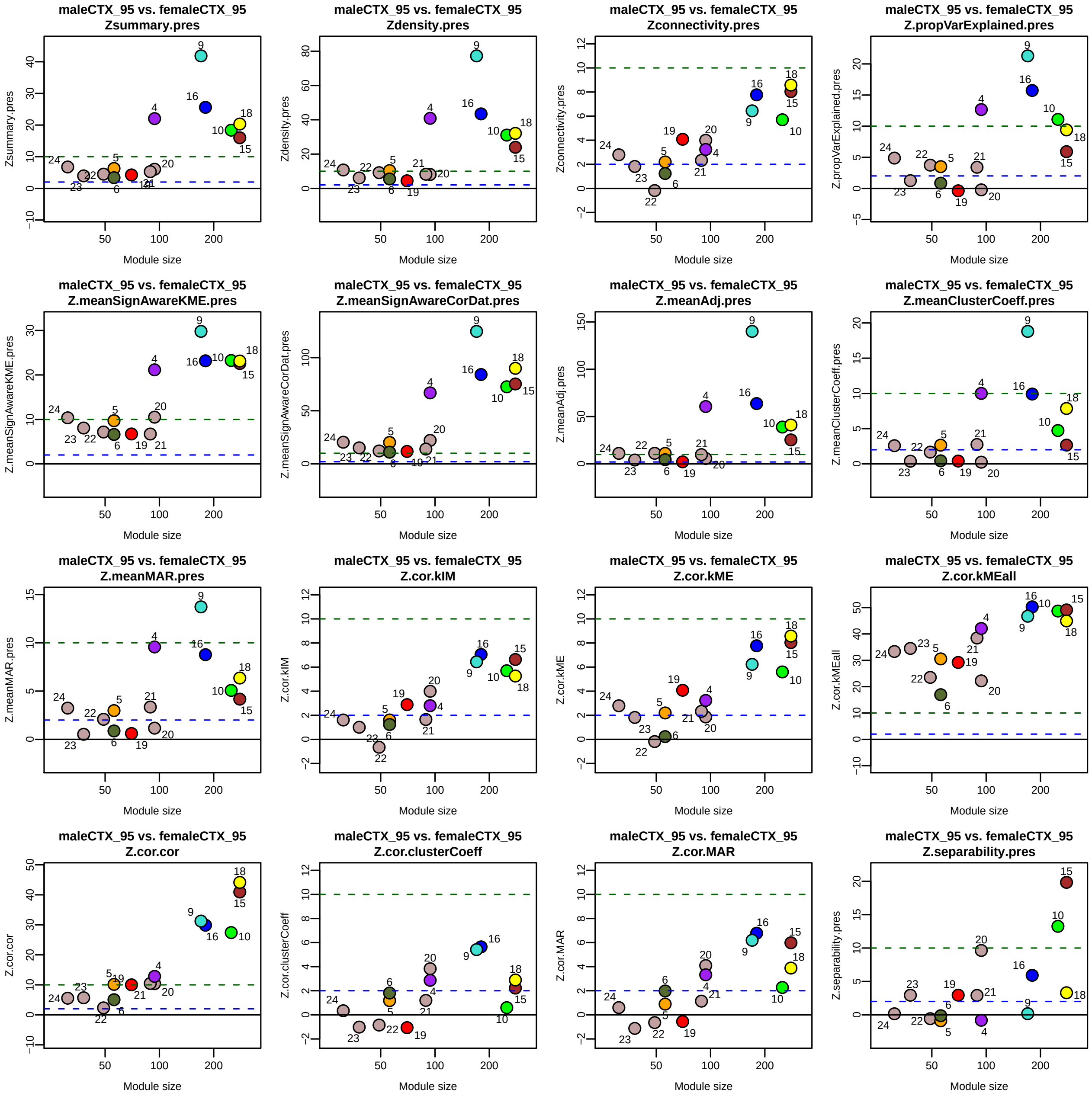

Figure 20. Module preservation between the male CTX95 reference data set and the female CTX95 test set.

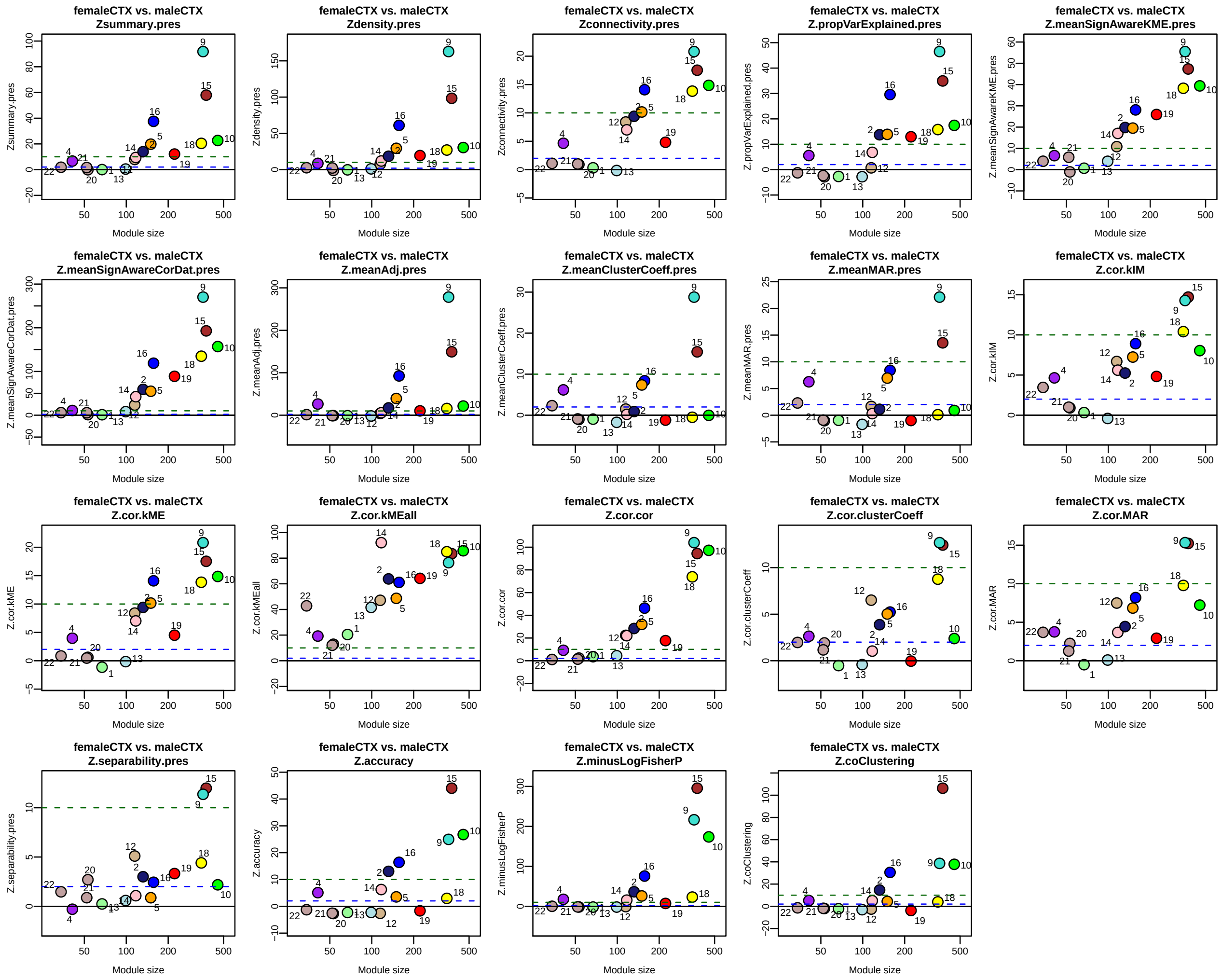

Figure 21. Module preservation between the female CTX reference data set and the male CTX test set.

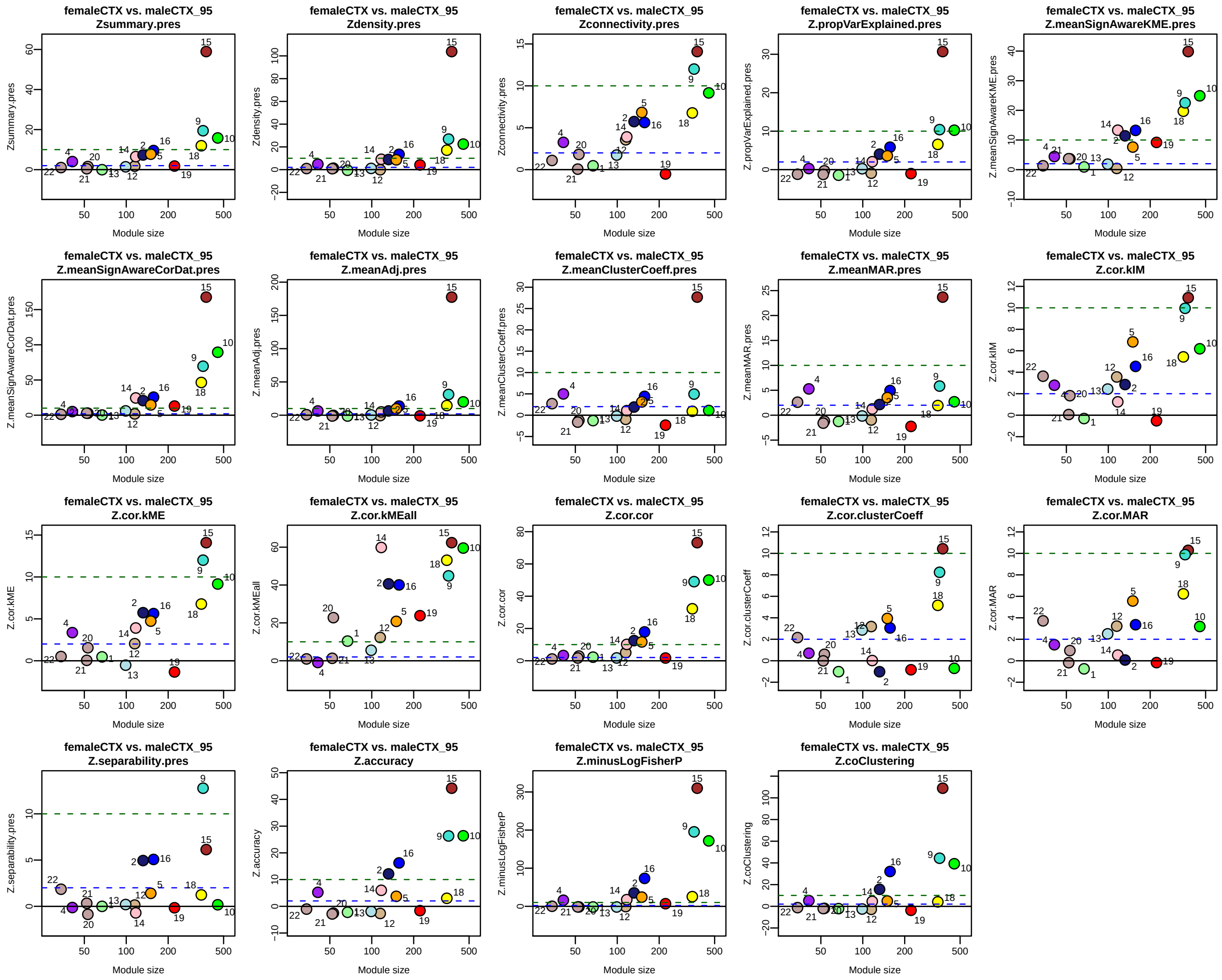

Figure 22. Module preservation between the female CTX reference data set and the male CTX95 test set.

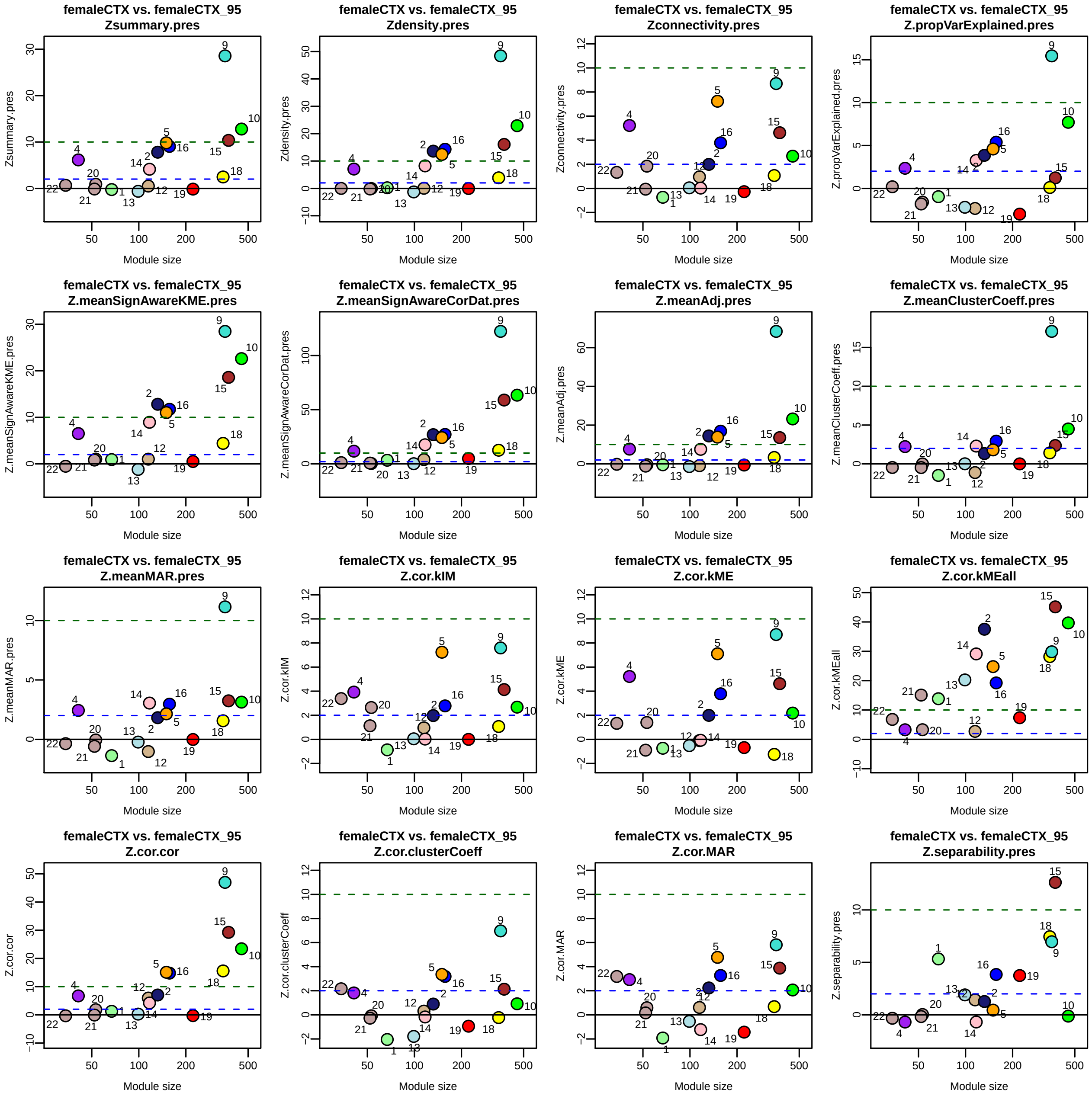

Figure 23. Module preservation between the female CTX reference data set and the female CTX95 test set.
