# Supplementary material for: Is My Network Module Preserved and Reproducible?
Source: PLoS Comput Biol. 2011 Jan 20;7(1):e1001057. doi: 10.1371/journal.pcbi.1001057 (PMC3024255; doi:10.1371/journal.pcbi.1001057)
Supplement: Text S4 — Detailed description of female mouse liver modules in male mice. Detailed results of Application 5: Preservation of female mouse liver modules in male mice. (3.82 MB PDF) [file pcbi.1001057.s009.pdf]

Supplementary Text S4
Detailed results of Application 5: Preservation of female mouse liver modules in male mice

Peter Langfelder, Rui Luo, Michael C. Oldham, and Steve Horvath*
*Corresponding author: shorvath@mednet.ucla.edu

Several studies have explored how co-expression module networks change between mouse tissues [1] and/or genders [2]. Here we re-analyze mouse liver samples of an F2 mouse intercross [3, 4] to study whether "female" modules (i.e., modules found in a network based on female mice) are preserved in the corresponding male network. The data sets consist of 135 female and 124 male samples. The modules were identified in the female data as follows [3]: the original over 20000 probes were reduced to 8000 most variant, and of those the 3600 most connected were selected for network analysis. Further, probes mapping to the same gene were merged, resulting in 3421 measured gene expressions. An unsigned network was constructed using the soft thresholding power $\beta = 6$, genes were clustered using average linkage hierarchical clustering with dissimilarity equal to one minus the topological overlap matrix [5], and the Dynamic Tree Cut [6] was used to identify modules in the clustering tree. The authors of [3] manually merged several modules whose eigengenes were highly correlated. This analysis identified 12 modules (labeled by colors black, blue, brown cyan, green, greenyellow, lightcyan, lightyellow, midnightblue, purple, red, and salmon) with sizes ranging from 34 genes to 772 genes. The biological significance of the modules was discussed in [3]; one of the modules (blue) was found to relate significantly to body weight.

In [3], the male mice were omitted from the analysis since the male network appeared significantly different from the female network. Here we use our module preservation statistics to make this statement more quantitative. Since module identification has not been performed in the male data, we do not use the cross-tabulation methods here and rely instead fully on the network preservation statistics.

We re-created the gene clustering dendrogram in the female data using the same network construction techniques as were used in [3] (Figure 1A). Additionally, we constructed an analogous clustering dendrogram in the male data, but did not identify modules. Instead, in Figure 1B we show the male dendrogram with the corresponding female module colors. This figure provides a visual clue to module preservation. Solid blocks of a single color (such as, for example, brown) indicate that the female module is strongly preserved in the male network. This figure indicates that most modules are likely to be strongly preserved since they can be observed as blocks of solid color under the male dendrogram. The only possible exceptions are the salmon and lightyellow modules that are difficult to locate under the male dendrogram.

The composite statistics $Z_{summary}$ and *medianRank* tell a similar though more precise story. In Figures 1C and D we show $Z_{summary}$ and *medianRank* as a function of module size. The composite $Z_{summary}$ indicates that all modules except the lightyellow and salmon are strongly preserved. For the lightyellow module we find very weak evidence of preservation ($Z_{summary} = 2.0$), while for the salmon module we find moderate evidence for preservation ($Z_{summary} = 7.6$). The salmon and lightyellow modules also exhibit the worst median rank (13) of observed statistics. Detailed results including values of all preservation statistics are presented below and in Supplementary Table S6.

What is the meaning of the weak preservation scores of the lightyellow and salmon modules? Since the network construction is sensitive to outliers, one may suspect that the correlations underlying the lightyellow and salmon modules are caused by outlier samples. Such samples may be visually identified in module expression heatmaps and the corresponding eigengene plots. In Figure 1E we show the heatmap and eigengene plot for the salmon module. It clearly shows an outlier sample in which genes of the salmon module have abnormally high

absolute expression (recall that in this analysis we used an unsigned network). If this sample were removed, the gene-gene correlations within the module would be much lower. For comparison, in Figure 1F we show the analogous heatmap and eigengene plot for the greenyellow module. This module is of similar size (121 genes) as the salmon module (98 genes), but is strongly preserved ($Z_{summary} = 31$, $medianRank = 2.0$). It is significantly enriched for cell cycle genes with a Bonferroni-corrected p-value of 1.5×10^{-42} and thus has a clear functional interpretation. Its heatmap shows a characteristic pattern of vertical bands indicating strongly co-expressed genes. None of the samples appears to be an outlier, and certainly removing any few samples of the module would not drastically alter the co-expression pattern. The heatmap of the lightyellow module (not shown) exhibits a pattern similar to the salmon module, with one outlier sample underlying most of the gene-gene correlations. *In summary, this application demonstrates that module preservation statistics allow us to identify invalid, non-reproducible modules due to array outliers.*

In the fourth section of Supplementary text S11, we present module quality statistics that are implemented in the `modulePreservation` R function. These module quality measures indicate that the lightyellow and salmon modules are high quality modules in the reference data set, that is their module density is high (see below). This is because Pearson correlation is sensitive to outliers. Were one to use an outlier-resistant correlation such as the biweight midcorrelation [7,8], the lightyellow and salmon modules would have been flagged as low-quality modules just by analyzing the module quality in the reference data, without the need of a test data set. Our R software implementation allows the user to choose the biweight midcorrelation instead of Pearson correlation.

A comprehensive table of module preservation statistics can be found in the accompanying Supplementary Table S3, a plain text Comma Separated Value format file. In Figure 2 we present detailed plots of the preservation Z statistics as a function of module size. In Figures 3 and 4 we present scatterplots of various measures of connectivity for two highly preserved modules (brown and green) and for the two (lightyellow and salmon) weakly preserved modules. In Figures 5 and 6 we present module heatmaps and corresponding eigengene expression barplots for the highly preserved modules and the weakly preserved modules, respectively. The heatmaps indicate the reason why the lightyellow and salmon modules are weakly preserved: the co-expression of their genes in the female data is mainly caused by a single outlier sample. In this sense the modules can be considered technical artefacts.

Results of alternate methods

Here we present the results of the function `clusterRepro` [9]. The method would take an excessively long time to calculate meaningful p-values, and hence we only report the observed IGP values, Figure 7. The lightyellow and salmon modules have the lowest IGP in the male data (panel A2).

Since we have a clustering for the male data, in this application we can also evaluate cross-tabulation measures. Figure 8 shows accuracy, Fisher exact test p-value, and co-clustering for the female modules when the male clustering is used as test clustering. The lightyellow and salmon modules are conspicuous by their low values of all three quantities. Thus, in this case the cross-tabulation statistics identify the same modules as the network preservation statistics.

References

1. Keller MP, Choi Y, Wang P, Belt Davis D, Rabaglia ME, et al. (2008) A gene expression network model of type 2 diabetes links cell cycle regulation in islets with diabetes susceptibility. Genome Res 18: 706-716.

2. van Nas A, GuhaThakurta D, Wang SS, Yehya N, Horvath S, et al. (2009) Elucidating the Role of Gonadal Hormones in Sexually Dimorphic Gene Coexpression Networks. Endocrinology 150: 1235-1249.

3. Ghazalpour A, Doss S, Zhang B, Plaisier C, Wang S, et al. (2006) Integrating genetics and network analysis to characterize genes related to mouse weight. PloS Genetics 2: 8.

4. Fuller T, Ghazalpour A, Aten J, Drake T, Lusis A, et al. (2007) Weighted gene coexpression network analysis strategies applied to mouse weight. Mammalian Genome 18: 463–472.

5. Zhang B, Horvath S (2005) General framework for weighted gene coexpression analysis. Statistical Applications in Genetics and Molecular Biology 4.

6. Langfelder P, Zhang B, Horvath S (2007) Defining clusters from a hierarchical cluster tree: the Dynamic Tree Cut library for R. Bioinformatics 24: 719–20.

7. Wilcox RR (1997) Introduction to Robust Estimation and Hypothesis Testing. Academic Press.

8. Hardin J, Mitani A, Hicks L, VanKoten B (2007) A robust measure of correlation between two genes on a microarray. BMC Bioinformatics 8: 220.

9. Kapp AV, Tibshirani R (2007) Are clusters found in one dataset present in another dataset? Biostat 8: 9-31.

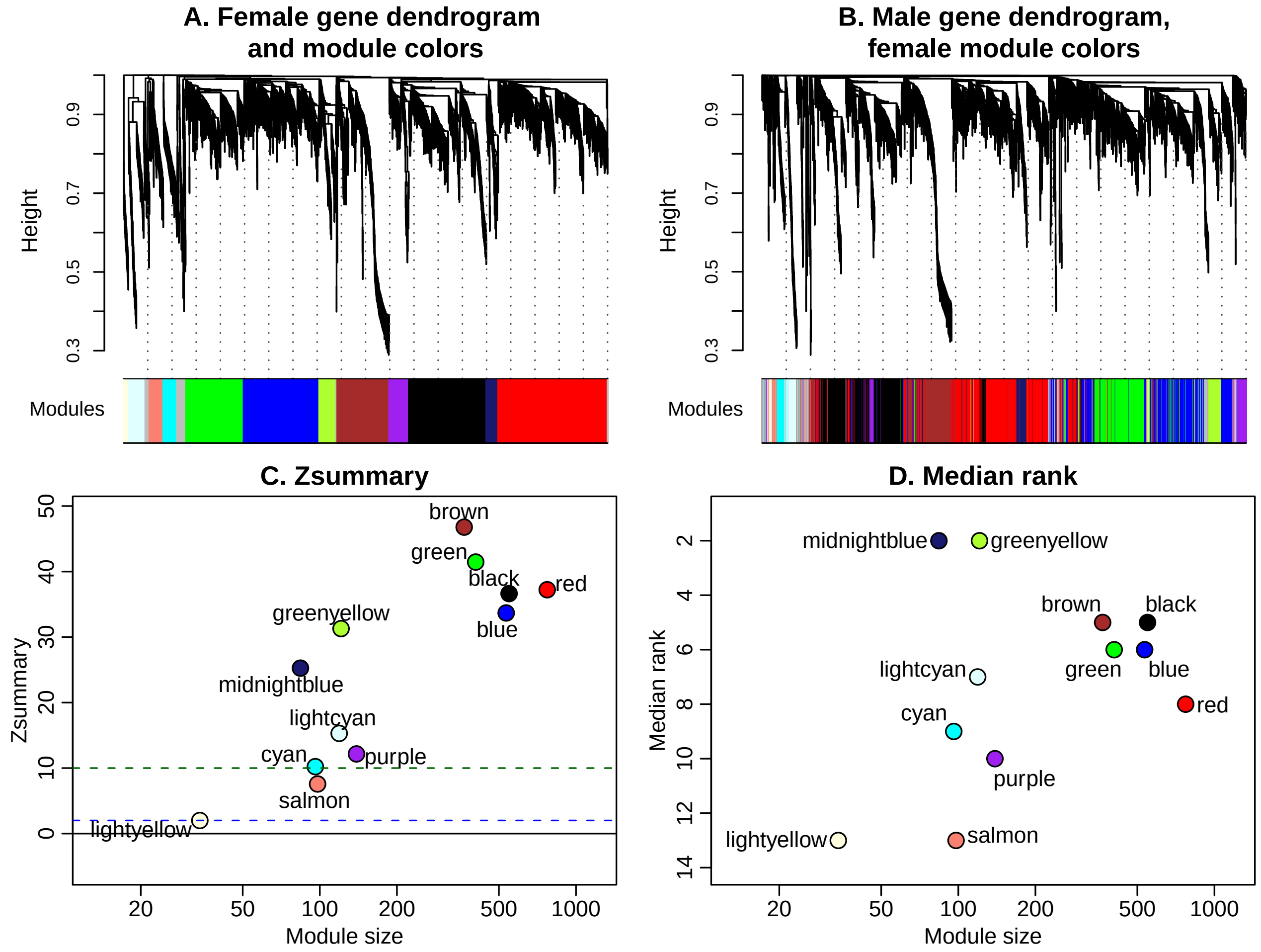

Figure 1. Preservation of female mouse liver modules in male samples of the same cross. A. Hierarchical clustering tree (dendrogram) of genes based on the female mouse liver co-expression network. Each "leaf" (short vertical line) corresponds to one gene. The color row below the dendrogram indicates module membership in the modules defined by cutting branches of this dendrogram using the Dynamic Tree Cut algorithm [6]. B. Hierarchical clustering tree of genes based on the male mouse liver co-expression network. The color row below the dendrogram indicates module membership in the female modules (defined by cutting branches of dendrogram in panel A.) C. Composite preservation statistic $Z_{summary}$ (y-axis) of preservation of female modules in the male network as a function of module size (x-axis). Each point corresponds to a module labeled by color and the text label. The blue and green dashed lines indicate the thresholds $Z_{summary} = 2$ and $Z_{summary} = 10$, respectively. D. The median rank of observed preservation statistics (y-axis) of preservation of female modules in the male network as a function of module size (x-axis). Each point corresponds to a module labeled by color and the text label.

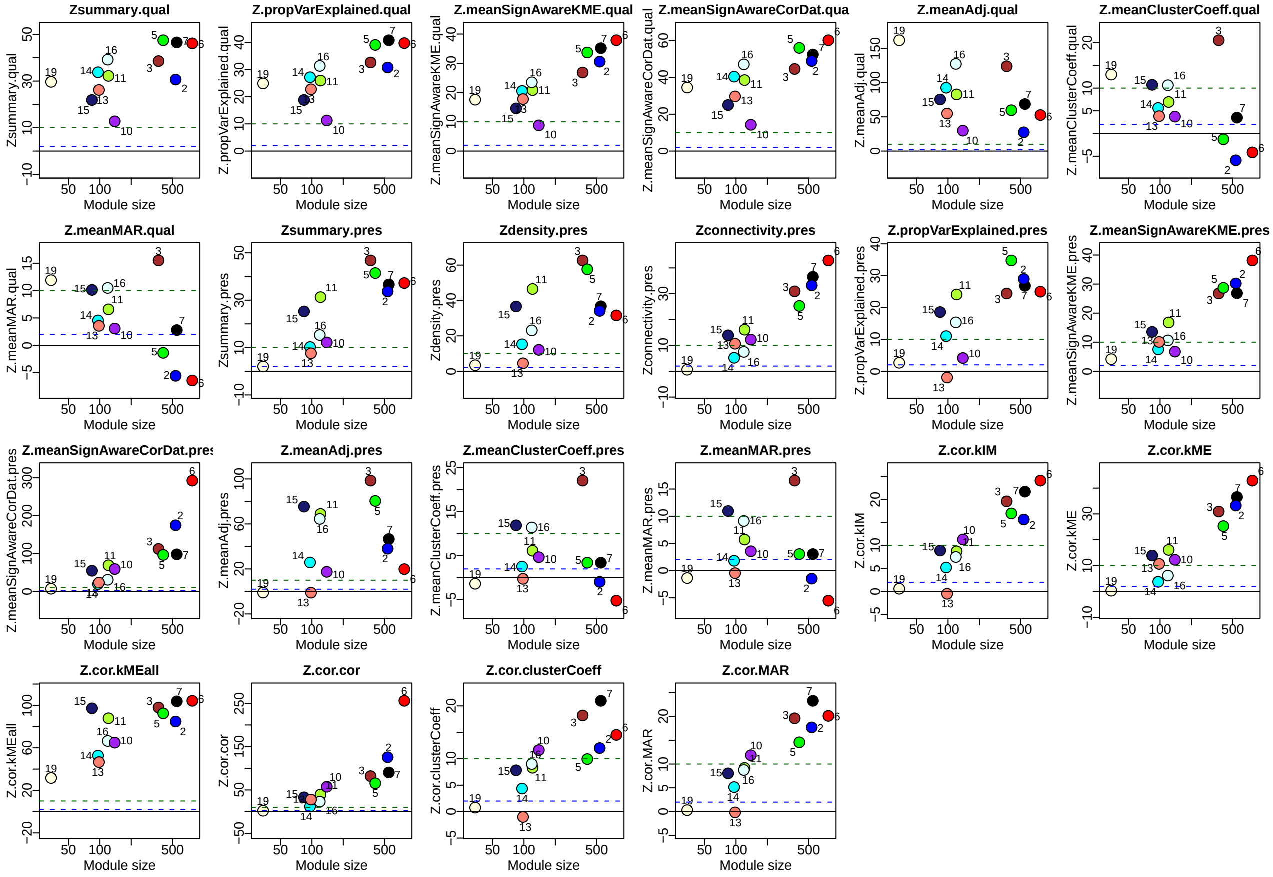

Figure 2. Z scores of quality and preservation of female mouse liver modules in male samples. Each plot presents one Z statistic (identified in the title) as a function of the module size. Each point represents a module, labeled by a color and a numeric label (2, blue; 3, brown; 5, green; 6, red; 7, black; 10, purple; 11, greenyellow; 13, salmon; 14, cyan; 15, midnightblue; 16, lighcyan; 19, lightyellow). The dashed blue and green lines indicate thresholds of $Z = 2$ and $Z = 10$, respectively.

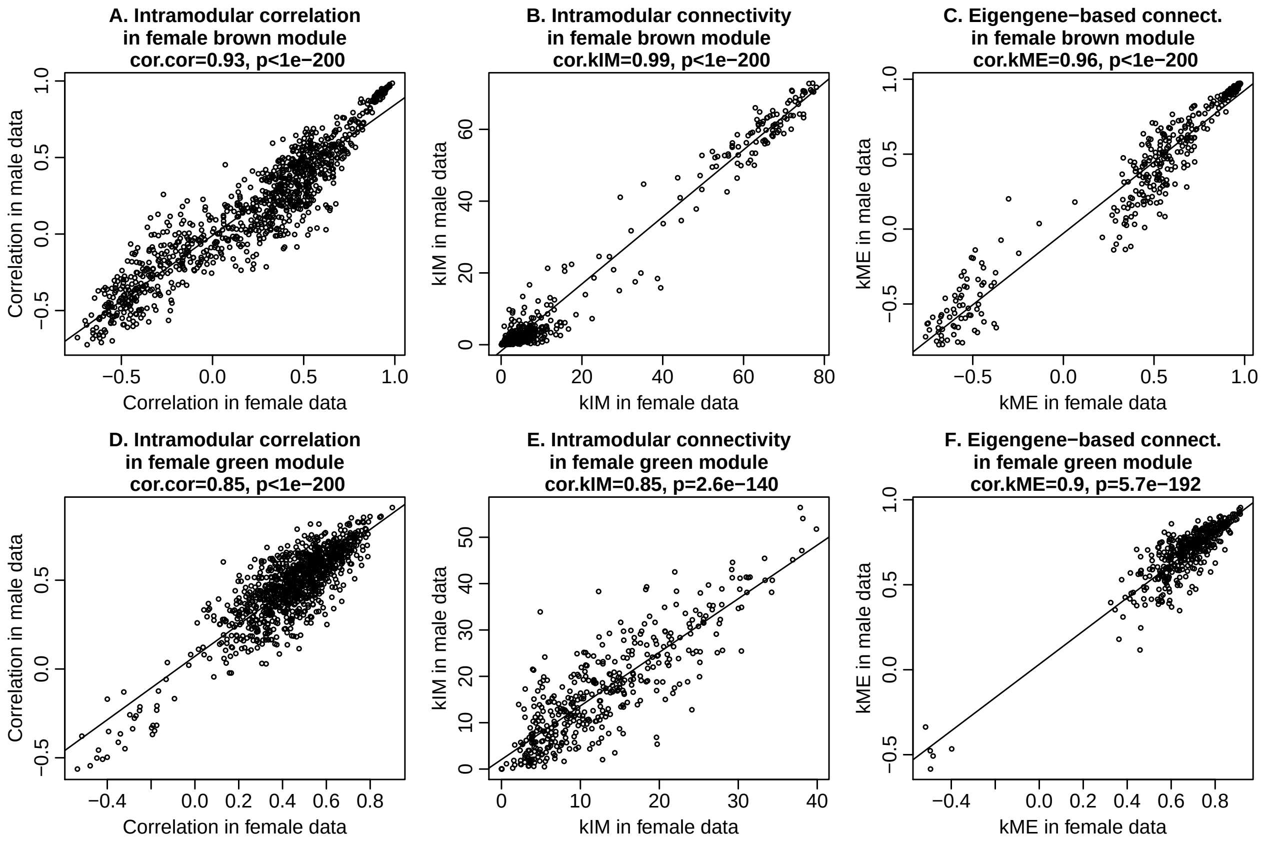

Figure 3. Scatterplots of various connectivity measures of genes in two highly preserved modules (blue and green). Each plot shows a connectivity measure of genes in one of the modules in the male data (y-axis) vs. the same connectivity measure in the female data (x-axis). The connectivity measure and module label are indicated in the title of each plot. The titles also contain the correlations and corresponding p-values of the male and female connectivities in each plot. The correlations and p-values are extremely high, indicating very strong preservation of connectivity patterns in the brown and green modules between the female and male samples.

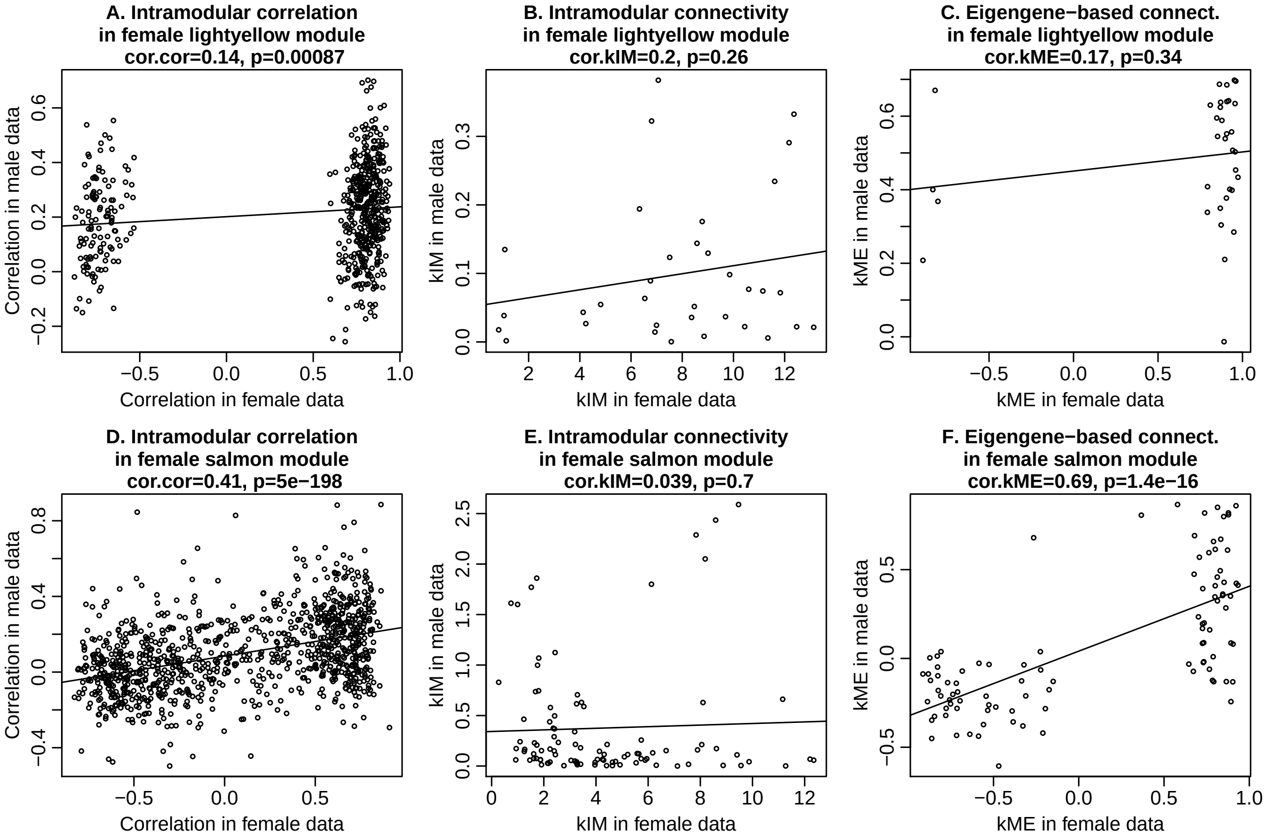

Figure 4. Scatterplots of various connectivity measures of genes in the two modules with lowest $Z_{summary}$ preservation statistics (lightyellow and salmon). Each plot shows a connectivity measure of genes in one of the modules in the male data (y-axis) vs.the same connectivity measure in the female data (x-axis). The connectivity measure and module label are indicated in the title of each plot. The titles also contain the correlations and corresponding p-values of the male and female connectivities in each plot. The correlations and p-values are much lower than those in strongly preserved modules (Figure 3), indicating weak preservation of connectivity patterns in the lightyellow and salmon modules between the female and male samples.

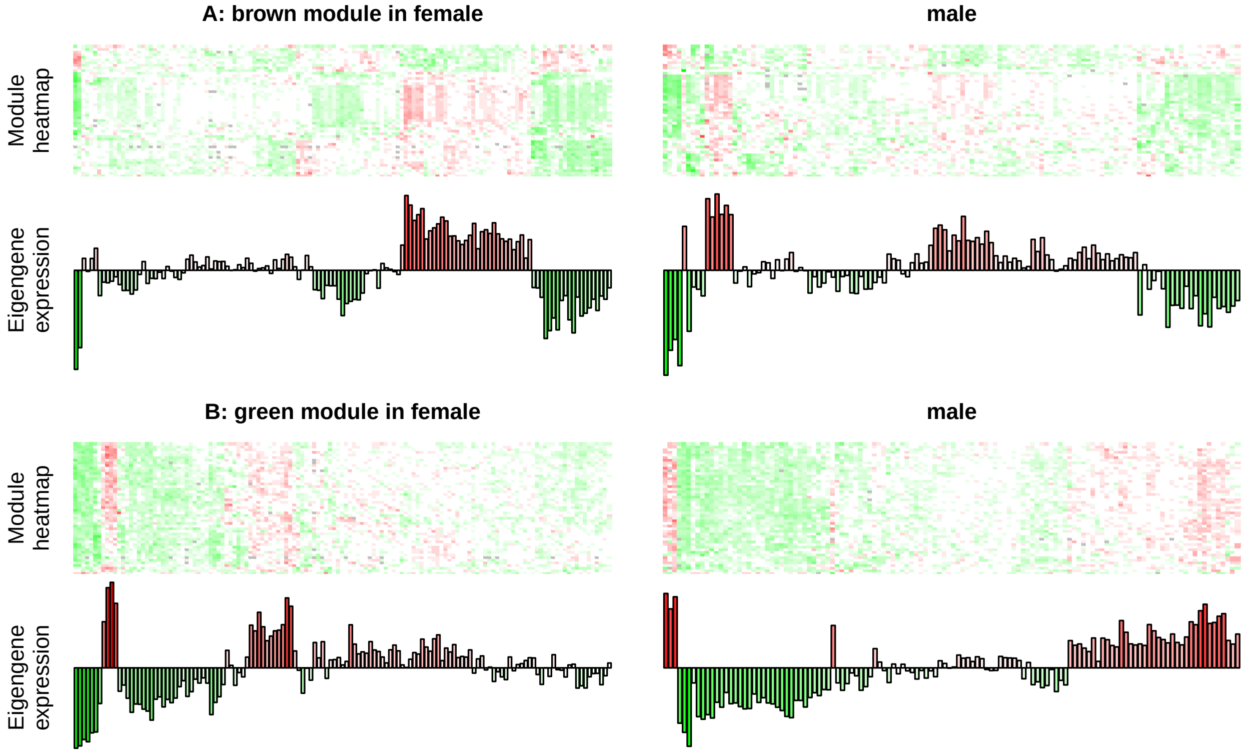

Figure 5. Module heatmaps and eigengene expression barplots of highly preserved modules in female and male samples. Each plot contains a module heatmap and a barplot of eigengene expression of one module in one data set (indicated in the title). In the heatmap, rows correspond to genes and column to samples; green means under-expression, red over-expression and white average expression; missing data are indicated by grey color. The eigengene summarizes the expression of all genes in the module. The heatmaps exhibit characteristic vertical bands indicating strong and robust co-expression of genes within each module in both tissues.

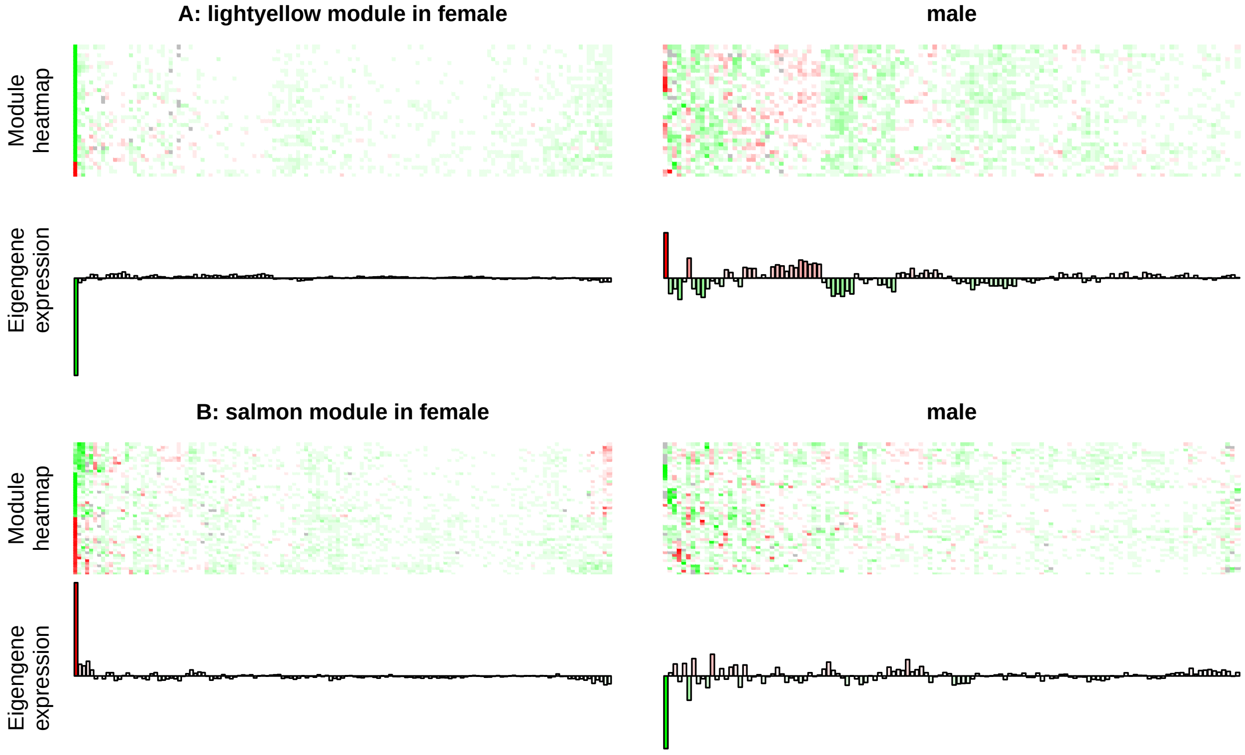

Figure 6. Module heatmaps and eigengene expression barplots of the weakly preserved modules in female and male samples. Each plot contains a module heatmap and a barplot of eigengene expression of one module in one data set (indicated in the title). In the heatmap, rows correspond to genes and column to samples; green means under-expression, red over-expression and white average expression; missing data are indicated by grey color. The eigengene summarizes the expression of all genes in the module. Heatmaps in the female samples indicate that in each of the modules the co-expression is mainly driven by one outlier sample. Hence, these two modules appear to be artefacts of the presence of an outlier sample.

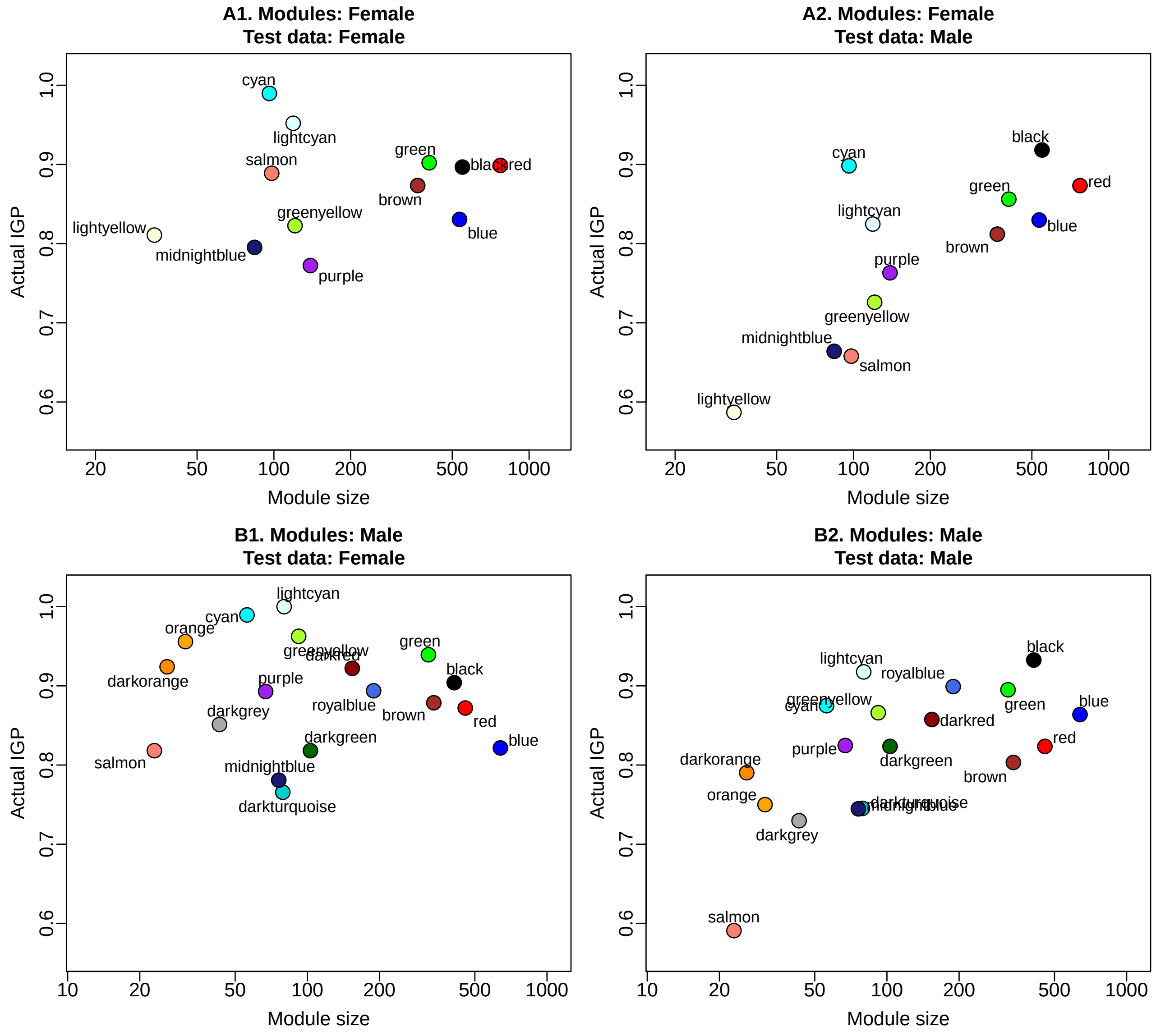

Figure 7. Observed in-group proportion of female and male liver modules when both data sets are used as test data. Modules are labeled by their labels and colors. The title of each plot indicates which clustering ("Modules:") was applied to which set ("Test data:") to obtain the IGP displayed in the plot. When the female modules are tested in male data (panel A2), the lightyellow and salmon modules exhibit a lower IGP and hence preservation than other similarly sized modules.

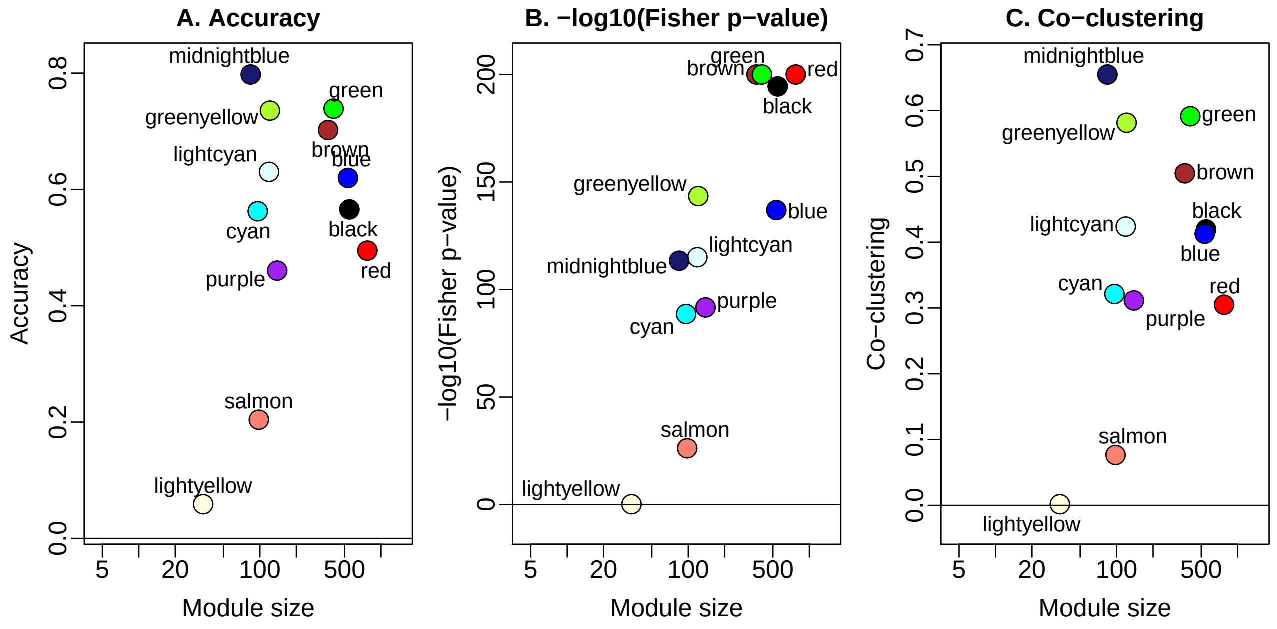

Figure 8. Observed values of cross-tabulation statistics of female mouse liver modules when compared to the corresponding male liver modules. Modules are labeled by their labels and colors. The lightyellow and salmon modules exhibit lower preservation than other modules.
