# Supplementary material for: Is My Network Module Preserved and Reproducible?
Source: PLoS Comput Biol. 2011 Jan 20;7(1):e1001057. doi: 10.1371/journal.pcbi.1001057 (PMC3024255; doi:10.1371/journal.pcbi.1001057)
Supplement: Text S5 — Detailed description of the consensus module application. Here we study preservation of consensus modules constructed previously, namely the consensus modules across human and chimpanzee brain samples, across samples from 4 tissues of female mice, and across samples from male and female mouse livers. (1.41 MB PDF) [file pcbi.1001057.s010.pdf]

Supplementary text S5
Application 6: Preservation of consensus modules

Peter Langfelder, Luo Rui, Michael C. Oldham, and Steve Horvath*
*Corresponding author: shorvath@mednet.ucla.edu

Here we study preservation of consensus modules constructed previously in [1], namely the consensus modules across human and chimpanzee brain samples, across samples from 4 tissues of female mice, and across samples from male and female mouse livers.

Consensus modules [1] across given data sets are constructed in such a way that genes within consensus modules are highly co-expressed in all given individual data sets. For example, consensus modules across human and chimpanzee brains should exhibit a high preservation between the human and chimpanzee data sets. Here we show that this is indeed true on the examples analyzed in [1]. That work reported three examples of consensus module analysis: 1) across human and chimpanzee brain expression data, 2) across expression data from mouse female brain, muscle, liver, and adipose tissues (i.e., consensus of four separate data sets), and 3) across female and male mouse liver expression data. The human and chimpanzee expression data have been first reported in [2], and a gene co-expression network analysis was described in [3]. The mouse expression data have been obtained from a BxH F2 intercross and have been previously described in [4].

A summary of the results is shown in Figure 1. The Figures shows that most consensus modules show strong overall preservation as measured by the $Z_{summary}$ statistic, with only a few exceptions showing moderate preservation. This expected findings corroborates that our module preservation statistics are indeed capable of identifying preserved modules. We observe again that, in general, larger modules tend to have larger Z statistic values. Since the Z statistics measure how significantly different the observed statistics are from their expectation under the null hypothesis (of no preservation) it is not surprising that this significance level depends on the module size.

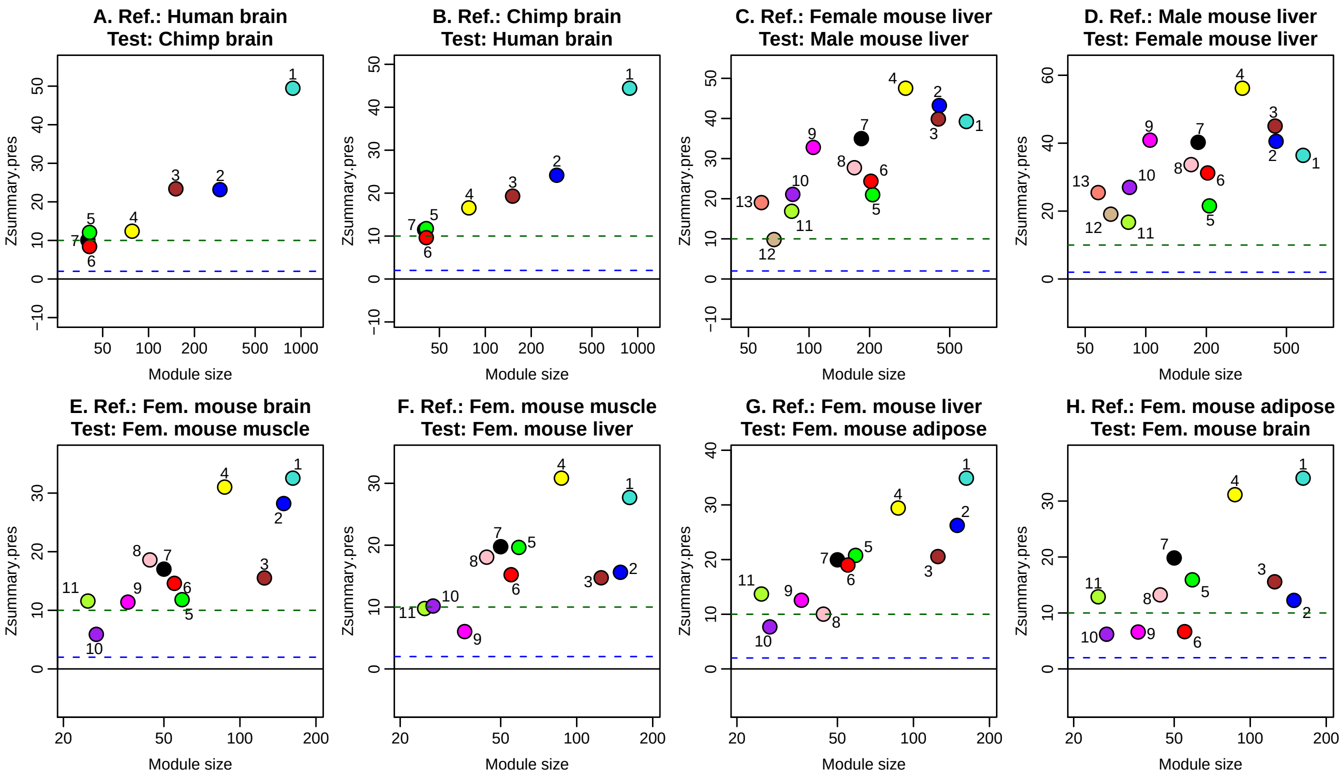

Figure 1. Consensus modules show high preservation statistics. This figure shows the $Z_{summary}$ statistics for human–chimpanzee consensus modules (panels A., B.), female–male mouse liver consensus modules (panels C., D.), and consensus modules across four female tissues (panels E.–H.). Each panel indicates which data set was regarded as reference (Ref.) and which data set was regarded as the test set. Each panel shows the preservation statistic $Z_{summary} = \frac{Z_{medianDensity} + Z_{medianNetStruct}}{2}$ as a function of the module size (x-axis) for the modules found in the corresponding analysis in [1]. Colors of the plot symbols correspond to the module color labels in [1]. Modules can also be identified by the secondary numeric label, 1 denoting turquoise, 2 blue, 3 brown, 4 yellow, 5 green, 6 red, 7 black, 8 pink, 9 magenta, 10 purple, and 11 greenyellow. The dashed blue and green lines indicate the thresholds $Z = 2$ and $Z = 10$, respectively. For all reference and test data sets, all consensus modules exhibit preservation statistics of around or above 10 indicating strong preservation.

In summary, this application shows 1) that module preservation statistics and the recommended threshold values provide sufficient statistical power to implicate preserved modules in several real examples and 2) that the Z statistic values of preserved modules tend to increase with module size.

A complete table of results can be found in the accompanying Supplementary Table S4. This is a flat comma separated value (CSV) text file that can be viewed in most standard spreadsheet software such as MS Excel and OpenOffice Calc. The columns indicate the reference set, test set, module, module size, observed preservation statistics, and their Z scores.

In Figures 2– 17 we present selected Z scores graphically. The plots are organized to form one figure per reference-test pair. In each figure, we plot the module quality and preservation Z scores (y-axis) of density and network structure preservation statistics as a function of module size (x-axis). The reference and test data sets are indicated in the title of each plot. Colors of the plot symbols correspond to the module color labels in [1]. Modules can also be identified by the secondary numeric label, 1 denoting turquoise, 2 blue, 3 brown, 4 yellow, 5 green, 6 red, 7 black, 8 pink, 9 magenta, 10 purple, and 11 greenyellow. The dashed blue and green lines indicate the thresholds $Z = 2$ and $Z = 10$, respectively.

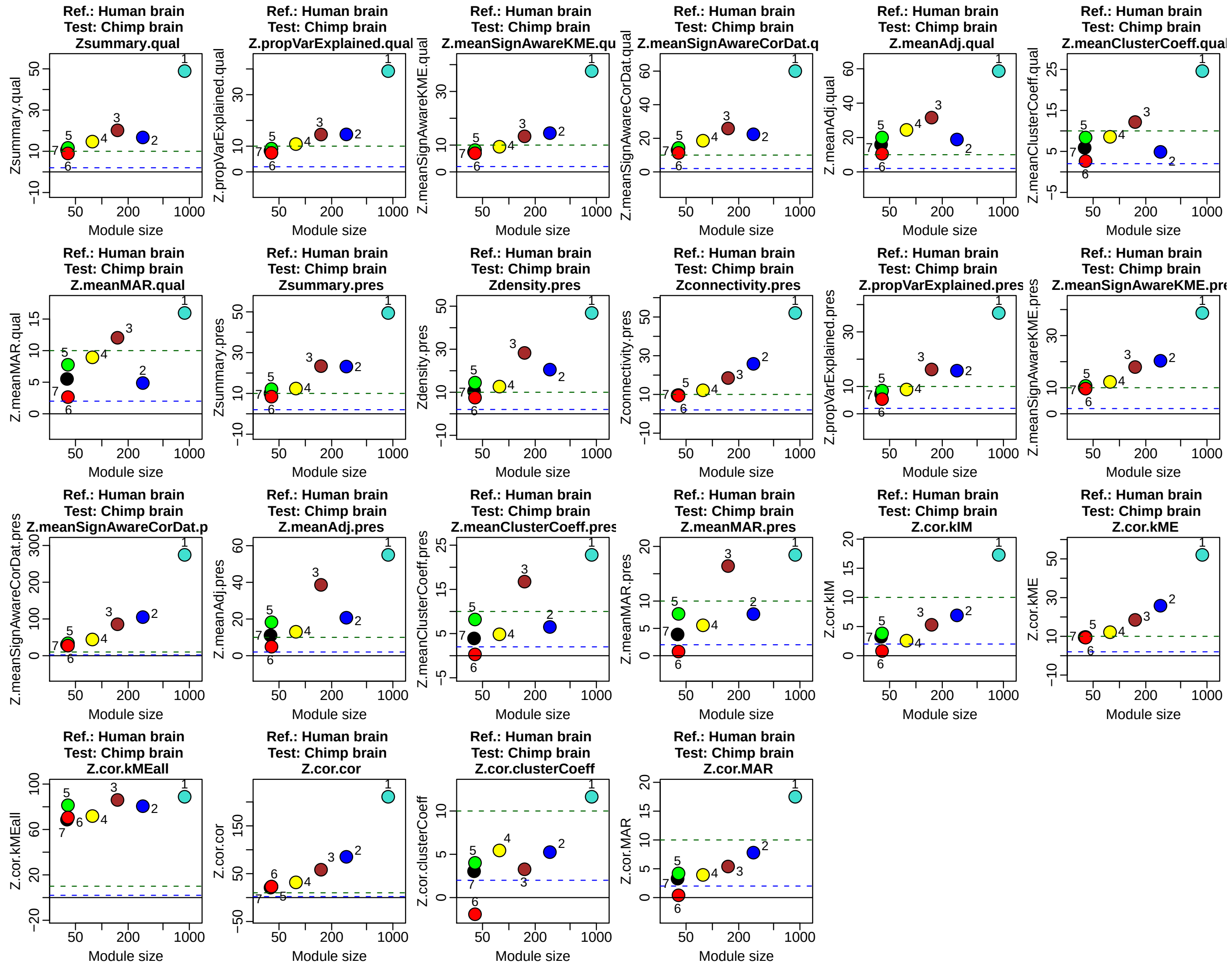

Figure 2. Module preservation of consensus modules between human and chimpanzee brain samples.

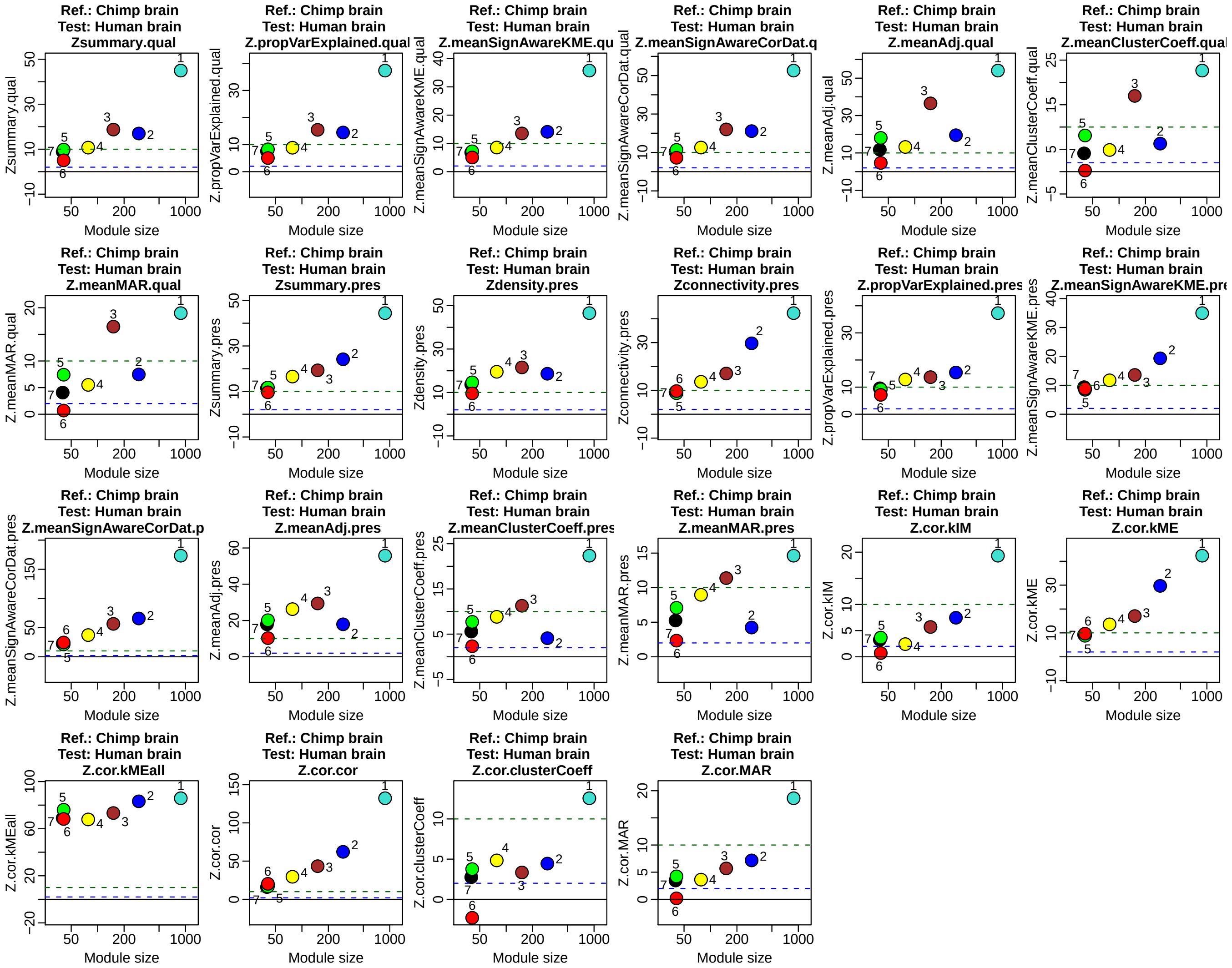

Figure 3. Module preservation of consensus modules between chimpanzee and human brain samples.

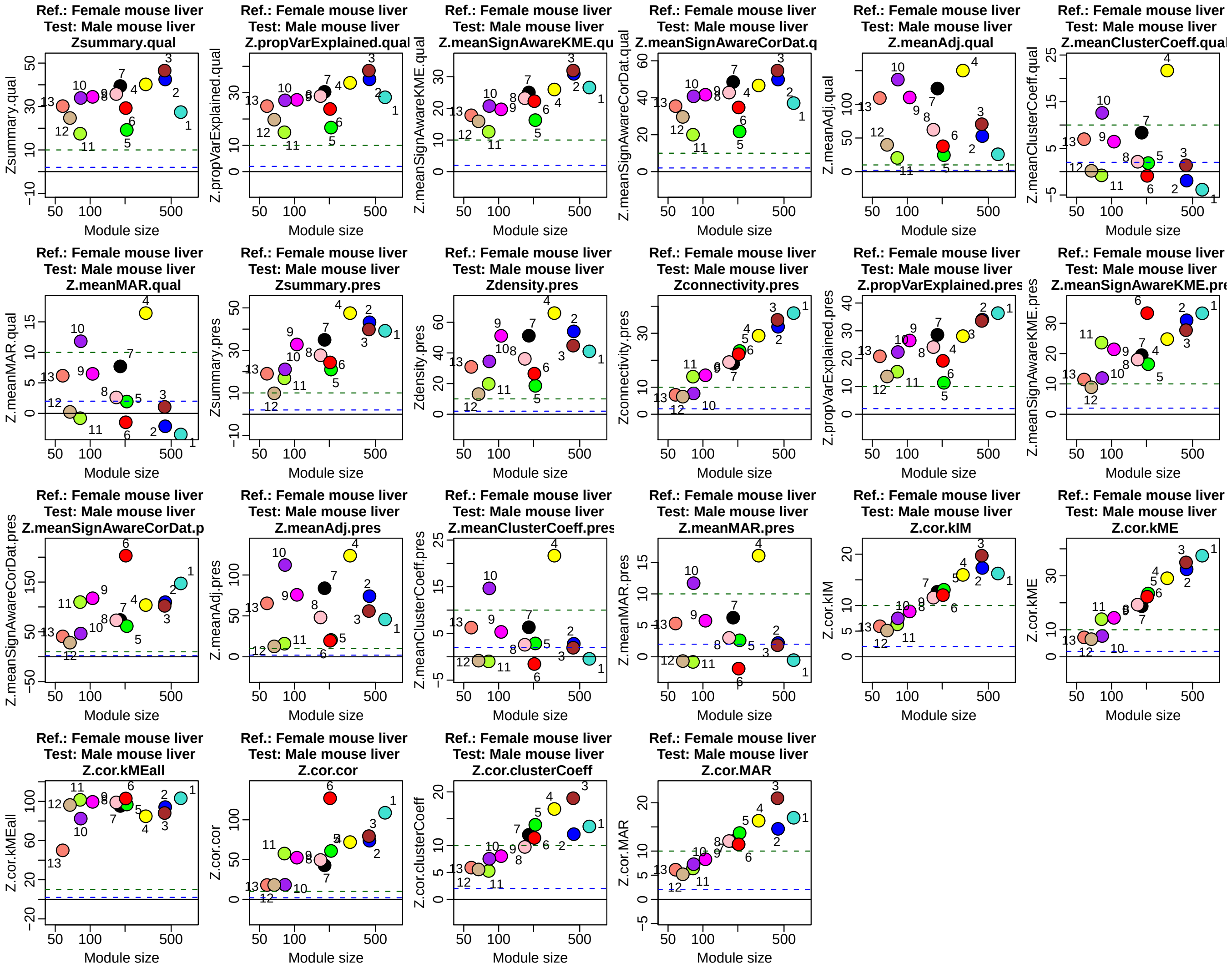

Figure 4. Module preservation of consensus modules between female and male mouse liver samples.

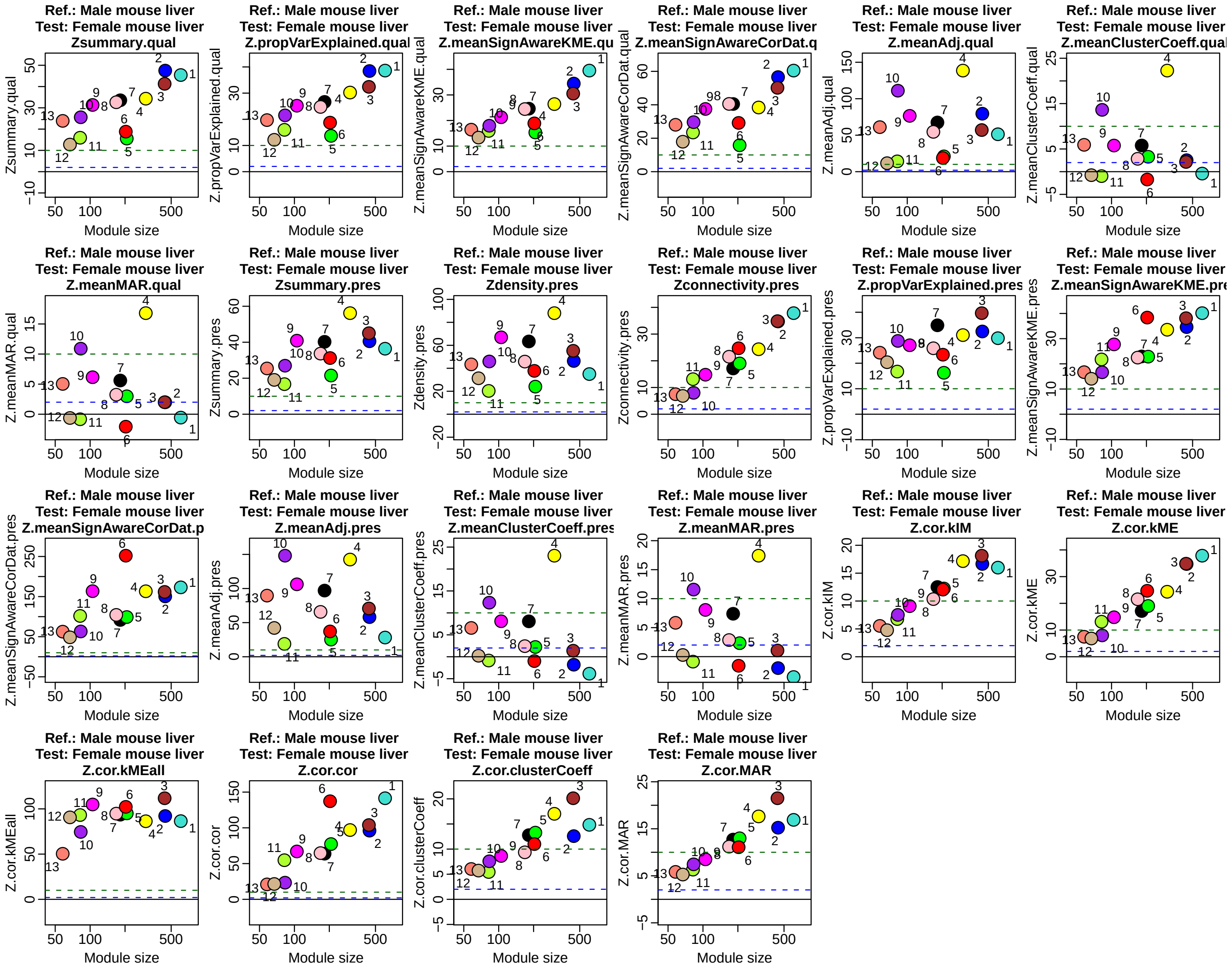

Figure 5. Module preservation of consensus modules between male and female mouse liver samples.

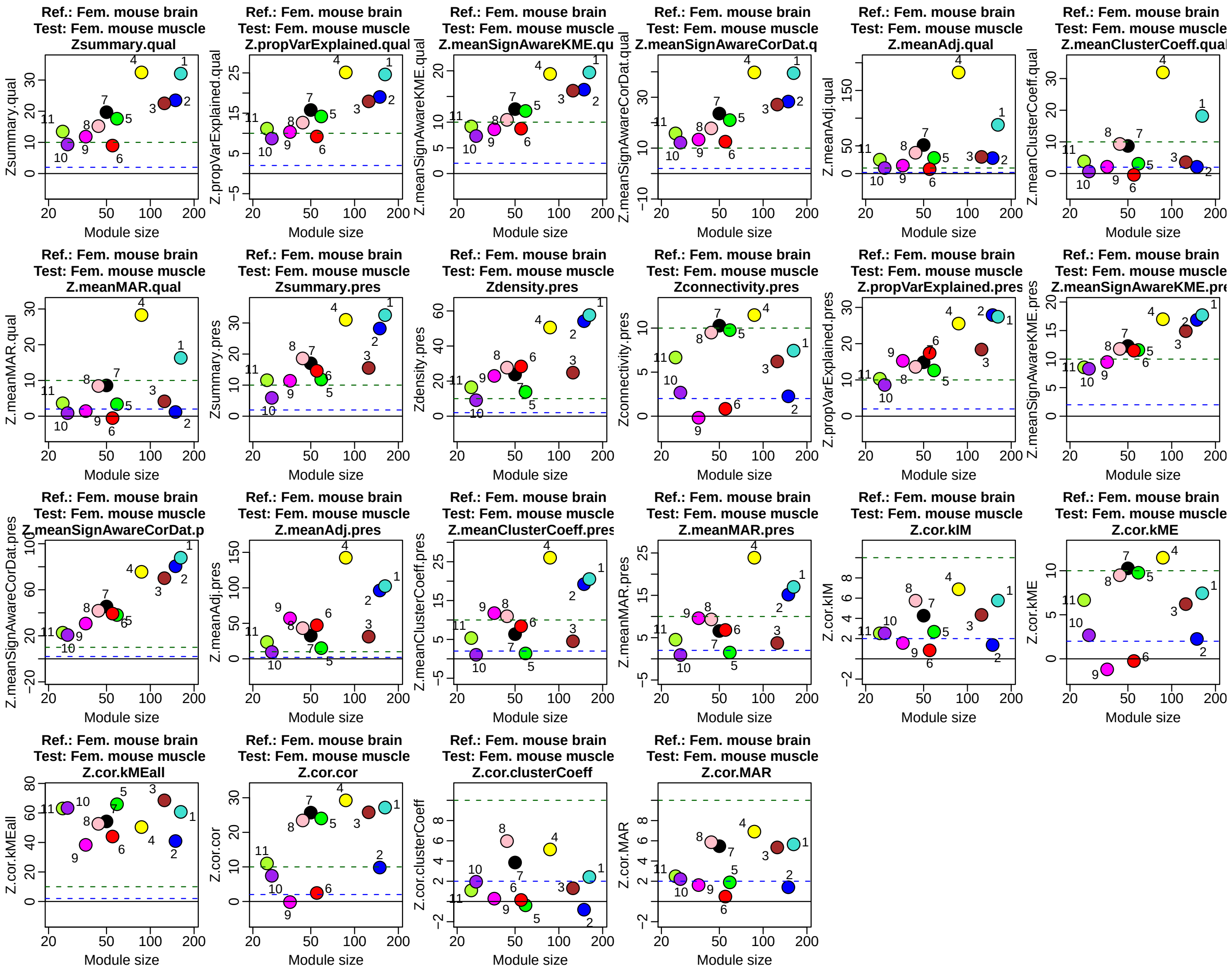

Figure 6. Module preservation of consensus modules between female brain and muscle samples.

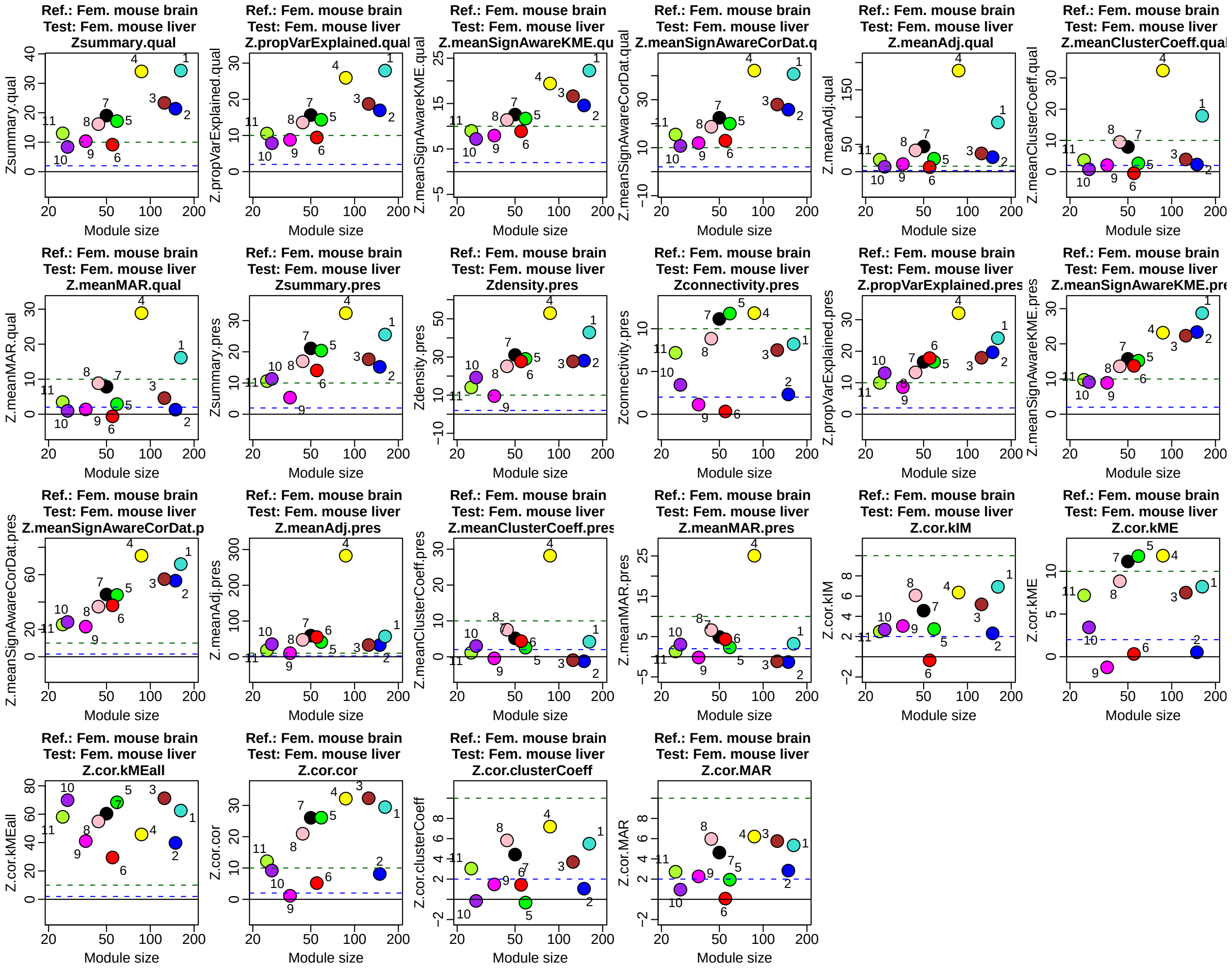

Figure 7. Module preservation of consensus modules between female brain and liver samples.

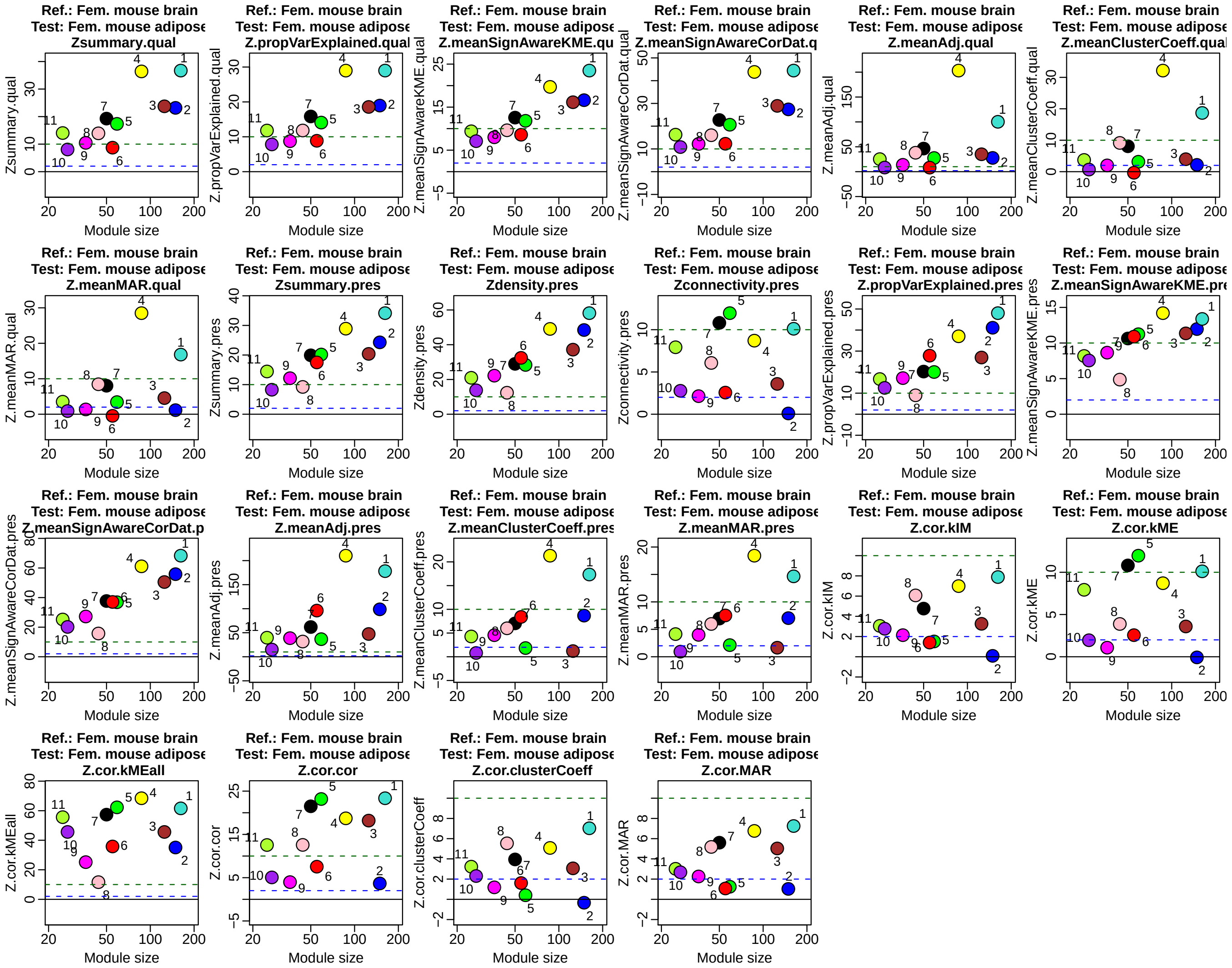

Figure 8. Module preservation of consensus modules between female brain and adipose samples.

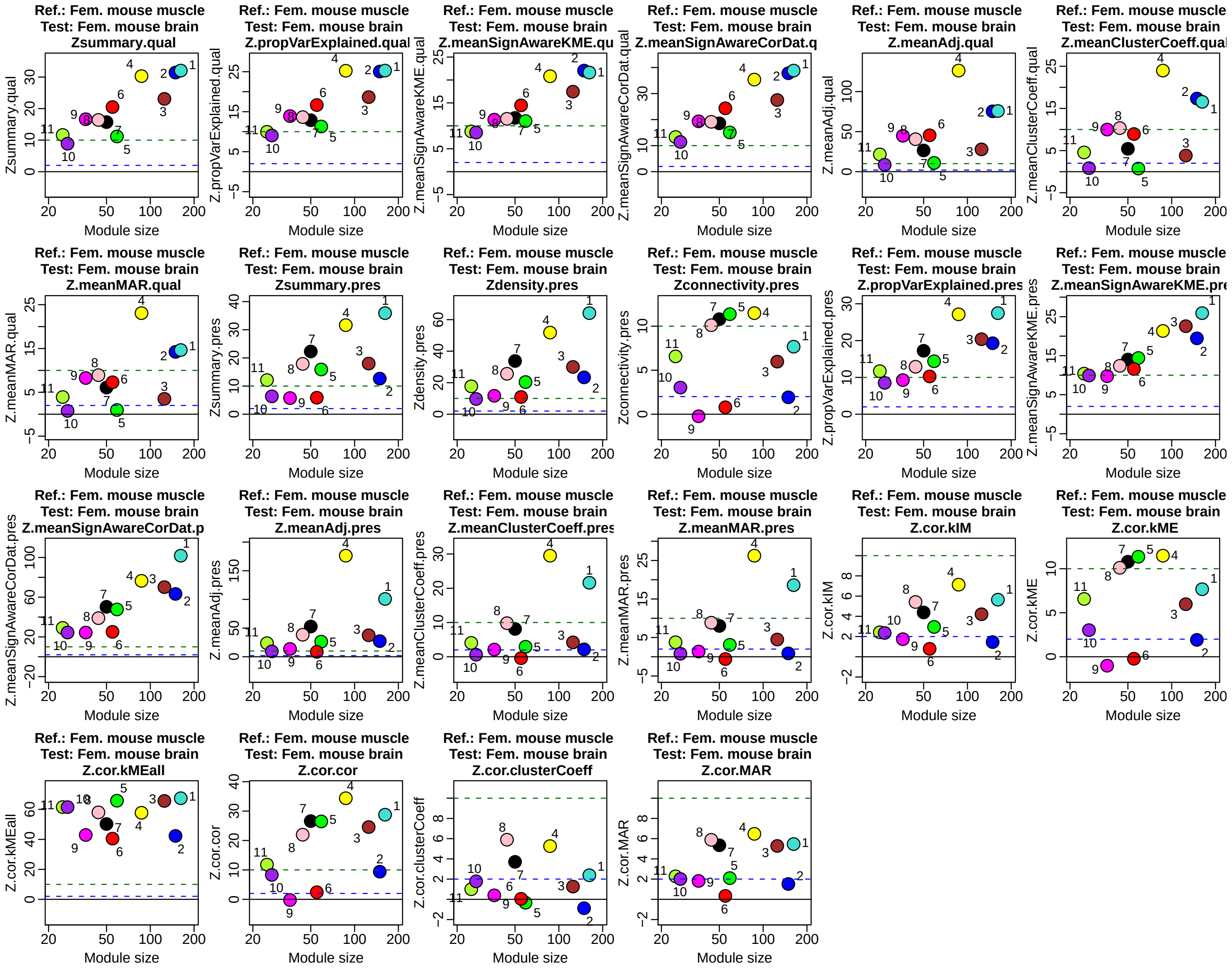

Figure 9. Module preservation of consensus modules between female muscle and brain samples.

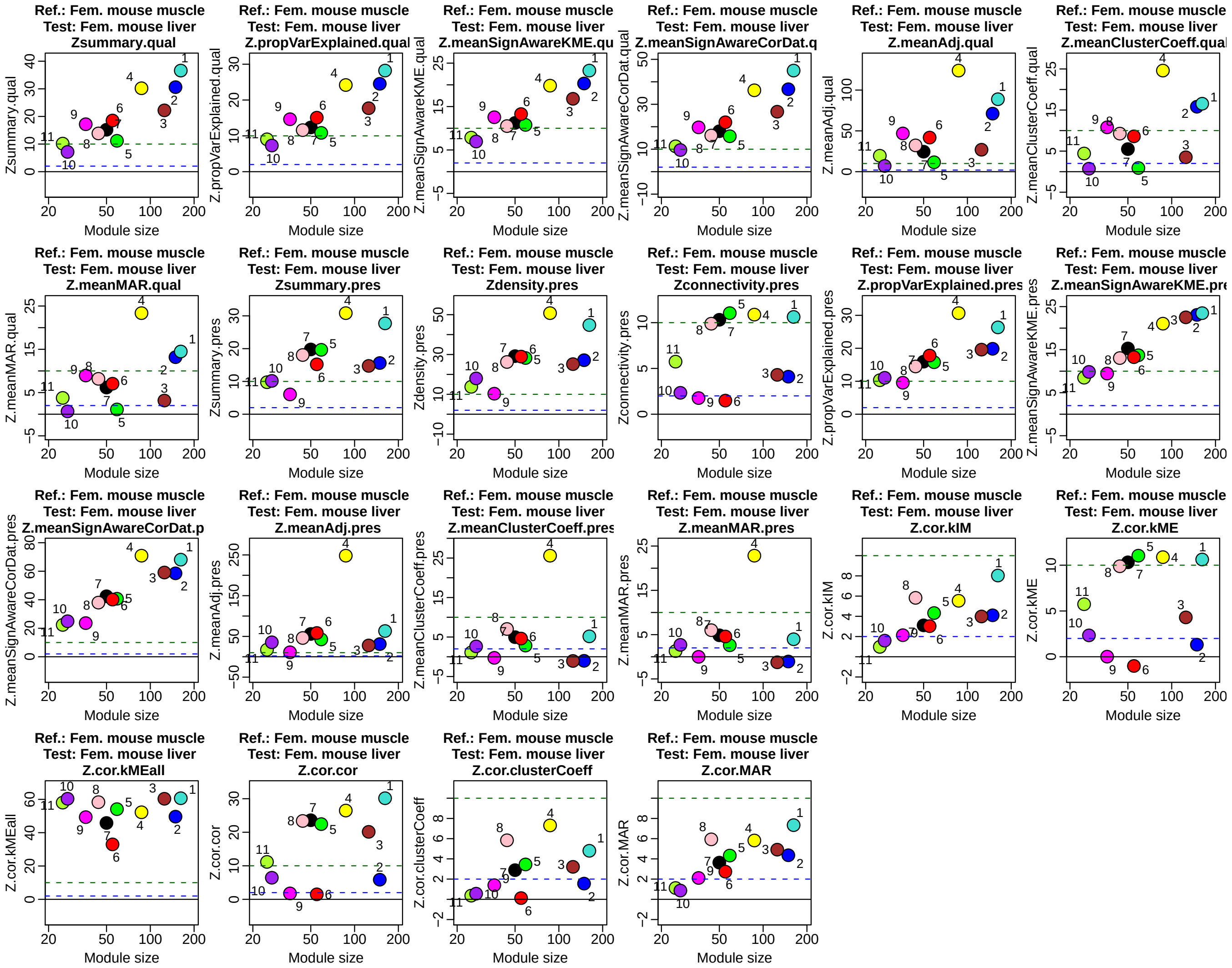

Figure 10. Module preservation of consensus modules between female muscle and liver samples.

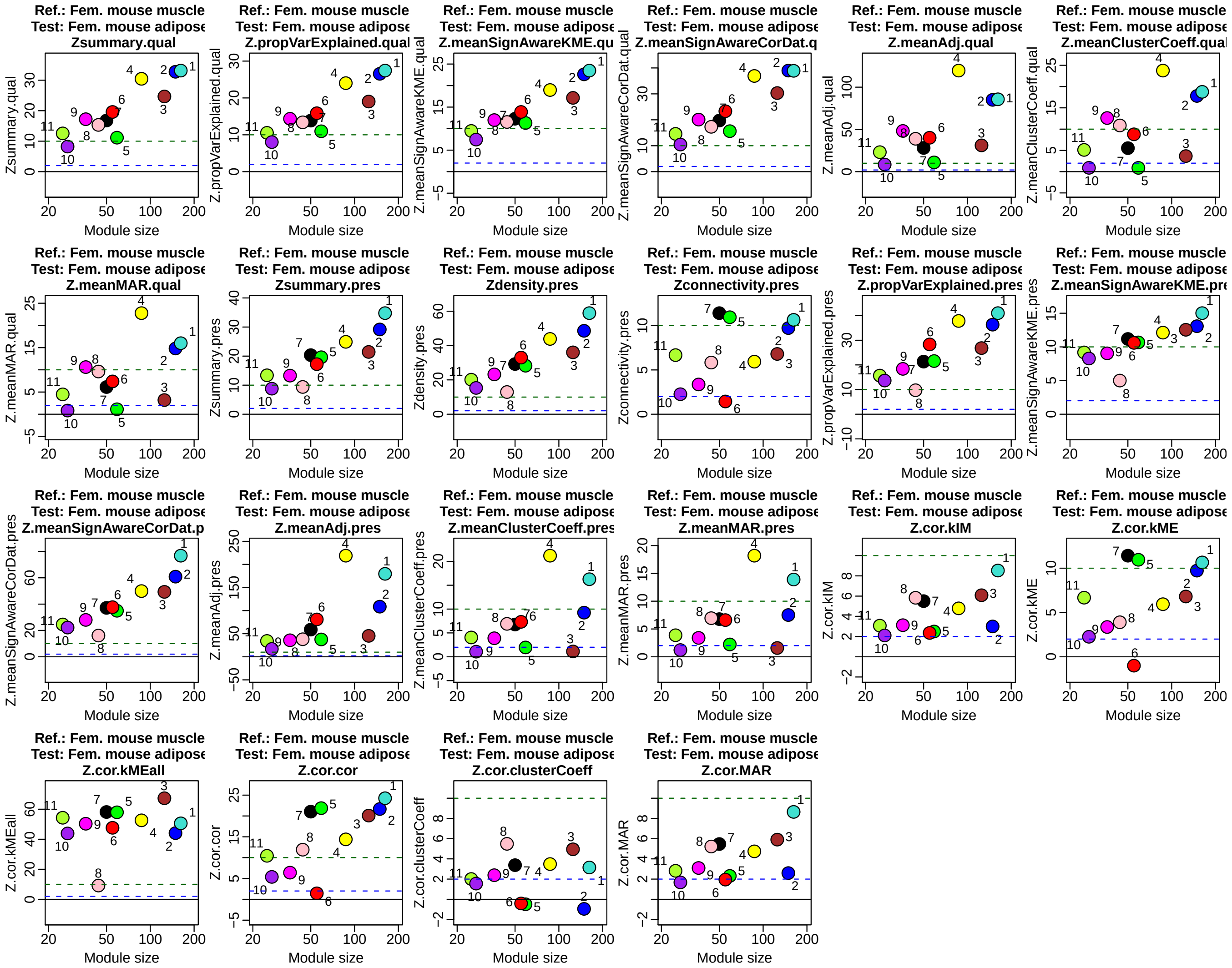

Figure 11. Module preservation of consensus modules between female muscle and adipose samples.

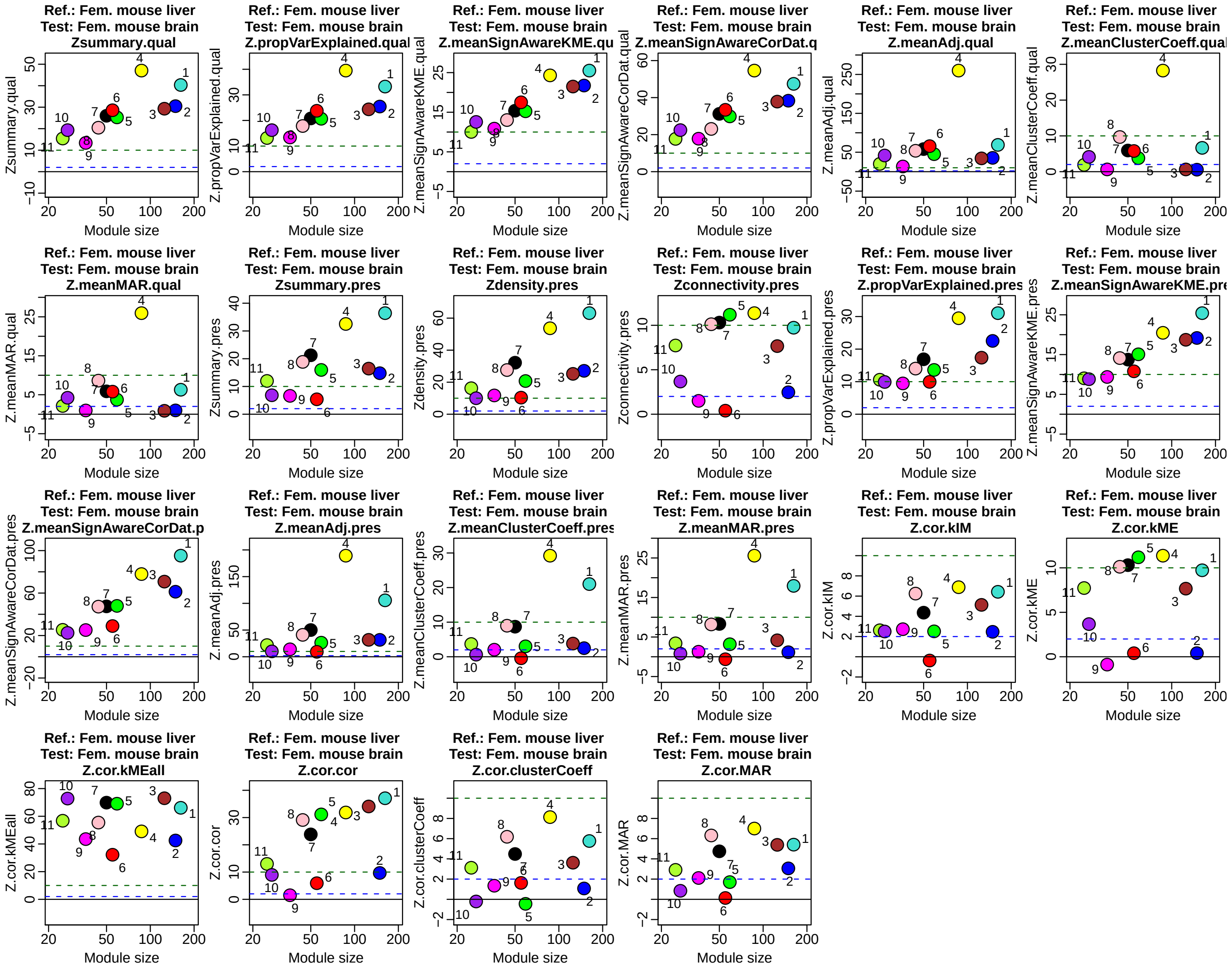

Figure 12. Module preservation of consensus modules between female liver and brain samples.

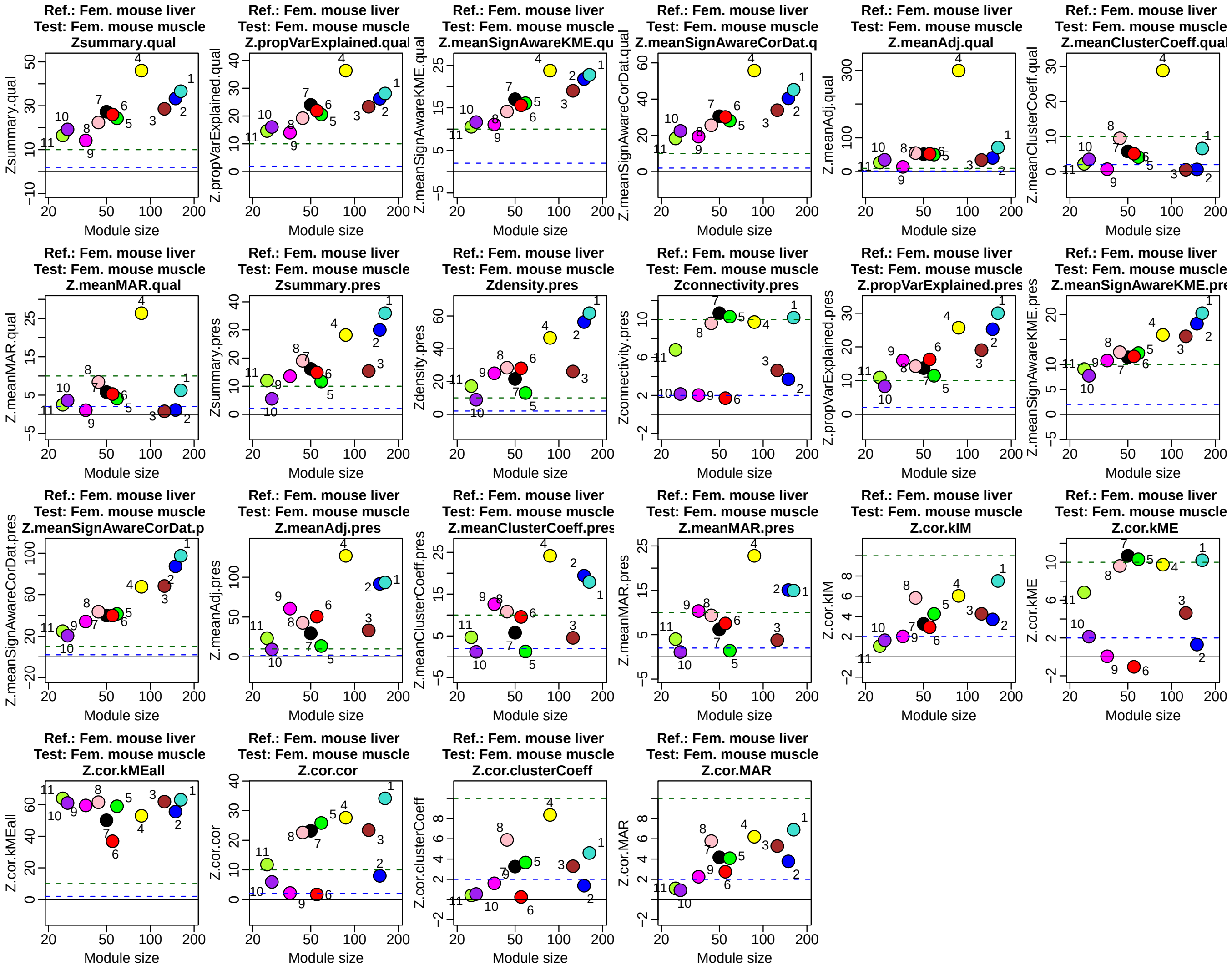

Figure 13. Module preservation of consensus modules between female liver and muscle samples.

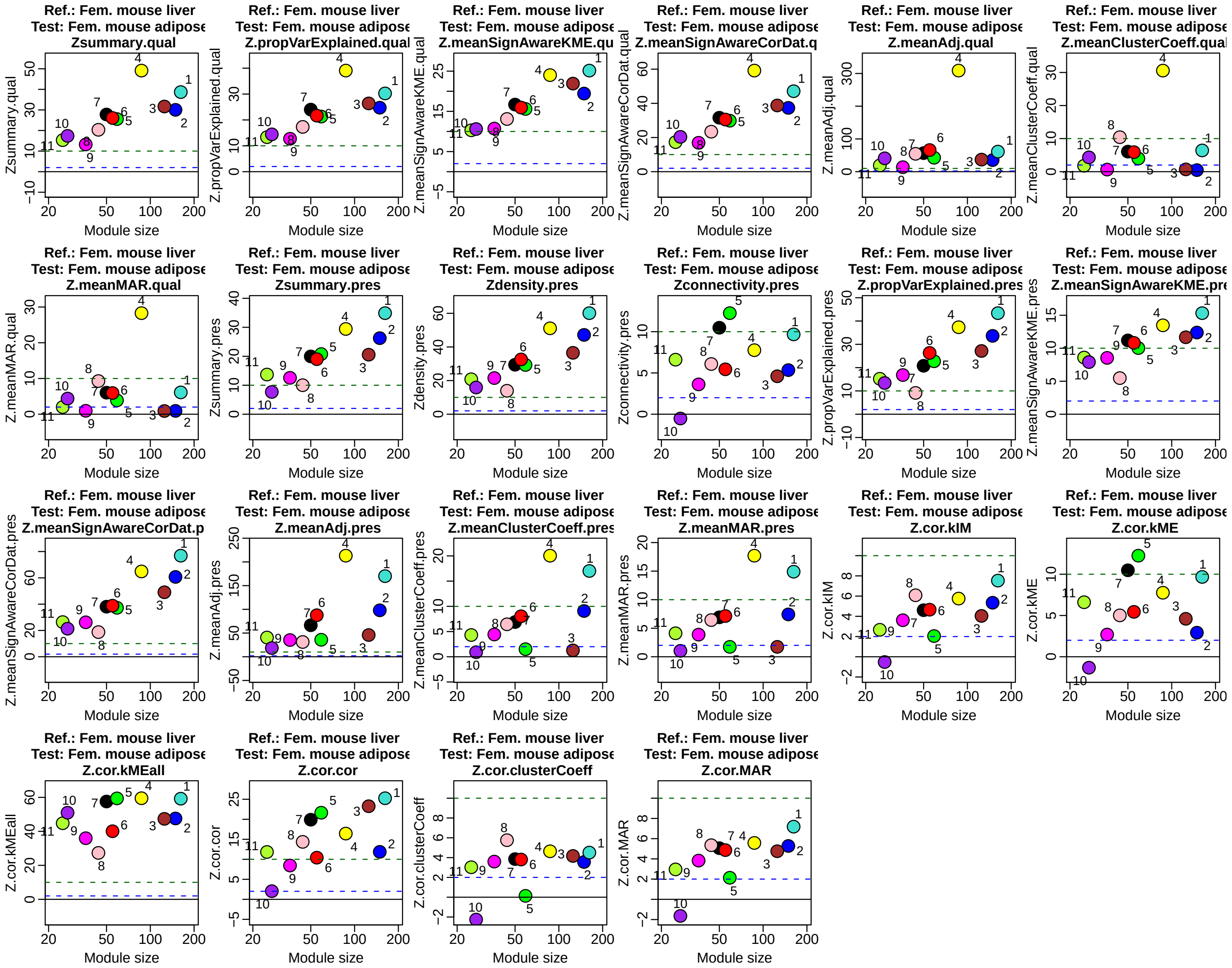

Figure 14. Module preservation of consensus modules between female liver and adipose samples.

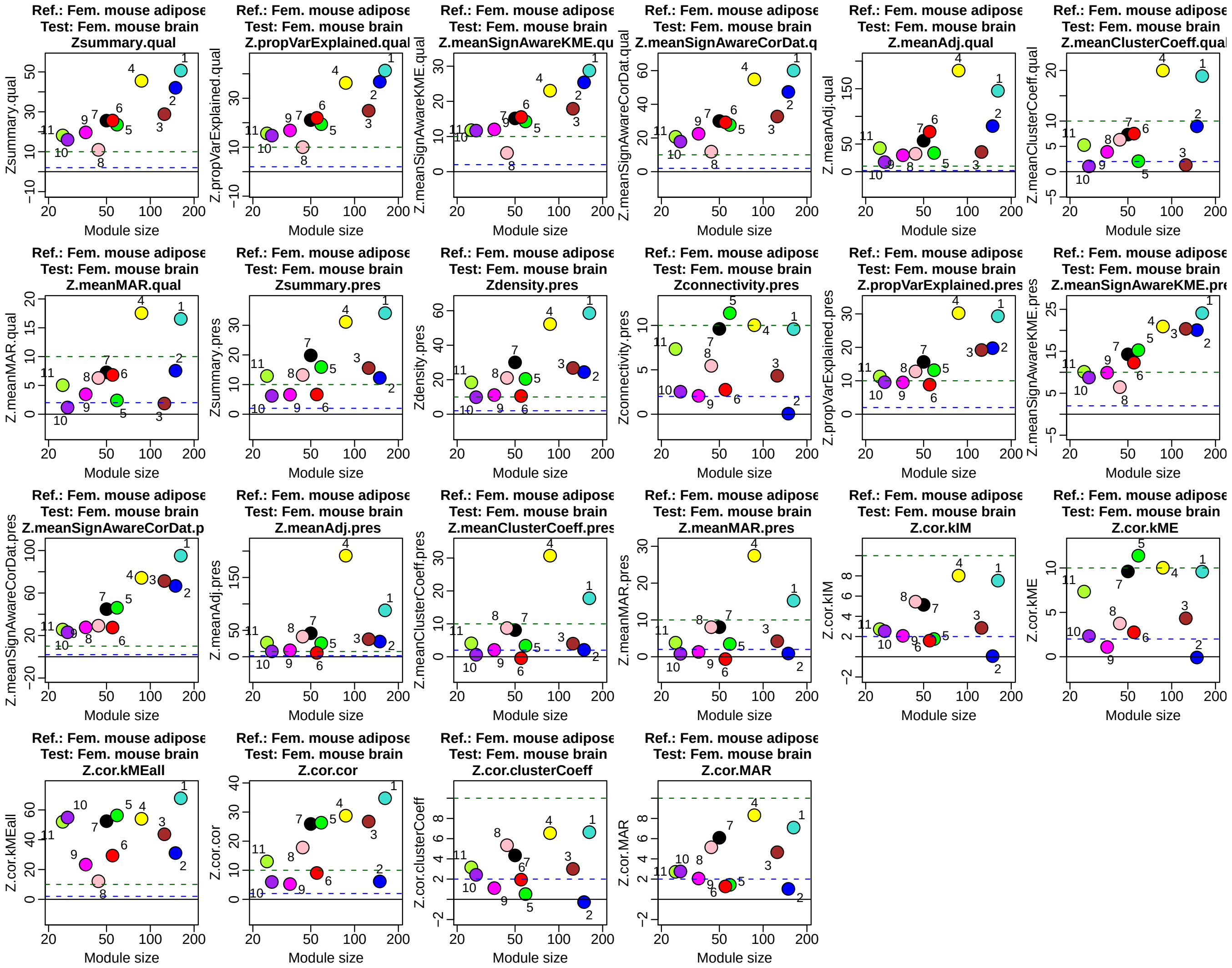

Figure 15. Module preservation of consensus modules between female adipose and brain samples.

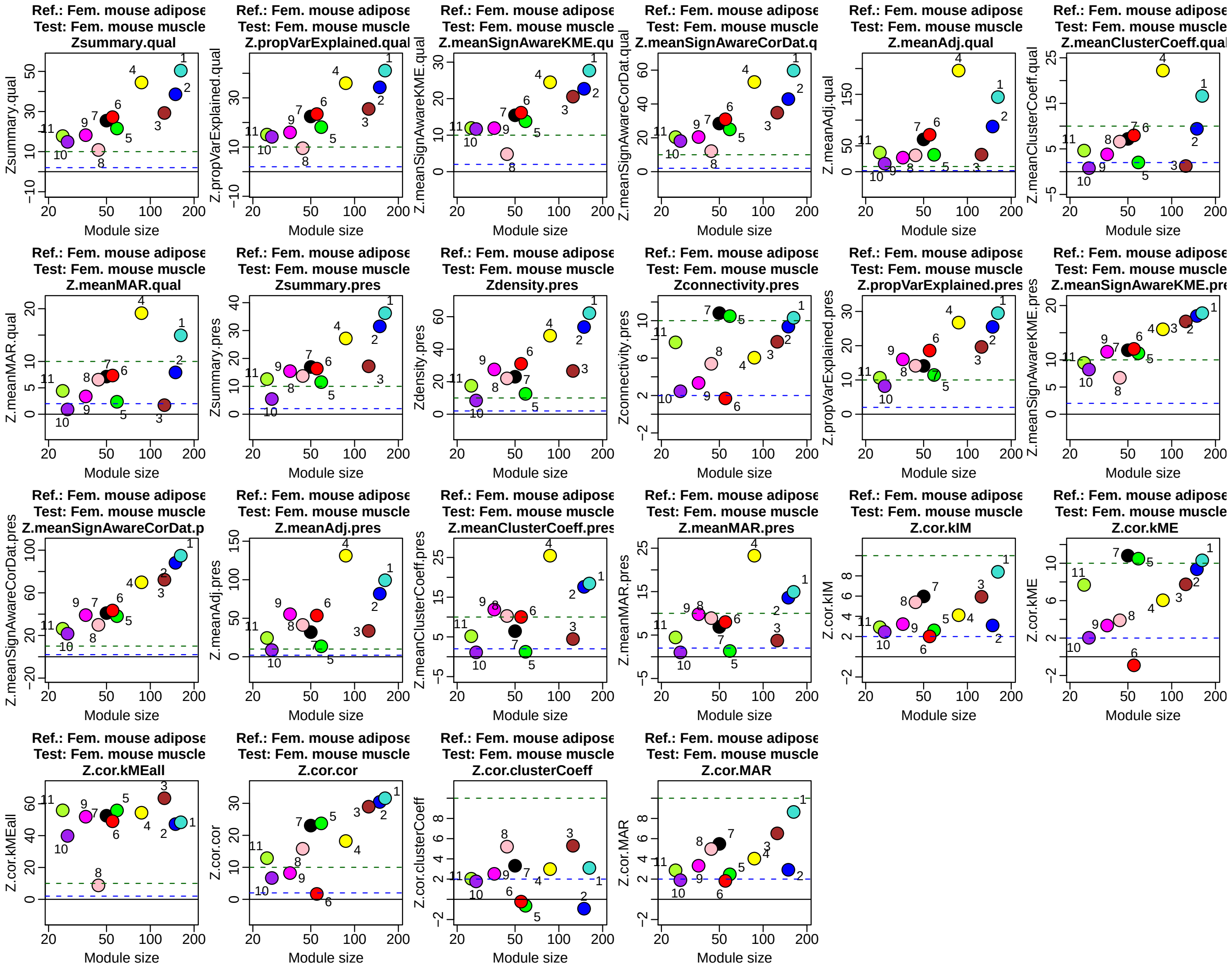

Figure 16. Module preservation of consensus modules between female adipose and muscle samples.

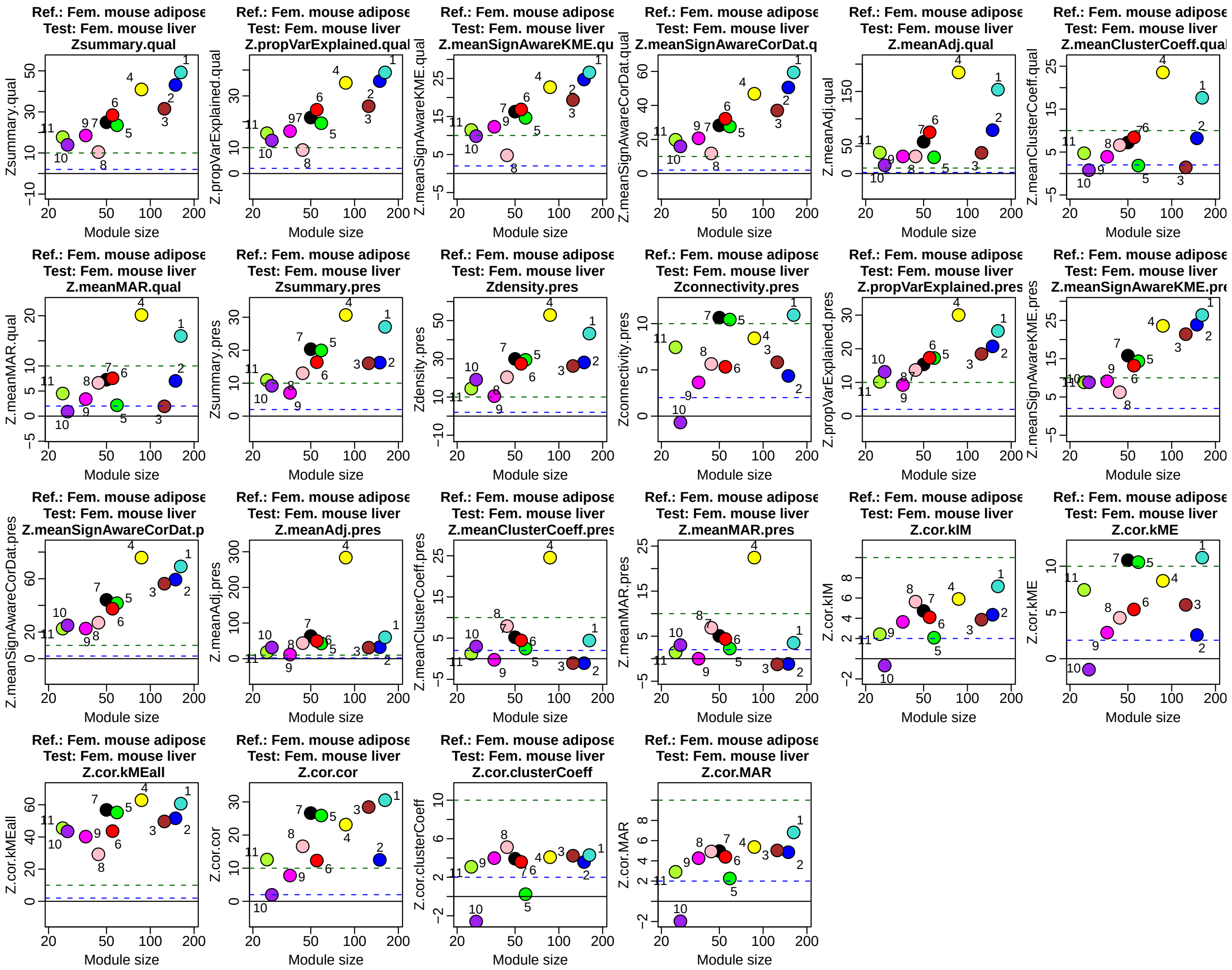

Figure 17. Module preservation of consensus modules between female adipose and liver samples.

1 Results of IGP

Here we present IGP (Figure 18) and permutation p-value (Figure 19 of consensus modules across the data sets from which the consensus modules were identified. Since consensus modules are by definition modules present in all data sets, we expect a high IGP for all modules. Overall this expectation is borne out in the results. Exceptions are modules female mouse 4-tissue consensus modules 6 and 9 (red and magenta) in the brain data that show lower IGP. These modules also exhibit lower $Z_{summary}$ preservation score.

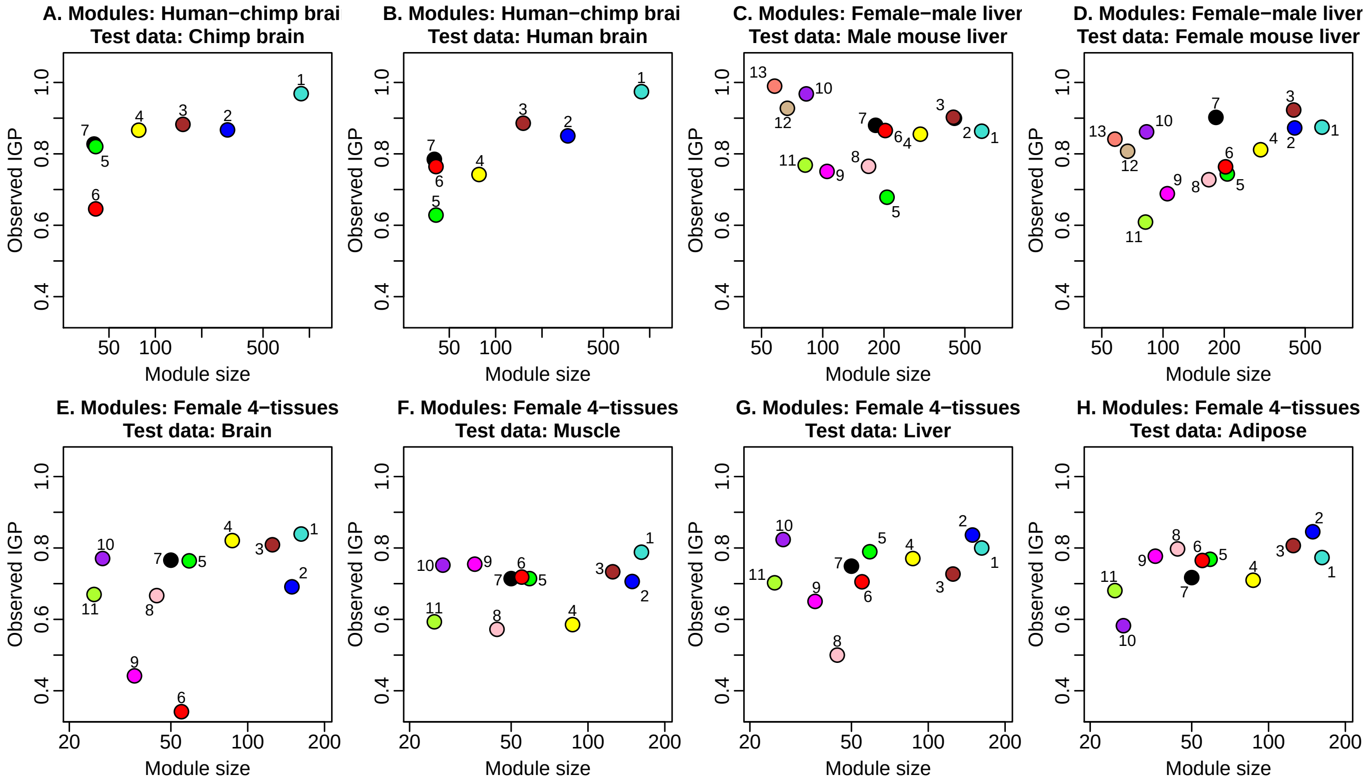

Figure 18. Observed in-group proportion of consensus modules across the data sets from which the consensus modules were identified. Modules are labeled by their labels and colors. Since consensus modules are by definition modules present in all data sets, we expect a high IGP for all modules.

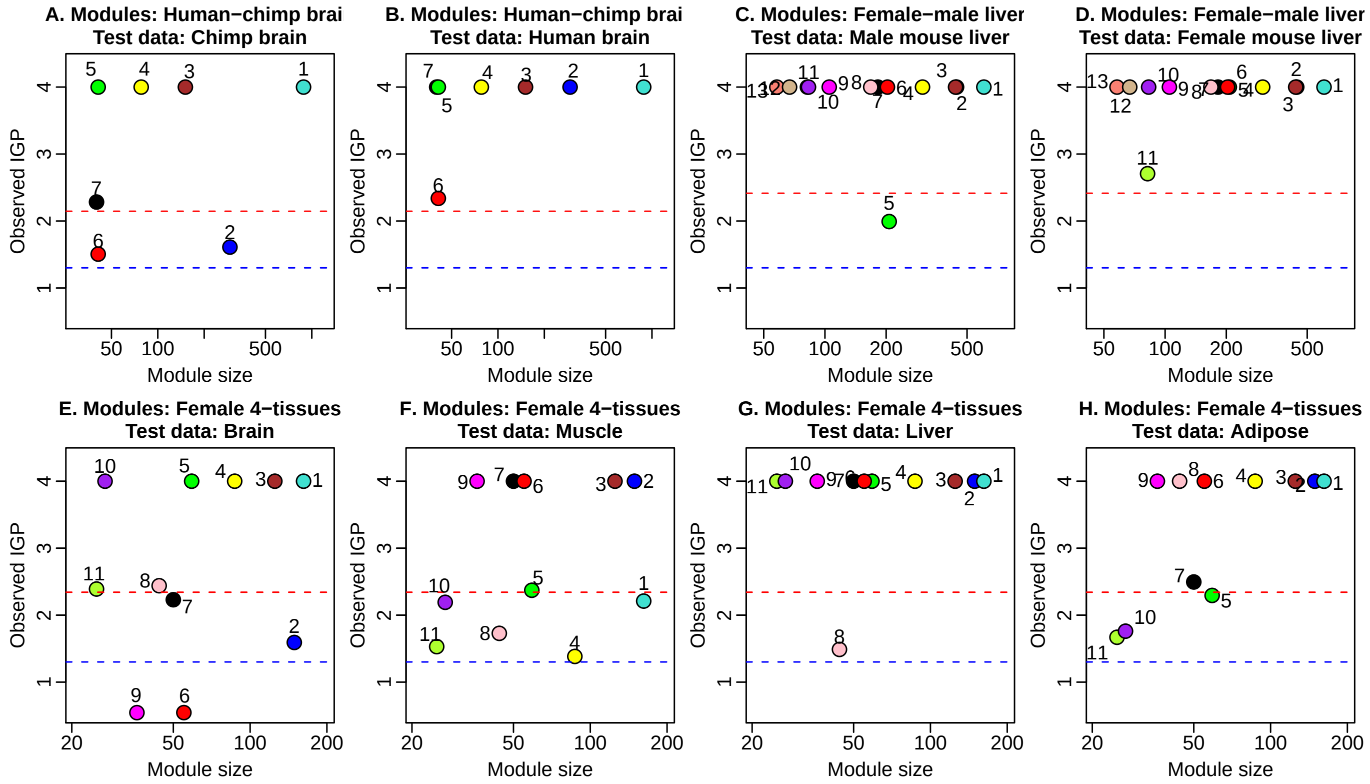

Figure 19. Permutation p-value of ofbserved IPG of consensus modules across the data sets from which the consensus modules were identified. Modules are labeled by their labels and colors.

References

1. Langfelder P, Horvath S (2007) Eigengene networks for studying the relationships between co-expression modules. BMC Systems Biology 1: 54.

2. Khaitovich P, Muetzel B, She X, Lachmann M, Hellmann I, et al. (2004) Regional Patterns of Gene Expression in Human and Chimpanzee Brains. Genome Res 14: 1462-1473.

3. Oldham M, Horvath S, Geschwind D (2006) Conservation and evolution of gene coexpression networks in human and chimpanzee brains. PNAS 103: 17973-17978.

4. Ghazalpour A, Doss S, Zhang B, Plaisier C, Wang S, et al. (2006) Integrating genetics and network analysis to characterize genes related to mouse weight. PloS Genetics 2: 8.
