# Supplementary material for: Is My Network Module Preserved and Reproducible?
Source: PLoS Comput Biol. 2011 Jan 20;7(1):e1001057. doi: 10.1371/journal.pcbi.1001057 (PMC3024255; doi:10.1371/journal.pcbi.1001057)
Supplement: Text S6 — Detailed description of the simulation study. Detailed performance analysis of the proposed module preservation statistics in seven simulation scenarios. The design and main results of the simulations are summarized in Figure 9 of the main text. (2.78 MB PDF) [file pcbi.1001057.s011.pdf]

Supplementary text S6
Comparison studies on simulated data

Peter Langfelder, Rui Luo, Michael C. Oldham, and Steve Horvath*
*Corresponding author: shorvath@mednet.ucla.edu

1 Overview

In this document we illustrate the module preservation statistics on simulated data. We describe the simulation method and seven simulation studies in more detail than in the main text. The design and main results of the simulations are summarized in Figure 8 of the main article which we reproduce here for convenience in Figure 1.

A complete table of results can be found in the accompanying Supplementary Table S5. This is a flat comma separated value (CSV) text file that can be viewed in most standard spreadsheet software such as MS Excel or OpenOffice Calc. The columns indicate the simulation, module label, module size, observed preservation statistics, and their Z scores, for each module in each of the simulated comparisons. R software tutorials describing the results of additional simulation studies can be found on our web page.

2 Data simulation

We use the data simulation functions in the WGCNA package [2] to simulate clusters of correlated genes. In most of our simulations, these clusters are the modules whose preservation we study, but in pathway simulations the modules are different from the clusters. Each cluster is simulated around a "seed eigennode", which is a randomly chosen profile. In the simulations presented here, seed eigennodes for different clusters are simulated independently, but they can also be simulated to exhibit correlations (in empirical data we often observe that eigennodes of different clusters are correlated). The components of each seed eigengene vector E^a_q, $a = 1, 2, ..., m$, $m = 100$ are drawn from normal distribution with mean 0 and variance 1 (denoted $N(0, 1)$). The cluster genes, labeled by index i, $i = 1, 2, ..., n_q$, are then simulated as

$$X^a_{i(q)} = r_i E^a_q + \sqrt{(1 - r_i^2)}\epsilon^a_i \,, \tag{1}$$

where the "noise" components ϵ^a_i are chosen independently from $N(0, 1)$, and the coefficients r_i are uniformly spaced between $r_{\min}$ and $r_{\max}$. To simulate well-defined (tightly coexpressed) clusters we use $r_{\min} = 0.3$ and $r_{\max} = 1$. Lower values can be used to simulate clusters with weak co-expression. Most genes outside of clusters are simulated with independent expression values drawn from $N(0, 1)$, whereas a small number are simulated as "near-cluster genes" according to Equation 1, but with r_i ranging from 0 to $r_{\min}$.

3 Simulation 1: Weak module preservation

Here we present an example of module preservation where the signal is weak and standard cross-tabulation methods fail to detect it reliably. We simulate 5000 genes in 100 samples. In the reference data set we simulate 20 modules with sizes around 200 genes, more precisely from 185 to 220 genes. In the test set, 10 of the 20 modules are simulated as preserved, that is according to Equation 1, but weakly: $r_{\min} = 0.05$

and $r_{\max} = 0.35$. The other 10 are simulated as not preserved at all, that is genes in these modules are simulated with independent expression values drawn from $N(0, 1)$.

Using network construction and module detection methods implemented in the WGCNA package, we constructed network modules in the reference data set (Figure 2). The identified modules show excellent agreement with the simulated modules. On the other hand, in the test set hierarchical clustering did not identify any network modules. To be able to study the performance of cross-tabulation methods, we used Partitioning Around Medoids (PAM) [4] with number of clusters equal 20 and distance equal to 1 minus the network adjacency. With this input, PAM will partition the simulated genes into 20 clusters irrespective of how weak the signal is.

We used the two data sets as input to our network-based module preservation calculations, and also to the `clusterRepro` function in the eponymous package described in [1]. The `clusterRepro` function calculates the In-Group Proportion (IGP) in the test set as a measure of the preservation of clusters identified originally in the reference data set. Higher values of IGP indicate that the corresponding reference clusters are better preserved (reproduced) in the test data. The significance of the observed IGP is quantified using a permutation procedure leading to a p-value.

The simulation results are summarized in Figure 3. The results indicate that network-based module preservation indices are capable of correctly identifying preserved modules (see panels A and B of Figure 3). Cross-tabulation methods (panels C and D) clearly depend on the clustering method used in the test set. For example, hierarchical clustering and branch cutting identified no modules in the test set, and one would conclude that none of the modules are preserved. If one uses PAM to partition the test set into clusters, neither co-clustering nor Bonferroni-corrected Fisher exact test p-value can reliably distinguish preserved from non-preserved modules. A similar conclusion holds for the IGP method implemented in `clusterRepro` (panels E and F). In this example, `clusterRepro` performs worse at separating the preserved from non-preserved modules than cross-tabulation.

Using a threshold of 0.05 for Bonferroni-corrected p-values (green line in all p-value plots in Figure 3), network-based module preservation correctly identifies all preserved and non-preserved modules. Cross-tabulation achieves perfect accuracy (no false positives), but its sensitivity is only 50% (only 5 of the 10 preserved modules are identified as preserved). The `clusterRepro` also achieves perfect accuracy, but with very low sensitivity of 10% (only 1 of 10 preserved modules was identified as preserved).

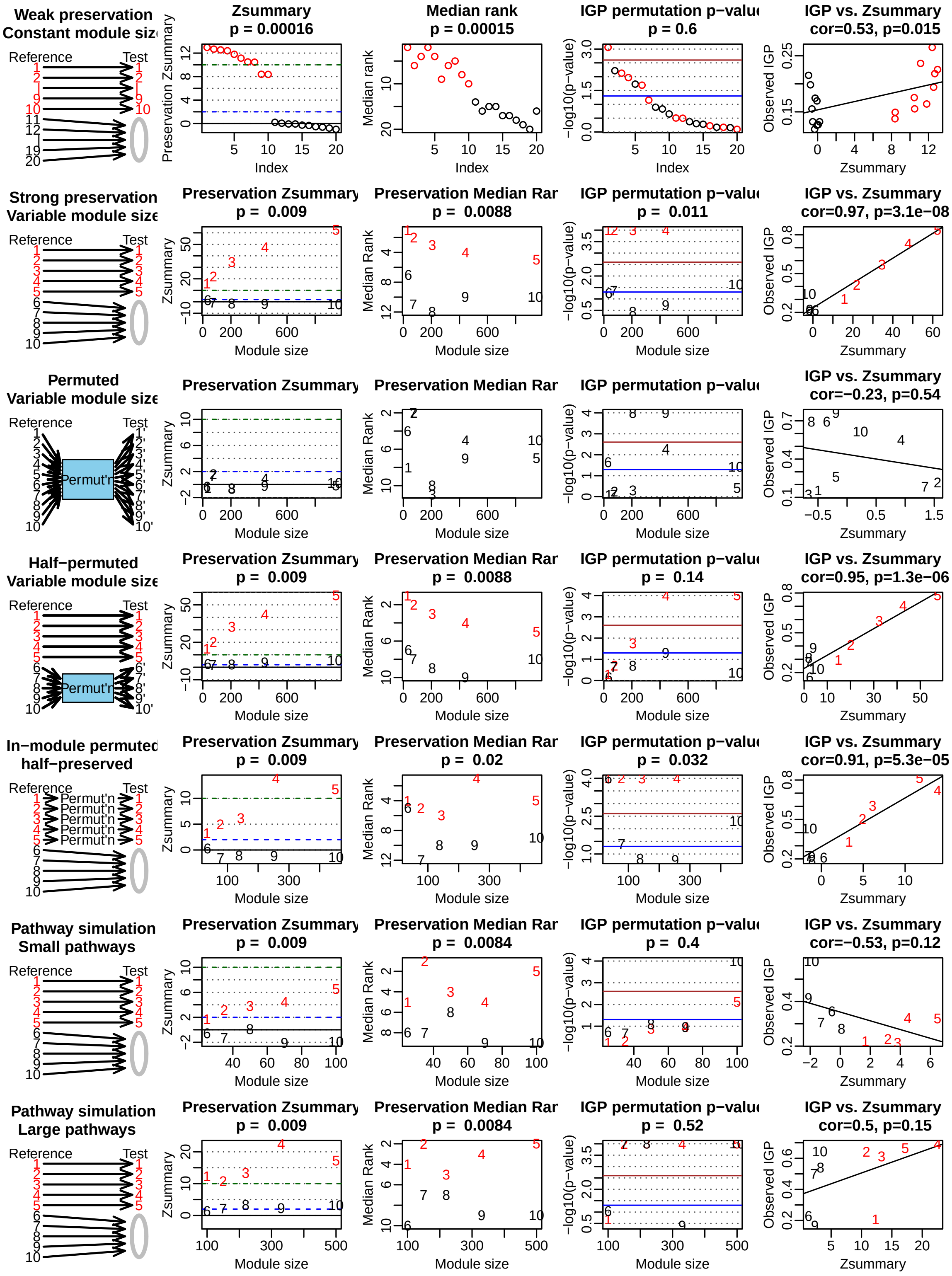

Figure 1. Design and main results of simulation studies of module preservation.

Figure 1. Design and main results of simulation studies of module preservation. For convenience, we reproduce this figure from the main article here. The first column shows cartoon views of our four simulation studies. In each simulation we simulate a reference and a test data set, each with its own set of modules labeled 1–10 or 1–20. Some of the modules are simulated as preserved, that is, present in both the reference and the test sets in the corresponding simulation. The preserved modules are marked in red color, and an arrow connects the preserved module in the reference and test data sets. The grey 0 represents genes whose profiles are simulates to be independent (that is, without any correlation structure). The second and third columns show the network preservation summary statistics $Z_{summary}$ and *medianRank* in each study. Preserved modules are denoted by red color, and non-preserved by black color. The blue and green lines show the thresholds of $Z_{summary} = 2$ and $Z_{summary} = 10$, respectively. In each title we also give the Kruskal-Wallis rank-sum test p-value. The summary statistics separate preserved and non-preserved modules very well, in most cases $Z_{summary} > 10$ for preserved modules and $Z_{summary} < 2$ for non-preserved modules. The fourth column shows the permutation p-values of IGP obtained by the R package `clusterRepro`. The blue and brown lines show p-value thresholds of 0.05 and its Bonferroni correction, respectively. The IGP permutation p-value is less seccessful than our summary statistics. The fifth and last column shows scatterplots of observed IGP vs. $Z_{summary}$. We observe that whenever the modules correspond to clusters and the strength of preservation varies significantly, IGP and $Z_{summary}$ tend to be highly correlated.

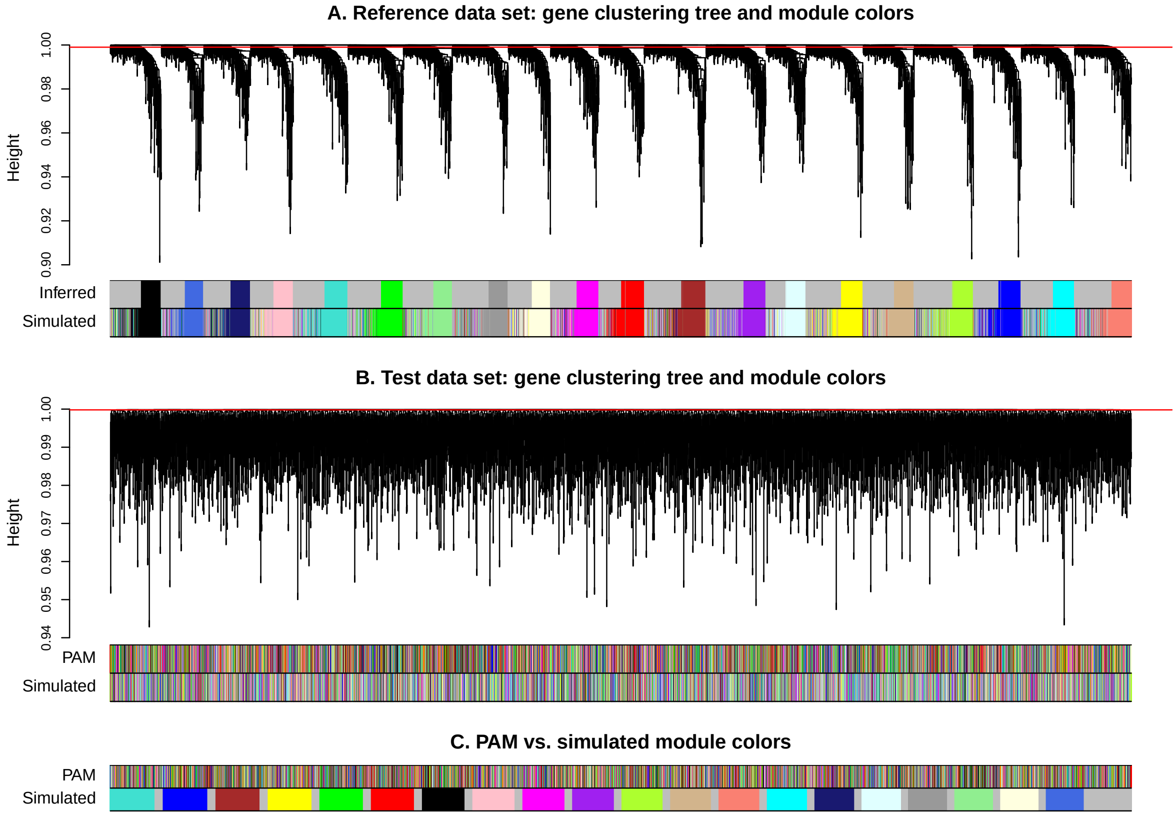

Figure 2. Simulated data where cross-tabulation methods fail due to weak signal A. Hierarchical clustering dendrogram and modules (labeled by colors) in the reference data set. Inferred and simulated modules show excellent agreement since the simulated modules are well-defined. B. Hierarchical clustering dendrogram and modules (labeled by colors) in the test data set. The hierarchical clustering dendrogram shows no branches that could be identified as modules. Because the modules have been simulated with very weak correlations, the simulated colors, shown below the dendrogram together with clusters identified by PAM, appear to have no relationship to the structure of the dendrogram. In other words, hierarchical clustering fails to identify the simulated modules in this scenario. C. Correspondence between simulated module labels and PAM. Again, because the modules have been simulated with weak correlations, there is no apparent correspondence between the simulated module colors and the module colors inferred by PAM.

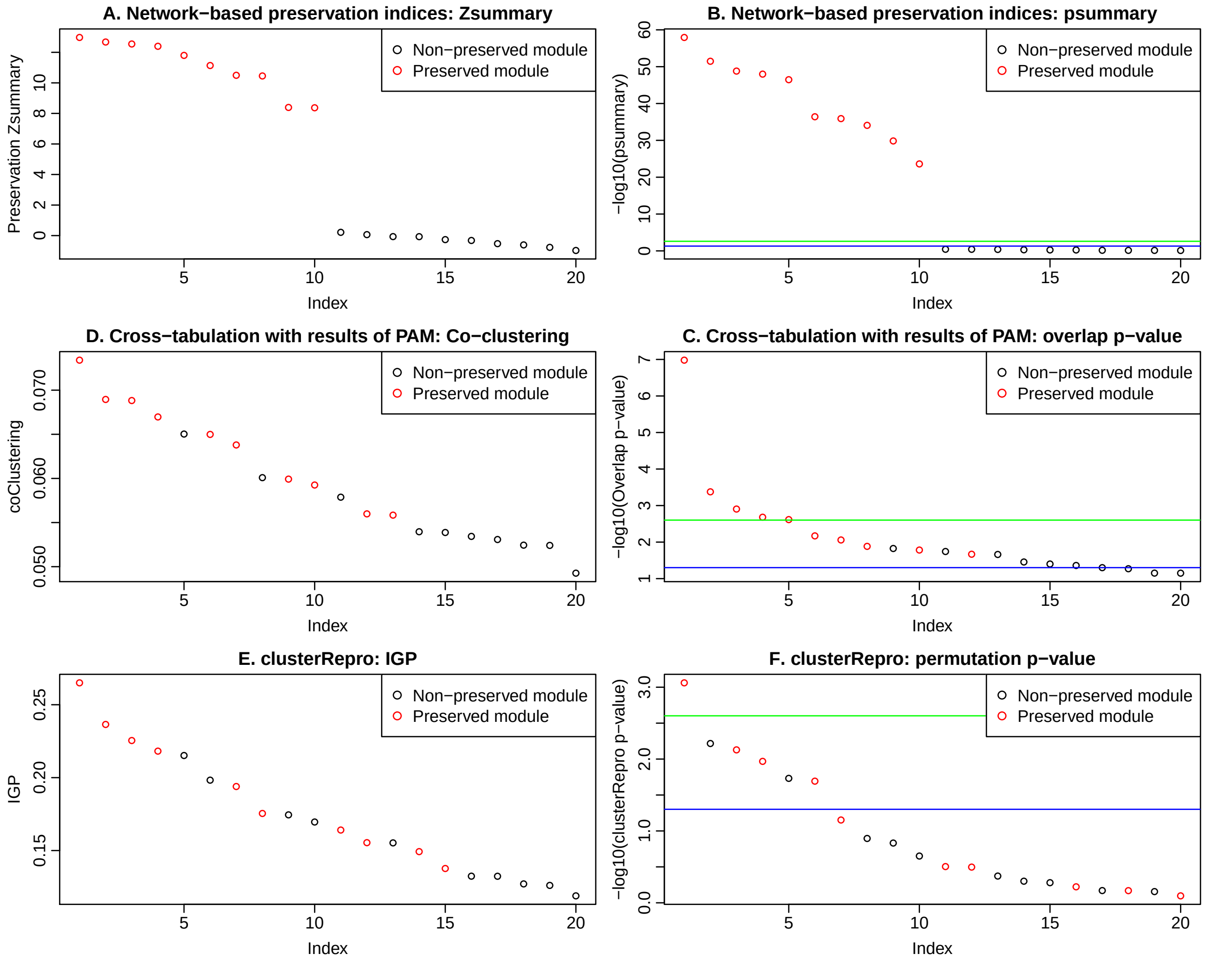

Figure 3. Success of various methods in distinguishing preserved and non-preserved modules. Selected preservation measures calculated by three methods of quantifying module preservation. The top row shows the results of network module preservation indices described in this work. Both Zsummary (top left) and psummary (top right) clearly distinguish preserved modules (red circles) from the non-preserved modules (black circles). In the p-value plot, the blue and green lines denote significance thresholds 0.05 and 0.0025 (0.05 Bonferroni corrected for 20 modules), respectively. The middle row shows results of cross-tabulating modules. The middle left panel shows the co-clustering coefficient, while the right panel shows the Fisher exact test p-values. Cross-tabulation does not succeed in separating the preserved and non-preserved modules with perfect accuracy. Further, taking a threshold of 0.05 after Bonferroni correction (green line), the method indicates that only 5 modules are preserved. The bottom two panels show results of `clusterRepro`, IGP (bottom left panel) and the corresponding permutation p-value. The method does not succeed in separating the preserved and non-preserved modules, and identifies only 1 module as preserved after Bonferroni correction (green line).

Lastly, for completeness we present the detailed results of all preservation Z scores in Figure 4. In this application the density statistics perform better than connectivity statistics at distinguishing preserved and non-preserved modules. Accuracy statistics perform much worse, and separability fails completely.

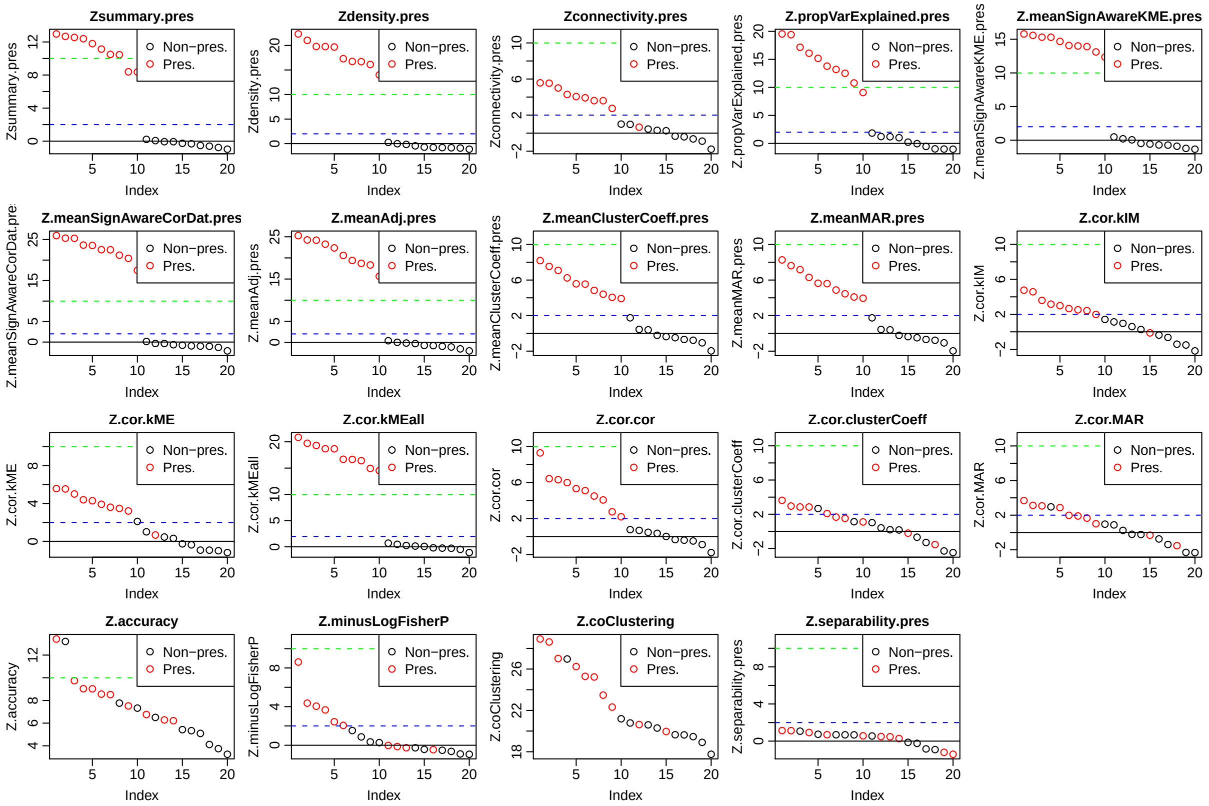

Figure 4. Success of our Z statistics at distinguishing preserved and non-preserved modules. This figure contains detailed results of all preservation statistics described here. Preserved and non-preserved modules are denoted by red and black circles, respectively. The blue and green dashed lines denote Z thresholds of 2 and 10, respectively. In each plot, modules are ordered by the corresponding Z statistic. This figure shows that in this application the density statistics are more successful than connectivity statistics. Accuracy statistics perform worse than either density or connectivity statistics, and separability fails completely.

4 Simulations 2–5: Strong module preservation

Here we describe four different simulation scenarios in which modules are either strongly preserved or not preserved at all. In these simulations modules correspond to clusters. We simulate two data sets (reference and test), each with $m = 100$ samples and approximately 5000 nodes. In the reference data set, most of the nodes are simulated to belong to one of 10 modules whose sizes range from 50 nodes to 1000 nodes, and about 1100 nodes are simulated to be outside of the modules.

The modules in the reference data set are labeled 1 through 10, while the label 0 is reserved for unassigned nodes. We simulate four different test data sets, illustrated in the first column in Figure 10 of the main text, reproduced in Figure 1 below for convenience. In the first test data set (Half preserved), reference modules 1 through 5 are preserved, while nodes belonging to reference modules 6 through

10 are simulated with completely independent expression profiles, that is modules 6 through 10 are not preserved. In the second test data set (Permuted) we simulate a similar set of 10 modules as in the reference data set, but node labels are randomly permuted. Thus, although the test data set also contains 10 modules, none of the reference modules are preserved. In the third test data set (Half-permuted) we simulate 10 modules. Test modules 1 through 5 correspond to reference modules 1 through 5; nodes that belong to reference modules 6 through 10 also belong to test modules 6' through 10', but their module memberships are randomly permuted. Thus, only reference modules 1 through 5 are preserved in test set 3. The difference between test sets 1 and 3 is that nodes that belong to non-preserved modules are simulated completely independent in test set 1, while in test set 3 they are simulated in 5 modules whose membership is randomly permuted. Lastly, in the In-module permuted, half-preserved simulation, in the test set reference modules 1 through 5 are preserved, while nodes belonging to reference modules 6 through 10 are simulated with completely independent expression profiles, that is modules 6 through 10 are not preserved. Additionally, genes belonging to each of the 5 preserved modules in the test set are randomly permuted (within each module). Thus, in this simulation the clusters are preserved but the connectivity relationships are randomly permuted.

Results of the simulation studies confirm that for preserved modules the summary preservation Z statistic is high, $Z_{summary} > 10$ (second row of Figure 1). On the other hand, for non-preserved modules the summary preservation Z statistic is low, $Z_{summary} \leq 2$.

Figures 5–7 show all Z statistics in simulation studies 2–4. In each figure, we plot the module quality and preservation Z statistics (y-axis) of density and network structure preservation statistics as a function of module size (x-axis). Modules are labeled by their numeric labels; modules simulated to be preserved between the reference and test data are indicated in red, while the ones simulated as non-preserved are indicated in black. The dashed blue and green lines indicate the thresholds $Z = 2$ and $Z = 10$, respectively.

We note that accuracy statistics fail to distinguish preserved and non-preserved modules in the "Half-permuted" simulation study where half of the modules are preserved and the other half have their labels randomly permuted. This is due to the fact that since only half of the modules are randomly permuted, the probability of a significant overlap is higher than would be expect by the null hypothesis tested by these statistics, namely that the module labels are random. We note that the density preservation statistics also exhibit a similar but weaker tendency of relatively high preservation statistics for non-preserved modules. The connectivity statistics are the most reliable group in this simulation study. The summary Z statistic indicates that the two largest of the permuted modules are weakly to moderately preserved. However, all preserved modules have much higher summary Z statistics. Hence, in this sense, the summary statistic still succeeds in separating preserved and non-preserved modules.

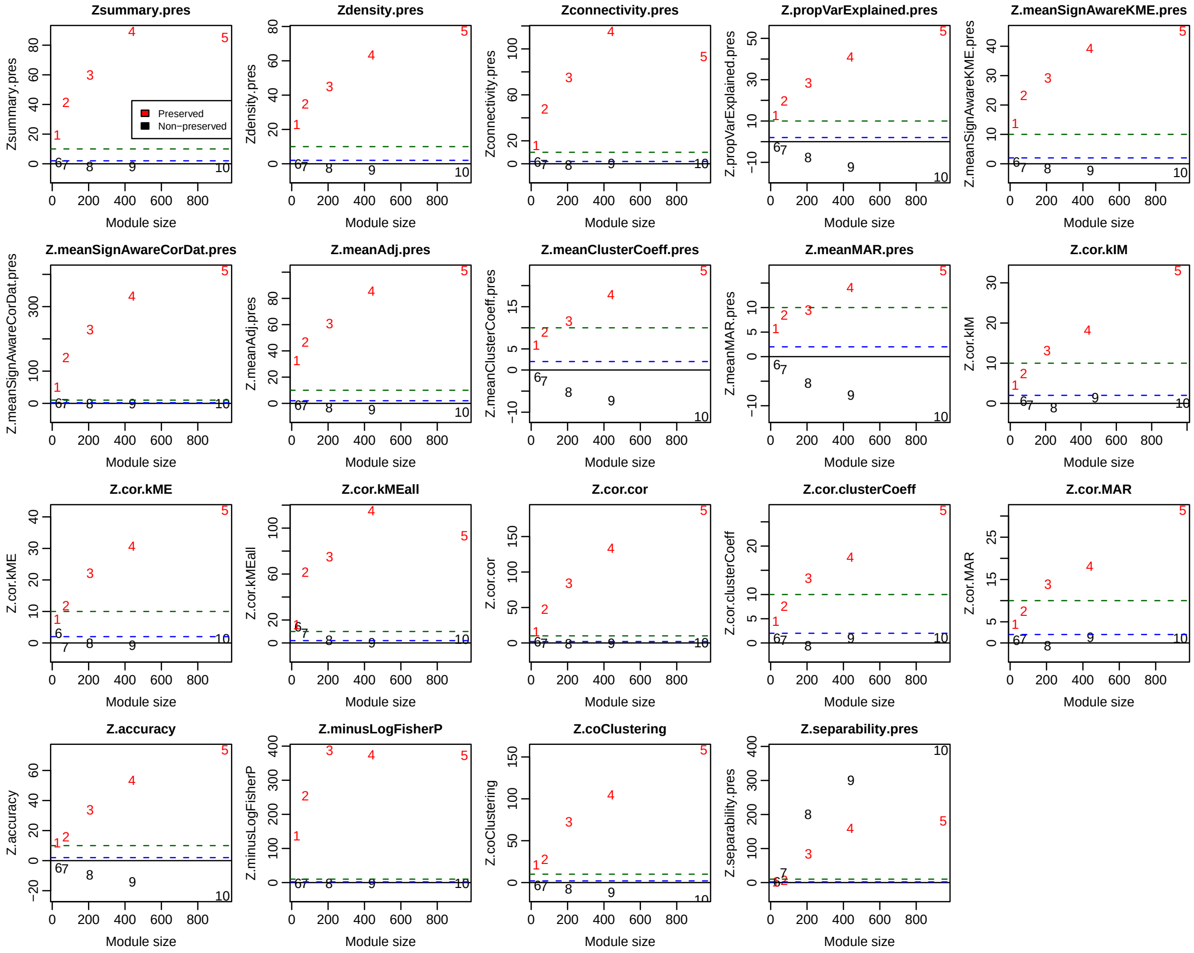

Figure 5. Module preservation Z statistics in the "Strong preservation" simulation as a function of module size. Modules are labeled by their numeric labels; modules simulated to be preserved between the reference and test data are indicated in red, while the ones simulated as non-preserved are indicated in black. The dashed blue and green lines indicate the thresholds $Z = 2$ and $Z = 10$, respectively. Most preservation Z statistics are able to distinguish preserved from non-preserved modules. The exception is separability which cannot distinguish preserved from non-preserved modules.

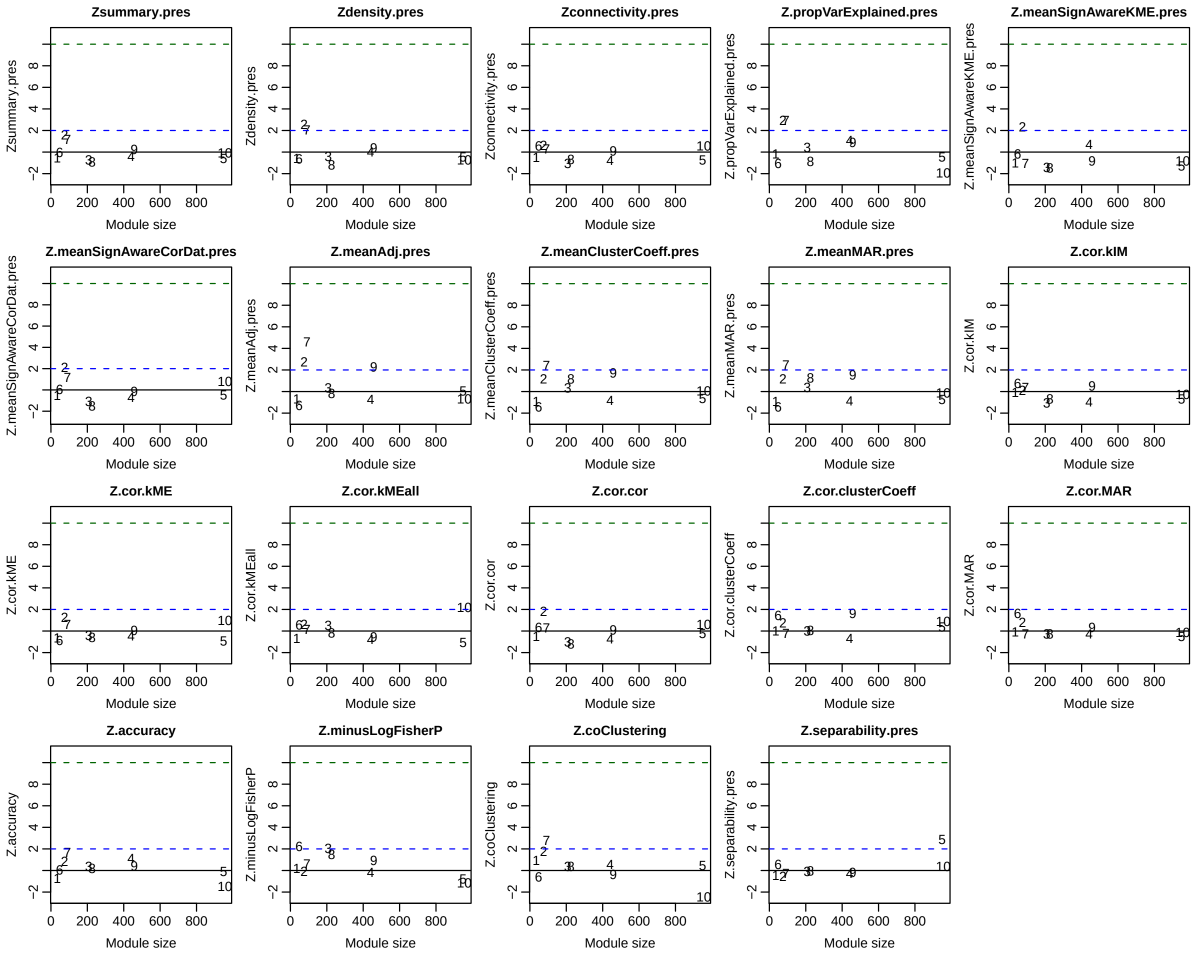

Figure 6. Module preservation Z statistics in the "Permuted" simulation as a function of module size. Modules are labeled by their numeric labels; modules simulated to be preserved between the reference and test data are indicated in red, while the ones simulated as non-preserved are indicated in black. The dashed blue and green lines indicate the thresholds $Z = 2$ and $Z = 10$, respectively. In this simulation none of the modules are simulated as preserved. Most of our preservation statistics show low preservation. This study highlights the utility of the summary Z statistics: although individual statistics may sometimes be above 2 (modules 2 and 7, statistics Z.propVarExplained.pres and Z.meanAdj.pres), the summary statistic is robust against such outliers.

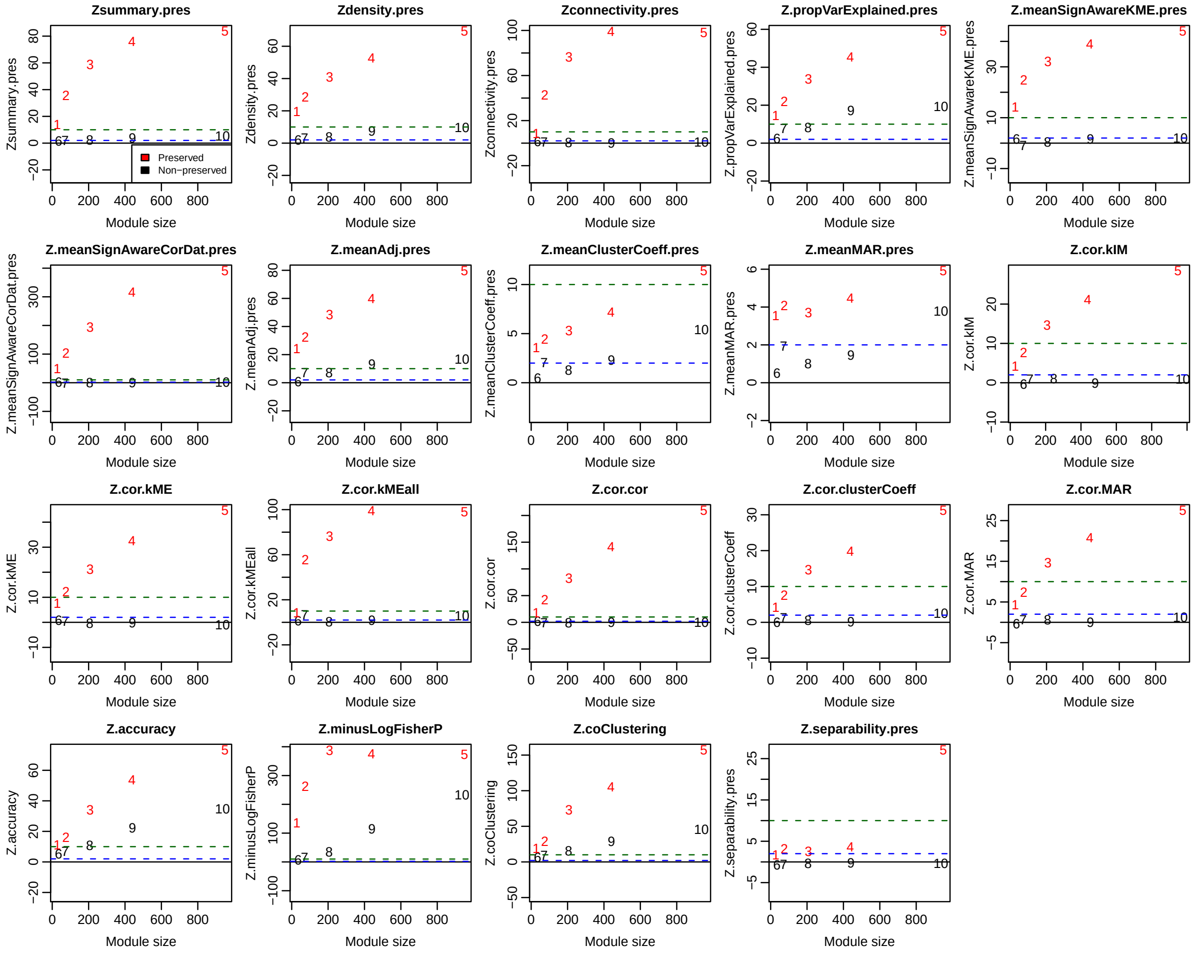

Figure 7. Module preservation Z statistics in the "Half-permuted" simulation as a function of module size. Modules are labeled by their numeric labels; modules simulated to be preserved between the reference and test data are indicated in red, while the ones simulated as non-preserved are indicated in black. The dashed blue and green lines indicate the thresholds $Z = 2$ and $Z = 10$, respectively. Here accuracy statistics fail to accurately distinguish preserved and non-preserved modules. In this study the connectivity statistics turn out to be the most reliable.

Comparison with co-clustering, cross-tabulation, and in-group proportion

In the Strong preservation simulation (half of the modules are preserved, denoted red in the plot) `clusterRepro` succeeds in separating most of the the preserved (IGP from about 0.3 to 0.9) and non-preserved modules (IGP between 0 and 0.3). The permutation p-values are also successful with the caveat that 10000 permutations were not enough to calculate meaningful p-values for the preserved module 5. Interestingly, the IGP for preserved modules seems to depend strongly on the size of the module, not unlike the dependence of our Z statistics on module size (Figure 1). The calculated p-values also appear to show a dependence on module size.

In the "Permuted" simulation (none of the modules are preserved), the actual IGP of some of the modules tends to be rather large (between 0.2 and 0.8), in the same range as for the preserved modules in the "Strong preservation" simulation. The p-values for several of the modules are again zero. Taking them at face value would suggest that the permutation p-values identify most non-preserved modules incorrectly as preserved.

In the "Half-permuted" the IGP does not separate preserved and non-preserved modules completely, since the IGP of the non-preserved modules 8 and 9 is higher than the IGP of the preserved module 2. Most of the p-values are non-zero and separate the preserved and non-preserved modules, with the exception of modules 2 (identified incorrectly as non-preserved) and modules 3,8,9 that have virtually equal p-values but module 3 is preserved and modules 8,9 are not preserved.

In summary, the IGP and the permutation procedure leading to the permutation p-values do not appear to reliably distinguish preserved from non-preserved modules. Further, the permutation procedure is inefficient in that a large number of permutations (likely in hundreds of thousands) would be necessary to arrive at p-values with sufficiently narrow confidence intervals. In our study we used 10000 permutations, taking nearly 10 hours of calculation on an 8-core workstation. However, the effective number of permutations used to calculate the p-value, varies for each module and was as low as 40.

5 Simulation 5: Simulation of non-preserved connectivity patterns

In this study we simulate a scenario similar to the half-preserved study, namely 10 modules labeled 1–10 in the reference set, and 5 modules labeled 1–5 in the test set. The genes in the reference and test modules 1–5 are the same, but their correlations r_i with the seed eigengene (Equation 1) are randomly permuted. Thus, the modules are preserved as groups (clusters) of highly correlated genes, but their intra-modular structure is not preserved. Thus, we expect that density measures will in general perform well, while connectivity measures will fail to distinguish the preserved and non-preserved modules.

Figure 8 summarizes the permutation Z statistics. We observe that while $Z_{density}$ separates preserved and non-preserved modules well, $Z_{connectivity}$ indicates no preservation. In this case, cross-tabulation statistics as well as IGP work well.

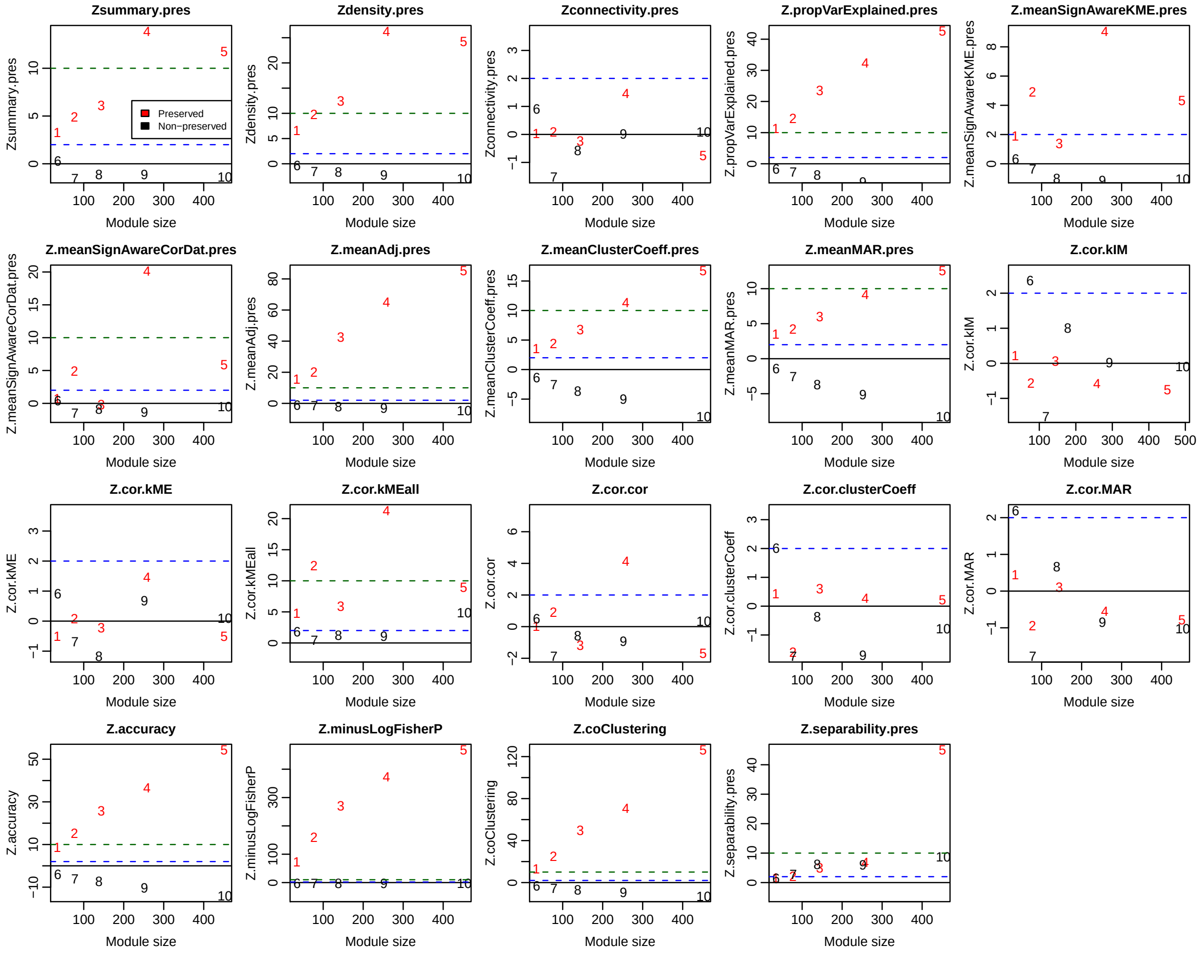

Figure 8. Module preservation Z statistics in the "In-module permuted" simulation as a function of module size. Modules are labeled by their numeric labels; modules simulated to be preserved between the reference and test data are indicated in red, while the ones simulated as non-preserved are indicated in black. The dashed blue and green lines indicate the thresholds $Z = 2$ and $Z = 10$, respectively. Here connectivity statistics fail to accurately distinguish preserved and non-preserved modules. In this study the density and cross-tabulation statistics work well.

6 Simulations 6–7: Simulation of pathways

In this study we simulate 10 clusters similar to study 4, 5 preserved and 5 non-preserved because their cluster membership in the test set in randomly permuted. However, on these simulations it is not the clusters that we are interested in. We form 5 modules 1–5 by randomly sampling genes from the preserved modules 1–5, and modules 6–10 by randomly sampling genes from the non-preserved modules. Thus, in this simulation the modules do not correspond to clusters, but the co-expression relationships among the genes in modules 1–5 are preserved, while the gene co-expression relationships among genes in modules 6–10 are not preserved.

Figures 9 and 10 summarize the permutation Z statistics. We observe that density statistics as well as IGP cannot distinguish the preserved and non-preserved "pathways" reliably. On the other hand, connectivity statistics distinguish preserved and non-preserved pathways with high accuracy.

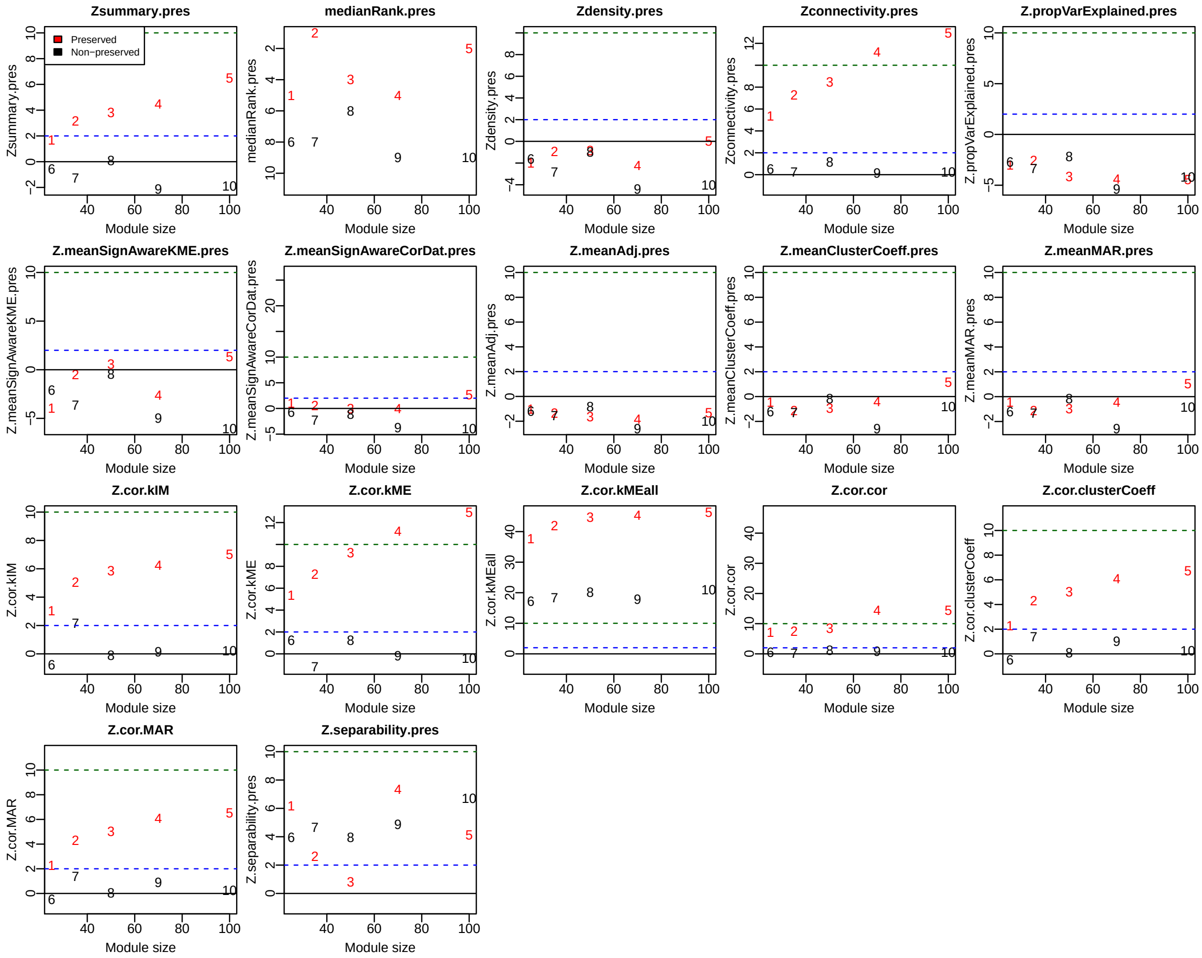

Figure 9. Module preservation Z statistics in the "Small pathway" simulation as a function of module size. Modules are labeled by their numeric labels; modules simulated to be preserved between the reference and test data are indicated in red, while the ones simulated as non-preserved are indicated in black. The dashed blue and green lines indicate the thresholds $Z = 2$ and $Z = 10$, respectively. Here connectivity statistics accurately distinguish preserved and non-preserved modules, while density statistics fail.

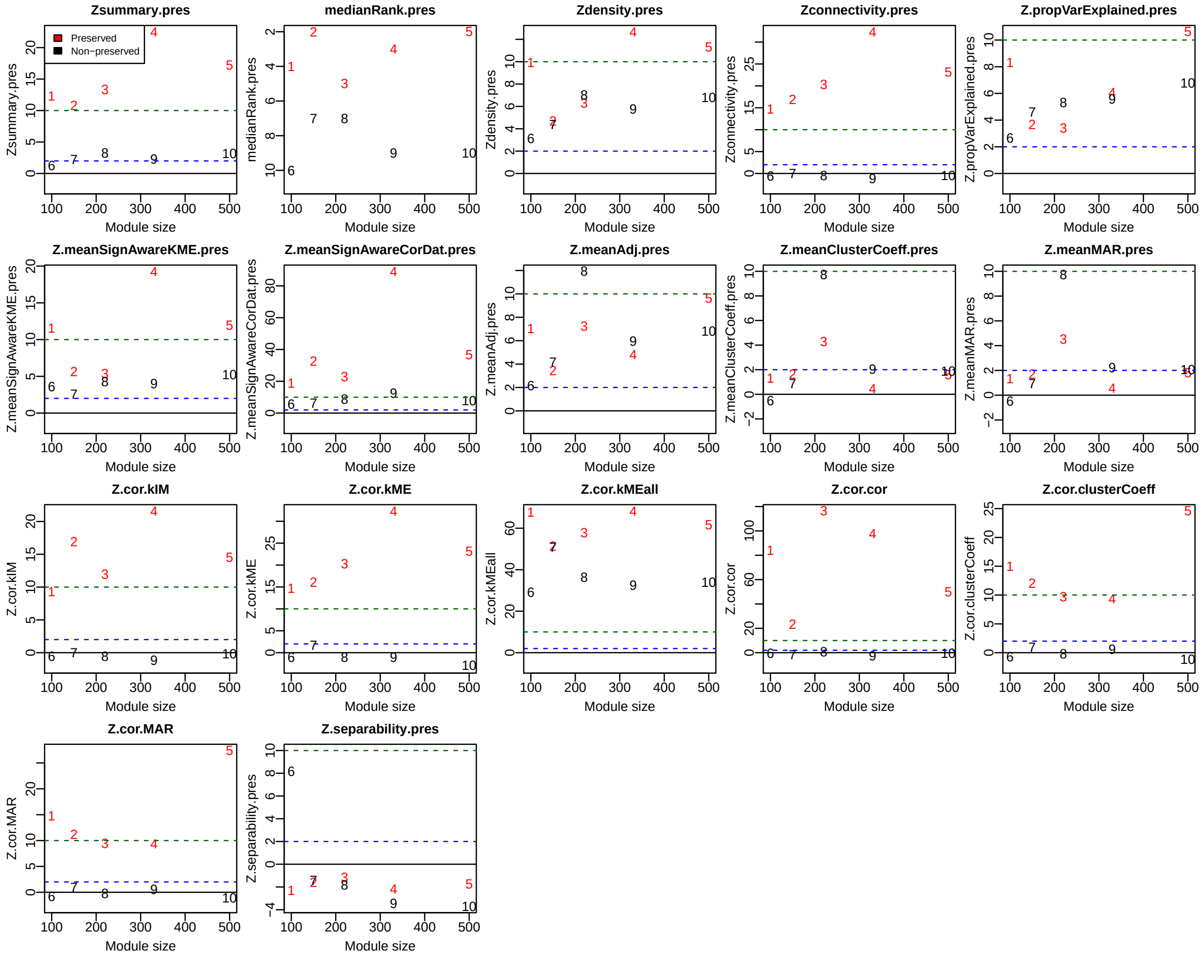

Figure 10. Module preservation Z statistics in the "Large pathway" simulation as a function of module size. Modules are labeled by their numeric labels; modules simulated to be preserved between the reference and test data are indicated in red, while the ones simulated as non-preserved are indicated in black. The dashed blue and green lines indicate the thresholds $Z = 2$ and $Z = 10$, respectively. Here connectivity statistics accurately distinguish preserved and non-preserved modules, while density statistics fail.

References

1. Kapp AV, Tibshirani R (2007) Are clusters found in one dataset present in another dataset? Biostatistics 8: 9-31.

2. Langfelder P, Horvath S (2008) WGCNA: an R package for weighted correlation network analysis. BMC Bioinformatics 9: 559.

3. Tibshirani R, Walther G (2005) Cluster validation by prediction strength. Journal of Computational and Graphical Statistics 14: 511–528.

4. Kaufman L, Rousseeuw P (1990) Finding Groups in Data: An Introduction to Cluster Analysis. New York: John Wiley and Sons, Inc.
